# Targeted Transcriptional Repression by Induced Proximity

**DOI:** 10.1021/acscentsci.5c02277

**Published:** 2026-06-11

**Authors:** Christian E. Stieger, Xinru Chen, Christina C. Kuismi, Dustin Dovala, Daniel Fuller, Andreas O. Frank, Mikias Woldegiorgis, Megan Bruce-Smythe, Fabian Wu, Nicolas Pizzato, Jeffrey McKenna, Cory Johannessen, Barna D. Fodor, Markus Schirle, Daniel K. Nomura

**Affiliations:** † Departments of Chemistry and Molecular and Cell Biology, 1438 University of California, Berkeley, Berkeley, California 94720, United States; ‡ Innovative Genomics Institute, Berkeley, California 94720, United States; § Molecular Therapeutics Initiative, Berkeley, California 94720, United States; ∥ Novartis-Berkeley Translational Chemical Biology Institute, Berkeley, California, 94720, United States; ⊥ Novartis BioMedical Research, Emeryville, California 94608, United States; # Novartis BioMedical Research, Cambridge, Massachusetts 02139, United States; ∇ Novartis BioMedical Research, Basel CH‑4056, Switzerland

## Abstract

Many transcription factors are considered “undruggable” and challenging targets due to the absence of ligandable pockets, large swaths of intrinsically disordered regions, and rapid turnover. Here, we describe a new induced-proximity therapeutic modality, **T**ranscriptional **R**epression via **A**ctive **C**hemical **E**pigenetic **R**eprogramming (TRACER), that enforces locus-specific transcriptional silencing by recruiting endogenous corepressor complexes to transcription factor binding sites. We developed small-molecule TRACERs that tether methyl-CpG binding domain protein 2 (MBD2), a component of the Nucleosome Remodeling and Deacetylase (NuRD) complex, to transcription factor-directed ligands. Recruitment of the NuRD complex by an estrogen receptor (ER) TRACER potently suppressed ER transcriptional activity in breast cancer cells, downregulated ER target genes, and required MBD2 and histone deacetylase (HDAC1/2) for activity, confirming on-target epigenetic repression. Extending this approach to prostate cancer, an androgen receptor (AR) TRACER transcriptionally repressed both full-length AR and the drug-resistant truncation variant, AR-V7, thereby achieving >90% inhibition of AR-dependent transcription in androgen-independent prostate cancer cells with locus-specific gene repression. Collectively, these findings establish TRACERs as a generalizable modality to pharmacologically silence transcription factors through targeted epigenetic reprogramming, offering a powerful strategy for treating cancers refractory to existing therapies.

## Graphical abstract

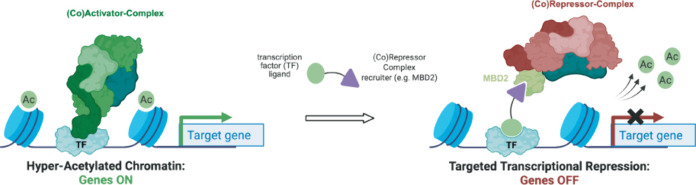

## Introduction

Despite the discovery of a large number of potential therapeutic targets for cancer, a critical bottleneck in realizing new cancer cures that exploit our expanding knowledge of cancer is the daunting realization that most proteins, over 90%, are still considered “undruggable.”^1^ This is because most proteins do not possess the classical binding pockets often found in enzyme active sites, but rather function through protein–protein or protein-nucleic acid interactions, or are highly intrinsically disordered or unstructured.^2–5^ Alongside advances in mapping ligandable sites and in enabling ligand discovery against undruggable targets with technologies such as activity-based protein profiling or DNA-encoded libraries, there has also been a complementary explosion in induced-proximity-based therapeutic modalities, ranging from targeted protein degradation to many additional approaches to manipulate post-translational modifications and protein function by recruiting an effector to a target protein of interest.^1,6,7^ However, existing drug discovery technologies and modalities still have gaps in addressing remaining challenges associated with difficult-to-drug targets. One of the most challenging, yet therapeutically critical, classes of proteins known to drive many human cancers are transcription factors.^3,4^ Transcription factors, such as MYC, CTNNB1, androgen receptors (AR and truncation variant AR-V7), estrogen receptors (ER), YAP and TAZ, STAT3, and many others, are known to drive cancer pathogenesis through engaging their respective promoters at genomic loci to activate the transcription of genes involved in cell proliferation, survival, and metastasis.^8–12^ While some of these transcription factors have been pharmacologically targeted and even degraded with newer therapeutic modalities such as targeted protein degradation (TPD) (e.g., ER and AR),^13^ many of these transcription factors have been more difficult to target directly. Furthermore, transcription factors pose an additional challenge, as many of these proteins have very rapid turnover rates, particularly in cancer cells, where they are rapidly degraded and resynthesized, making them difficult to target with classical inhibitors or even innovative degraders in a durable manner. Alternative efforts to epigenetically target these transcription factors by broadly modulating chromatin remodeling through histone modification (e.g., HDAC1/2, CBP/EP300, SMARCA2/4, and BRD4 inhibitors and degraders^9–11,14–16^ ) have thus far not achieved a viable therapeutic window.

An ideal therapeutic strategy would be to directly target transcription factors and epigenetically silence the genetic loci associated with them, rather than broadly targeting multiple transcriptional loci. While epigenome editing using dCas9/gRNA fusions with DNA methyltransferases has enabled locus-specific epigenetic silencing, these large protein-nucleic acid complexes are not practical for systemic and comprehensive delivery to most cancer types.^17^ Recently, the Gray and Crabtree groups have reported on a small molecule-based targeted transcriptional activation or rewiring approaches with transcriptional/epigenetic chemical inducers of proximity (TCIPs) wherein they developed heterobifunctional molecules to recruit BRD4, CDK9, or lysine acetyltransferases to BCL6 transcriptional loci to activate BCL6 genetic loci and repress MYC.^18–20^ In this study, we have developed a novel, induced-proximity-based therapeutic modality for targeted transcriptional repression, termed **T**ranscriptional **R**epression via **A**ctive **C**hemical **E**pigenetic **R**eprogramming (TRACER). TRACERs consist of a transcriptional corepressor complex recruiter linked to a transcription factor targeting ligand to epigenetically and locus-specifically repress transcription. We demonstrate proof-of-concept of the TRACER platform through the recruitment of methyl CpG binding domain protein 2 (MBD2) and the nucleosome remodelling and deacetylase (NuRD) corepressor complex to estrogen receptor (ER) and androgen receptor (AR) genetic loci in breast and prostate cancer cells, respectively.

## Results

### Characterization of an MBD2 Inhibitor as an MBD2 Recruiter for TRACER Applications

MBD2 functions as a core subunit of the NuRD complex, where it binds methylated DNA and recruits other NuRD components—including histone deacetylases (HDAC1/2), CHD3/4, RBBP4/7, and MTA family proteins—to coordinate chromatin remodeling and histone deacetylation, ultimately leading to transcriptional repression.^21^ MBD2 may also recruit additional repressors, including PRMT5 and SIN3A-associated corepressor complexes, although these interactions are more context-specific and less universally conserved than NuRD.^22^ To enable the TRACER strategy, we hypothesized that we could utilize MBD2 as a general recruiter of corepressor complexes to facilitate targeted transcriptional repression (Figure 1a). We identified a previously reported MBD2 ligand, KCC-07, which has been reported to block the interaction between MBD2 and hypermethylated DNA both *in vitro* and *in situ* with a reported 50% inhibitory concentration (IC_50_) of 1.6 μM, as a promising chemical starting point for developing TRACERs (Figure 1b).^23–25^ When we tested the binding of this ligand with the purified methyl-binding domain of MBD2 by nuclear magnetic resonance (NMR) spectroscopy, we were surprised to observe no binding in either the presence or absence of nucleic acids (Figure S1a–d). We observed chemical shift perturbations in MBD2 upon nucleic acid binding, indicating that the protein was properly folded and could bind DNA (Figure S1a). We thus postulated that perhaps this KCC-07 ligand binds to MBD2 only in a cellular context and might require MBD2 interaction with its other NuRD complex partners for binding. To test the binding of this ligand in cell lysates, we generated a biotin-functionalized probe based on KCC-07, CS-1-174, as well as a negative control biotin-PEG probe (Figure 1b, Figure S2a). In both Ramos and 22Rv1 cancer cell lysates, we demonstrated robust enrichment of MBD2, but not of the unrelated target tubulin, with CS-1-174 compared to the negative control probe (Figure 1c,d). In order to further demonstrate MBD2 engagement in living cells, we also prepared an alkyne-functionalized photoaffinity probe, CS-1-102, as well as a negative control diazirine-phenyl (diazirine-Ph) probe, and demonstrated significant enrichment and engagement of MBD2 in Ramos cells compared to the negative control (Figure 1b,e, Figure S2a). Using an orthogonal affinity selection mass spectrometry (ASMS) approach for assessing binding *in vitro*, we also demonstrated that while KC-007 and its related analog CS-1-69 do not show binding to the individual NuRD components MBD2 or HDAC1, we observed robust dose-responsive binding when MBD2, HDAC1, and MT1A proteins were in complex (Figure 1b,f–g). We could not purify MT1A alone to test ligand binding in the absence of its complex partners. Collectively, these experiments indicated that while KC-007 does not bind to the methyl binding domain of MBD2 alone, it does bind to MBD2 in complex cellular proteomes, in cells, and *in vitro* as an assembled NuRD complex. While this relatively simple ligand is likely to possess additional targets beyond MBD2, we nonetheless sought to demonstrate proof-of-concept of the TRACER platform with the hope that we could assess the on-target mode of action with subsequent genetic validation studies.

**Figure 1:**
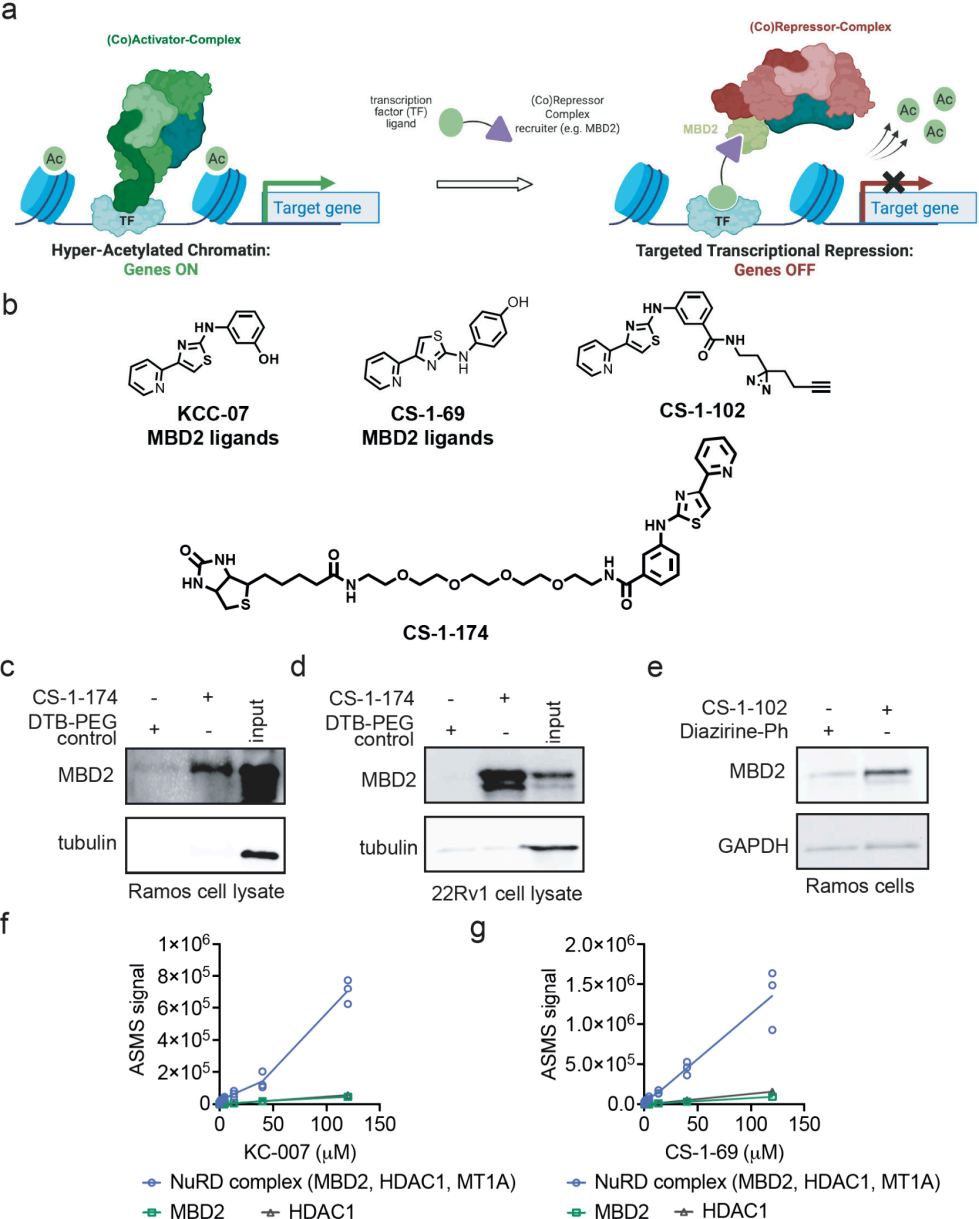
Targeted Transcriptional Repression with TRACERs using an MBD2 recruiter. (a) Targeted transcriptional repression with TRACERs that consist of an epigenetic recruiter (e.g., against MBD2) linked to a transcription factor targeting ligand to recruit a corepressor complex to specific transcriptional loci to repress transcription. (b) Structures of MBD2 inhibitors KCC-07 and CS-1-69, and derivative photoaffinity and biotin probes. (c, d) MBD2 enrichment from Ramos (c) and 22Rv1 (d) with KCC-07-based biotin probe, CS-1-174. Cell lysates were incubated with streptavidin beads pretreated with CS-1-174 or a negative control biotin-PEG probe (2 h, 4 °C), and input and pulldown MBD2 and unrelated protein tubulin levels were assessed by SDS/PAGE and Western blotting. (e) MBD2 cellular engagement. Ramos cells were treated with a negative control diazirine-Ph probe or CS-1-102 (25 μM, 2 h), after which cells were irradiated, and cell lysates were subjected to copper-catalyzed azide–alkyne cycloaddition (CuAAC) with a biotin-functionalized azide handle, after which probe-labeled proteins were avidin-enriched, eluted, and MBD2 and unrelated protein GAPDH levels were assessed by SDS/PAGE and Western blotting. (f, g) ASMS analysis of KC-007 or CS-1-69 binding to MBD2, HDAC1, or MBD2, HDAC1, and MT1A proteins in the NuRD complex. Protein(s) (7.5 pmol) were incubated with compound for 1 h, and binding was analyzed by ASMS. Data shown in (c–g) are from *n* = 3 biologically independent replicates per group. Blots in (c–e) are representative. Data in (f, g) are individual replicate values and the average.

To assess the initial feasibility of the TRACER platform, we selected estrogen receptor (ER) as a target in breast cancer cells using an ER agonist, estrone. Given the precedent that functionalized estrogens can retain the ability to bind and even activate ER, we sought to use the oxime-linked estrone derivative CS-1-65 as an ER ligand (Figure 2a).^19,26^ Analogously to the two naturally occurring estrogens, estrone (E1) and estradiol (E2), the linker-functionalized estrone derivative CS-1-96 induced ER-mediated transcription, indicating robust agonistic activity (Figure S2c).

**Figure 2:**
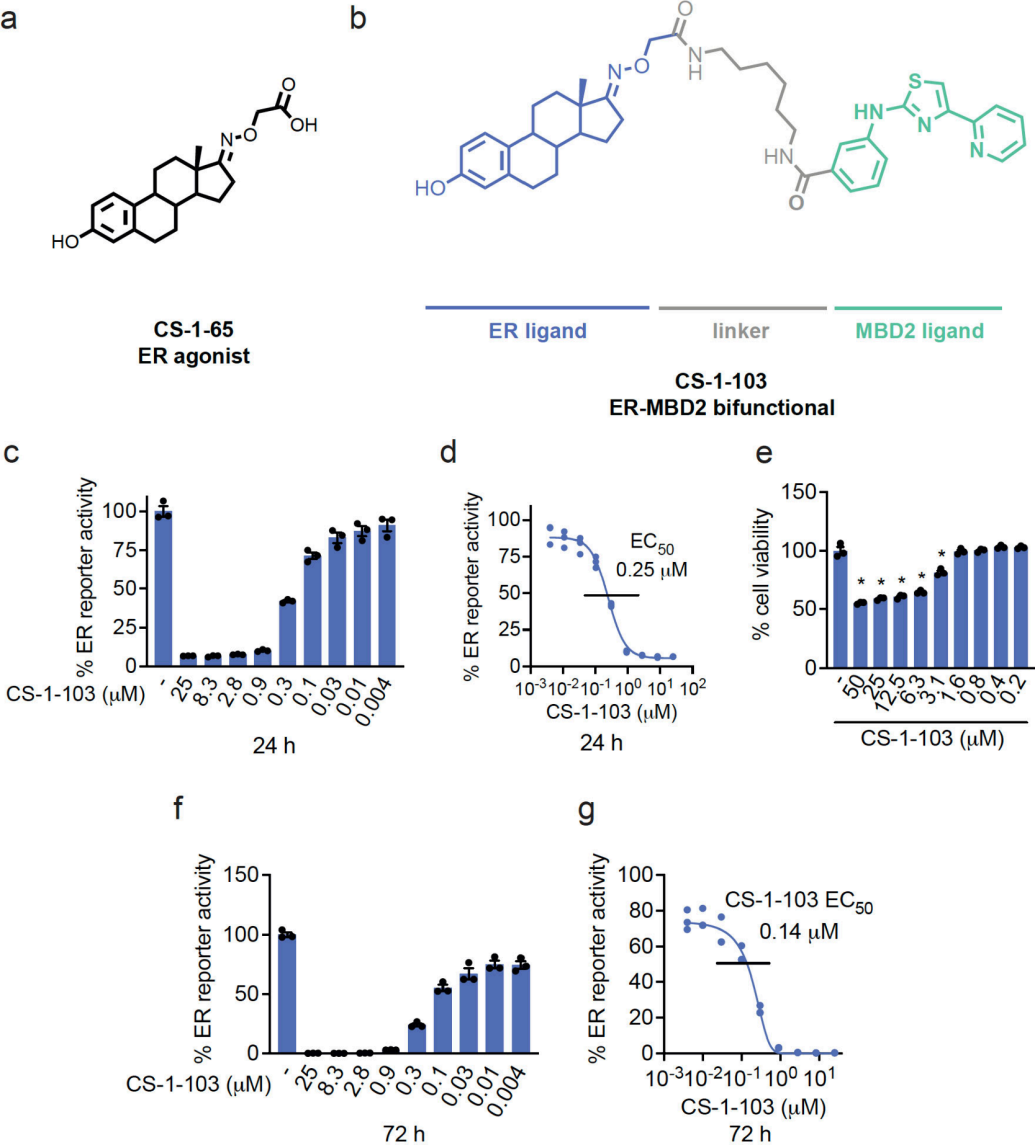
ER TRACER CS-1-103. (a) Structure of estrone derivative ER agonist, CS-1-65. (b) Structure of ER TRACER CS-1-103. (c, d) Dose-responsive inhibition of ER luciferase transcriptional reporter activity in T47D cells upon treatment with DMSO vehicle or TRACER CS-1-103 for 24 h, showing an EC_50_ of 250 nM. (e) 24 h cell viability by Cell TiterGlo in T47D cells showing an EC_50_ > 50 μM. (f, g) Inhibition of T47D ER luciferase reporter activity after treatment with DMSO vehicle or CS-1-103 treatment for 72 h, showing an EC_50_ of 320 nM. Shown in (c–g) are individual replicate values and the average (d, g) or average ± SEM (c, e, f).

### Development and Characterization of an ER TRACER

We subsequently synthesized heterobifunctional MBD2 ligand-based TRACERs targeting ER, each containing a variety of linkers, and showed that many of these molecules robustly and potently inhibited ER transcriptional activity in T47D breast cancer cells, with minimal overall impairment in cell viability (Figure S3a,b, Figure 2b–e, Figure S4a–c). Among these compounds, CS-1-103, CS-1-163, and CS-1-86 showed the best potency, with 50% effective concentrations of 0.25, 0.89, and 1.3 μM, respectively (Figure 2b–e, Figure S3a,b, Figure S4a–c). We did not observe a “hook effect” with these heterobifunctional molecules, but we believe this is because we were unable to test higher concentrations due to solubility issues. The inhibition of ER transcriptional activity by the more potent compound CS-1-103, observed after 24 h of treatment, was even more pronounced after 72 h of treatment with an EC_50_ of 0.14 μM (Figure 2f,g). To assess the durability of ER transcriptional inhibition induced by CS-1-103, we performed a washout experiment comparing it with the clinically approved selective estrogen receptor degrader (SERD), fulvestrant. We treated T47D cells with each compound for 72 h and assessed ER transcriptional activity with and without a 24 h washout period (Figure S4d,e). While the 50% inhibitory concentration (IC_50_) of fulvestrant was shifted by 23-fold with washout, we observed only a 1.4-fold shift with CS-1-103 (Figure S4d–e), indicating greater durability of inhibition after compound washout with our TRACER compared to a SERD. To further demonstrate that the individual components of our TRACER either alone or in combination could not exert similar inhibitory effects, we showed that treatment of T47D cells with KCC-07, CS-1-65, CS-1-96, and KC-007, E1, or E1 and KC-007 treatments either stimulated or did not alter ER transcriptional activity (Figure S5a–e). The stimulation of ER transcriptional activity with KCC-07 treatment is likely due to global derepression of transcriptional responses, including ER transcriptional activity, consistent with previous reports using KCC-07.^23,25^ We presume that the inhibition observed at the highest concentration is likely due to off-target toxicity. The lack of agonist activity of CS-1-65 or E1 in T47D cells is likely due to the presence of hormones, including estrogens, in cell culture serum, since we observe robust ER transcriptional activity in T47D cells with CS-1-96 or E1 in charcoal-stripped media, which does not contain lipophilic compounds, including estrogens and other steroids (Figure S2c).

To demonstrate the necessity of the MBD2 recruiter in the observed activity, we showed complete attenuation of CS-1-103- and CS-1-86-mediated ER inhibitory activity with cotreatment with KCC-07 or CS-1-69 (Figure 3a, Figure S6a). To demonstrate on-target activity through recruitment of HDACs and the NuRD complex, we also showed significant attenuation of CS-1-103- and CS-1-86-mediated ER inhibition by the HDAC1/2-selective inhibitors Mocetinostat and CI-944 (Figure 3b, Figure S6b). These data indicate that HDAC activity was required for TRACER-mediated inhibition of ER transcriptional activity, rather than other potential mechanisms, such as disruption of ER protein complexes or sequestration of ER from its native genetic loci. Most importantly, we demonstrated significant rescue of CS-1-103- and CS-1-86-mediated ER inhibitory activity upon MBD2 knockdown, consistent with on-target activity. Importantly, MBD2 knockdown alone did not substantially affect ER-driven reporter gene activity (Figure 3c,d, Figure S6c,d). While these data were promising, our readout used an artificial luciferase reporter system; thus, we sought to confirm this ER transcriptional inhibitory activity with endogenous ER gene targets. We showed that CS-1-103 treatment significantly downregulated ER target genes, including ER (ESR1) itself and GREB1, both under basal conditions with serum containing native steroid levels and in charcoal-stripped medium with added exogenous estrogen E2 after 72 h (Figure 4a,b). We also observed that ER protein levels were downregulated by CS-1-86 treatment, but ER transcriptional activity was inhibited earlier than ER protein levels in a time-course study, indicating that the loss of ER protein levels was likely due to transcriptional inhibition and ER transcriptional downregulation rather than ER degradation (Figure S6e–g). To further confirm that the CS-1-103-mediated transcriptional inhibitory activity was not occurring through proteasomal degradation-mediated pathways, we further demonstrated that the CS-1-103-mediated ER transcriptional inhibitory activity was not attenuated with pretreatment of cells with a proteasome inhibitor, bortezomib (Figure S4h). In accordance with our observations in the luciferase reporter system, we further demonstrated significant attenuation of ER target gene downregulation upon MBD2 knockdown, while tamoxifen-mediated inhibition of ER target genes was not attenuated by MBD2 knockdown (Figure 4c,d, Figure S4i).

**Figure 3:**
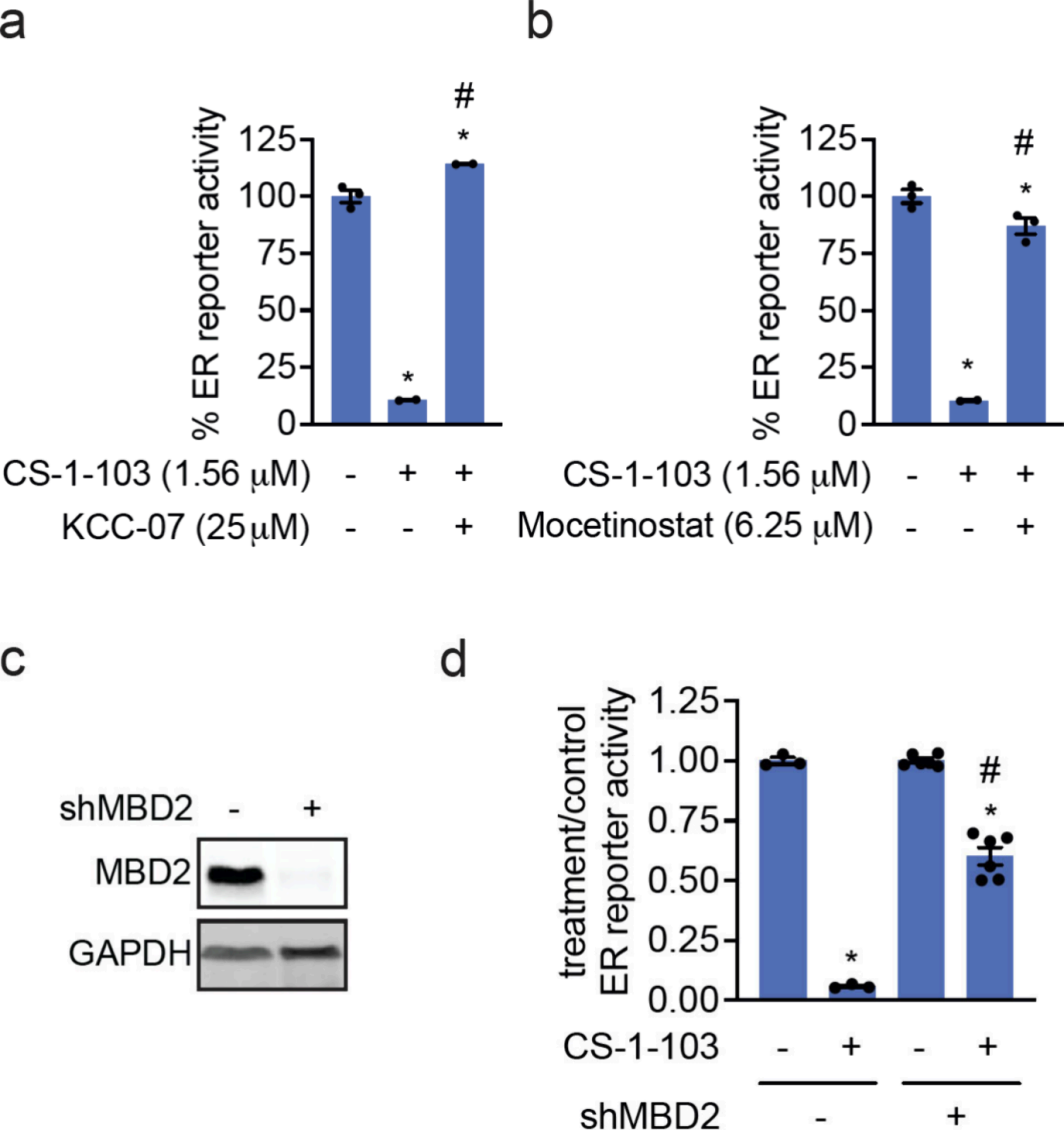
Elucidating the mechanism of ER TRACER. (a, b) Attenuation of CS-1-103-mediated ER luciferase reporter inhibition in T47D cells with KCC-07 or HDAC inhibitor Mocetinostat. ER luciferase reporter T47D cells were cotreated with DMSO vehicle, KCC-07 (a), or pretreated with Mocetinostat (b) for 1 h prior to treatment of cells with DMSO vehicle or CS-1-103 for 24 h, after which ER luciferase transcriptional activity was read out. (c) Stable short hairpin RNA (shRNA) knockdown of MBD2 in T47D cells assessed by SDS/PAGE and Western blotting alongside loading control GAPDH. (d) Attenuation of CS-1-103-mediated inhibition of T47D ER luciferase reporter activity upon MBD2 knockdown. T47D shControl versus shMBD2 cells were treated with DMSO vehicle or CS-1-103 (1.56 μM) for 24 h, after which ER luciferase transcriptional activity was read out. Treatment data for shControl and shMBD2 cells are in relation to the respective vehicle-treated controls for shControl and shMBD2 cells, respectively. Data in (a–d) are from *n* = 3–6 biologically independent replicates per group. Data in (a, b, d) show individual replicate values and average ± SEM. Blot in (c) is representative. Significance is expressed as *p < 0.05 compared to vehicle-treated control and #*p* < 0.05 compared to CS-1-103-treated groups in (a, b) and CS-1-103-treated shControl cells in (d).

**Figure 4:**
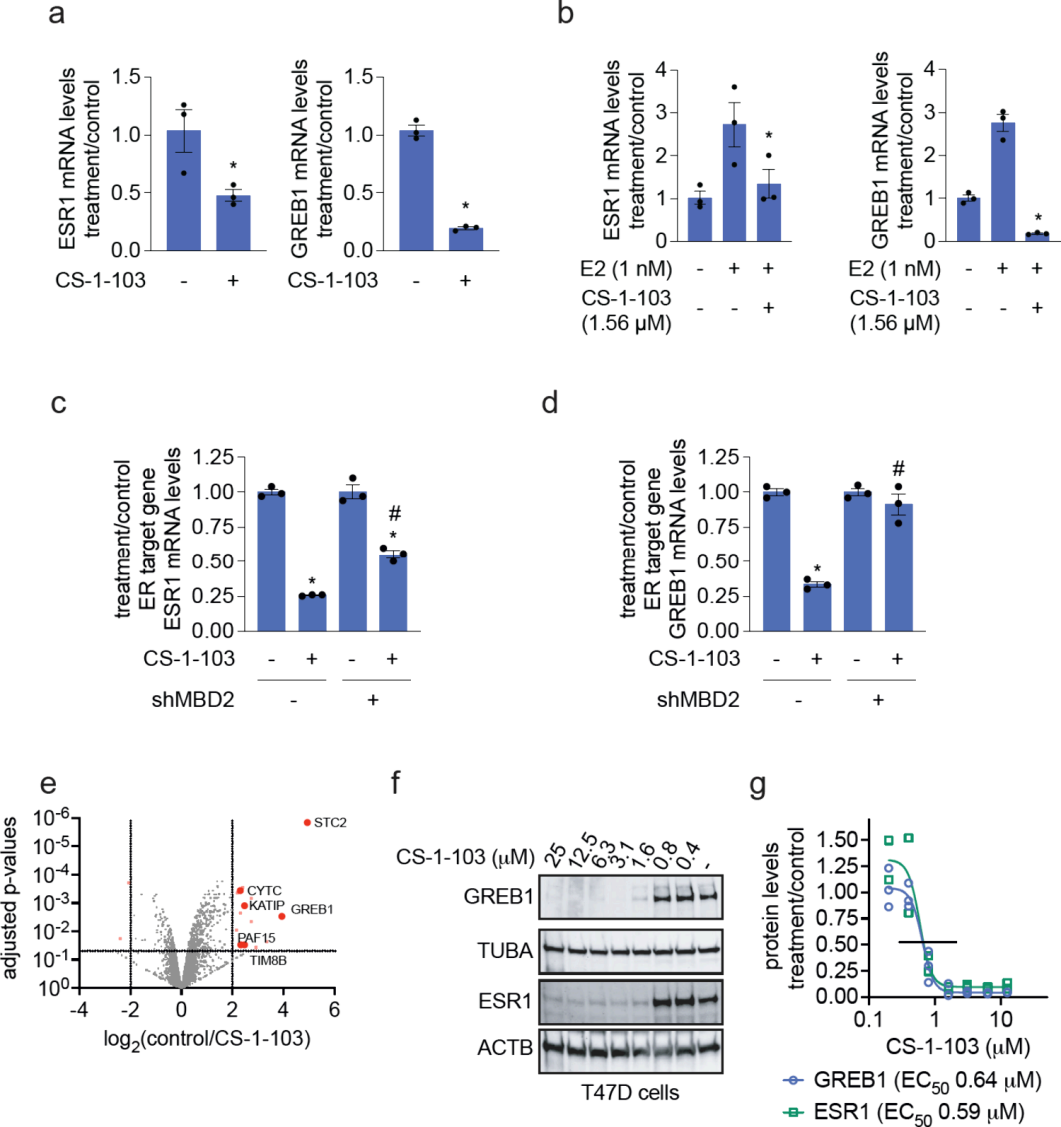
Characterization of ER TRACER. (a) RT-PCR of ER target genes with CS-1-103 treatment. T47D cells grown in normal serum were treated with DMSO vehicle or CS-1-103 (1.56 μM) for 24 h, after which ESR1 and GREB1 mRNA levels were assessed by RT-PCR. (b) RT-PCR of ER target genes with CS-1-103 treatment. T47D cells were grown under charcoal-stripped serum for 72 h, followed by cotreatment with E2 (1 nM) and DMSO vehicle or CS-1-103 (1.56 μM) for 24 h, and ESR1 and GREB1 mRNA levels were assessed by RT-PCR. (c, d) Attenuation of CS-1-103-mediated downregulation of ER target genes upon MBD2 knockdown. T47D shControl and shMBD2 cells were treated with DMSO vehicle or CS-1-103 (1.56 μM) for 24 h, and ESR1 (c) and GREB1 (d) mRNA levels were assessed by RT-PCR. Treatment data for shControl and shMBD2 cells are in relation to the respective vehicle-treated controls, respectively. (e) Proteomic analysis of T47D cells treated with DMSO vehicle or CS-1-103 (12.5 μM) for 24 h. (f, g) Dose–response of ER target protein downregulation with CS-1-103 treatment. T47D cells grown in normal serum were treated with DMSO vehicle or CS-1-103 for 24 h, after which ESR1 and GREB1 protein levels were assessed by SDS/PAGE and Western blotting (f) and quantified (g). Data in (a–g) are from *n* = 3 biologically independent replicates per group. Data shown in (a–d) show individual replicate values and the average (a, g) or average ± SEM (b–d). Significance is expressed as *p < 0.05 compared to vehicle-treated controls and #*p* < 0.05 compared to CS-1-103-treated shControl groups.

Quantitative proteomics revealed a significant reduction in the abundance of proteins encoded by ER-target genes after 24 h of treatment (Figure 4e; Table S1). We further confirmed dose-responsive reductions in GREB1 and ER protein levels with CS-1-103 treatment, with EC_50_ values of 0.64 and 0.59 μM, respectively, comparable to those observed in ER luciferase reporter assays (Figure 4f,g). Transcriptomic profiling by RNA-seq showed robust repression of various ER target genes and revealed high selectivity for genes associated with estrogen response (Figure 5a–c, Figure S7a; Table S2). Moreover, comparing the RNA-seq data with publicly available transcription factor ChIP-seq experiments revealed strong enrichment for the ESR1 and ESR2 data sets (Figure 5d, Figure S7b,c). We also performed RNA-seq analysis of CS-1-103 treatment in MBD2 knockdown T47D cells and observed an overall transcriptionally silent signature, with no significant alterations in ER or other pathways (Figure 5e–g; Table S3), strongly indicating that the CS-1-103-mediated transcriptional changes were MBD2-dependent.

**Figure 5:**
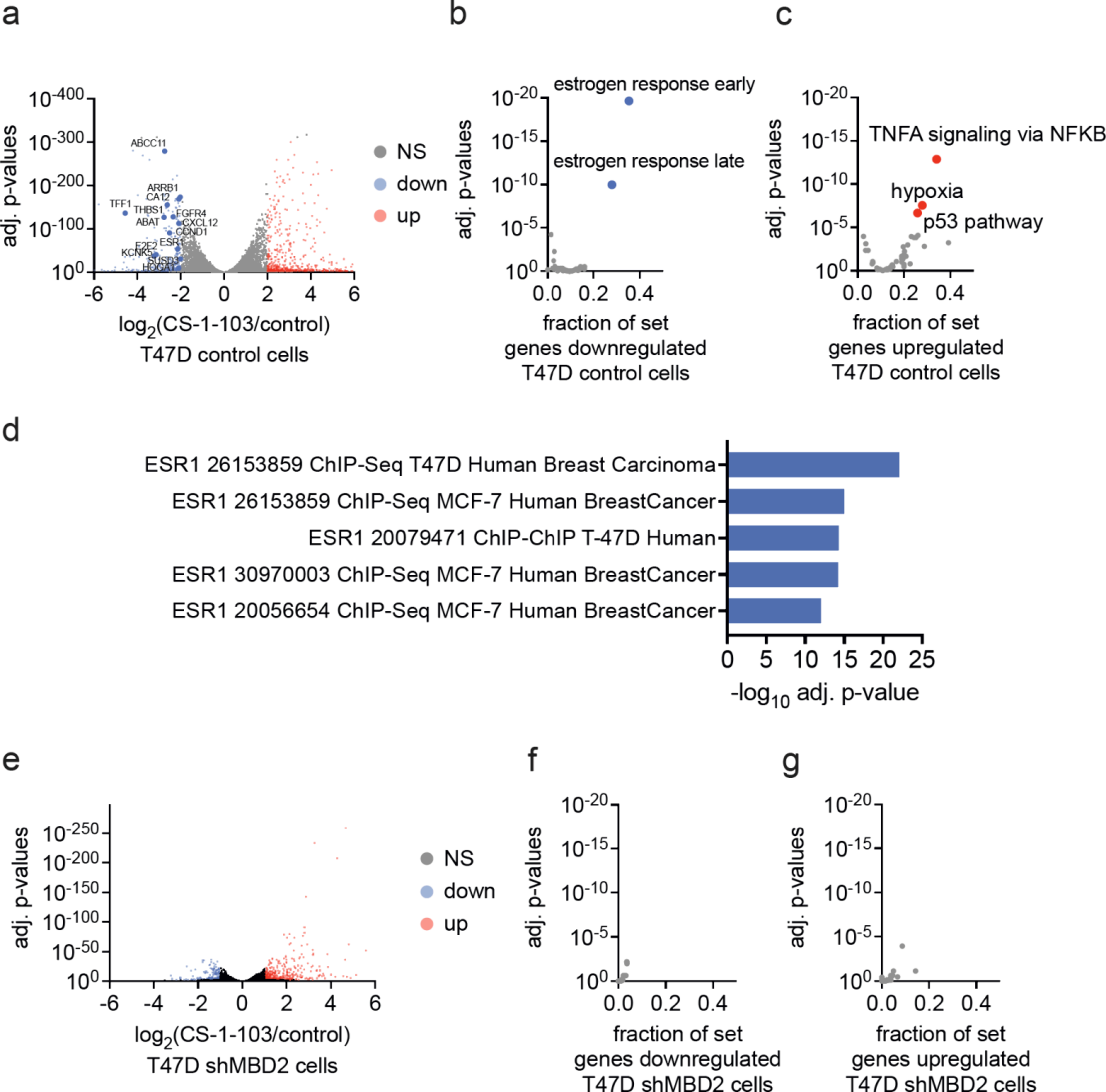
Transcriptomic profiling of ER TRACER (a) RNA-seq transcriptomic data of T47D cells cotreated with 1 nM E2 and DMSO vehicle or CS-1-103 (1.56 μM) for 24 h. (b–d) Comparison of significantly down-regulated genes to the ChEA 2022 transcription factor targets. (e) RNA-seq transcriptomic data of T47D shMBD2 cells cotreated with 1 nM E2 and DMSO vehicle or CS-1-103 (1.56 μM) for 24 h. (f, g) Comparison of significantly down-regulated genes to the ChEA 2022 transcription factor targets. Data are from *n* = 3 biologically independent replicates per group. Data from RNA-seq experiments are available in Tables S2 and S3.

To further interrogate the chromatin and transcriptional consequences of CS-1-103 treatment, we performed Assay for Transposase-Accessible Chromatin with sequencing (ATAC-seq^27^) in T47D cells. More than 50% of ER-bound ATAC-seq peaks were localized to promoter regions (). We then integrated the promoter-centric data with RNA-seq and ER-binding profiles. Motif analysis revealed that CS-1-103-responsive downregulated ATAC-seq peaks were strongly enriched for canonical ER response elements, as well as motifs for cooperating transcription factors including NFY, FOXA, and TEAD family members (Figure 6a–c, Table S4). Further analysis of the promoter regions associated with changes in either DNA accessibility or RNA expression revealed that these changes were not exclusive to ER-bound elements and were inconsistent with repression. These could reflect both indirect downstream effects and differences in additional regulatory mechanisms modulating the response to compound treatment (Figure 6b). We also assigned nonpromoter elements to the nearest genes and found the results consistent with the promoter-centric observations (Figure S9). We then next focused on only the subset of these promoters that had overlapping ER peaks and asked whether promoters with changes consistent with repression (ATAC-seq and/or RNA expression downregulated) had any sequence features distinguishing them from the other categories (Figure 6c). The ERβ motif (ERB) was enriched in the ATAC-seq and/or RNA-expression down categories and depleted in the opposite categories. The opposite was observed for transcription factor binding motifs such as MITF and LHX6. These results suggest that CS-1-103-dependent transcriptional repression at ER-bound loci may be context-dependent.

**Figure 6:**
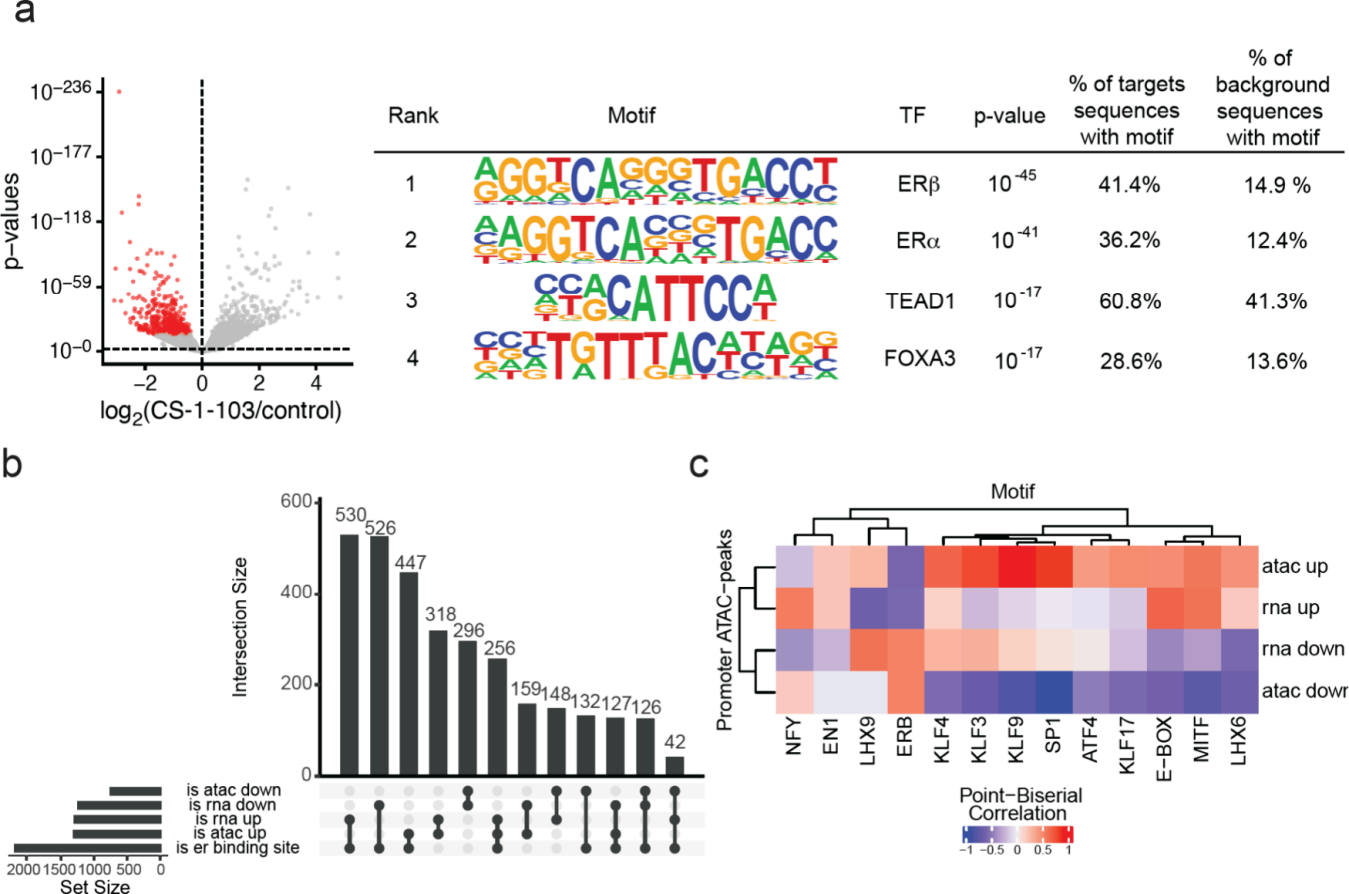
Integrative chromatin accessibility and transcriptional profiling of CS-1-103 TRACER activity in T47D cells. (a) Motif enrichment analysis of CS-1-103-responsive regions (500 downregulated ATAC-seq peaks with the highest p-values, see left panel), confirming top enrichment of canonical estrogen receptor binding motifs, along with TEAD and FOXA motifs (right panel). ATAC-seq data are from *n* = 3 biologically independent replicates per group. (b) UpSet plot depicting the number of promoters with a change in accessibility (measured by ATAC-seq) or/and RNA expression with or without estrogen receptor peaks (measured by CUT&RUN). (c) Point-biserial correlation heatmap relating transcription factor motif enrichment to estrogen receptor target promoters from (b). Motif enrichment is represented as the log_2_(odds ratio) from HOMER analysis. Peak categories are defined by significant changes in chromatin accessibility (ATAC up/down) and gene expression (RNA up/down). After CS-1-103 treatment, the ERβ (ERB) motif is enriched in the promoters with downregulated ATAC-seq peaks and RNA-expression, while it is depleted in promoters with upregulated ATAC-seq and RNA-expression, respectively. ATAC-seq data are available in Table S4.

### Development and Characterization of an AR TRACER

While ER was a promising proof-of-concept for our TRACER platform that may be applicable to certain so far intractable ER mutants, existing ER-targeting drugs, such as SERDs, Fulvestrant, and recently approved ER PROTACs, are likely to successfully tackle a large fraction of ER+ breast tumor patients.^28^ We therefore sought to apply the TRACER modality to a currently unmet medical need: androgen-independent prostate cancers resistant to AR antagonists and current clinical AR PROTACs due to the expression of truncation variants, such as constitutively activated AR-V7, which lacks the ligand-binding domain, alongside full-length AR.^10,29^ While AR-V7 has been challenging to target directly due to its high intrinsic disorder, both AR and AR-V7 still bind to the same genetic loci. As such, epigenetic suppression of AR loci with an AR TRACER would also repress AR-V7 loci by blocking AR-V7 access to the common chromatin binding sites. To develop AR TRACERs, we linked a selective androgen receptor modulator (SARM) ligand RU59063 to our MBD2 recruiter using linkers of various lengths and composition to generate five AR/AR-V7 TRACERS—CS-1-162, CS-1-168, CS-1-169, CS-1-175, and CS-1-176 (Figure S10a). As expected, RU59063 alone inhibited AR transcriptional activity by about only about 40% in 22Rv1 cells expressing both AR and AR-V7, since RU59063 binds to the ligand-binding domain of AR (Figure 7a,b). Upon testing the five AR and AR-V7 TRACERs in 22Rv1 AR transcriptional reporter assays, we found several compounds, including CS-1-162, CS-1-168, CS-1-175, and CS-1-176, that robustly inhibited total AR transcriptional activity by >90% in a dose-responsive manner with EC_50_ values of 1.3, 4.7, 1.3, and 2.5 μM, respectively, while CS-1-169 only showed modest inhibition comparable to RU59063 (Figure S10b). However, CS-1-162, CS-1-168, and CS-1-176 showed substantial impairments in cell viability, although their cytotoxic effects were less potent than their transcriptional inhibitory effects (Figure S10b). In contrast, CS-1-175 showed >90% inhibition of total AR luciferase reporter transcriptional activity in 22Rv1 cells with minimal cytostatic effects (Figure 7c–e; Figure S10b). We also observed more durable inhibition of AR transcriptional activity in a time-course study comparing RU59063 treatment to CS-1-175 (Figure S11a). To confirm that these effects were not due to the MBD2 ligand or RU59063 alone or in combination, we demonstrated that KC-007 or cotreatment with KC-007 and RU59063 combined treatment had no effect on AR transcriptional activity in 22Rv1 cells (Figure S11b,1c). CS-1-175 treatment also did not lower AR or AR-V7 protein levels in 22Rv1 cells (Figure S11d). In addition to the reporter system, CS-1-175 also significantly downregulated endogenous AR target genes (Figure 7f). This inhibition of AR transcriptional activity was attenuated by preincubation with an HDAC inhibitor, and the downregulation of AR target genes was completely attenuated upon MBD2 knockdown, indicating on-target activity (Figure 7g–i). HDAC inhibitor treatment alone in 22Rv1 cells did not increase basal AR transcriptional activity, but rather inhibited activity at higher concentrations, demonstrating that it did not confound the effects of our rescue experiments (Figure S11e). Transcriptomic profiling of CS-1-175 by RNA-seq showed significant downregulation of many AR target genes (Figure 8a; Figure S12b; Table S5). Analyzing effects on genes that have been previously shown to be regulated by AR-V7,^30^ CS-1-175 universally and significantly affected almost every AR-V7 target gene within this data set (Figure 8b). Pathway enrichment analysis showed a broader transcriptional signature than our ER TRACER, including significant downregulation of androgen response genes, as well as MYC target genes, fatty acid and cholesterol metabolism, and DNA repair genes (Figure 8c). Overall, we demonstrated that we could achieve targeted transcriptional repression of ER and AR/AR-V7 by developing TRACER heterobifunctional compounds that link an MBD2 recruiter to ER and AR ligand-binding domain modulators, thereby durably suppressing ER and AR transcriptional activity.

**Figure 7:**
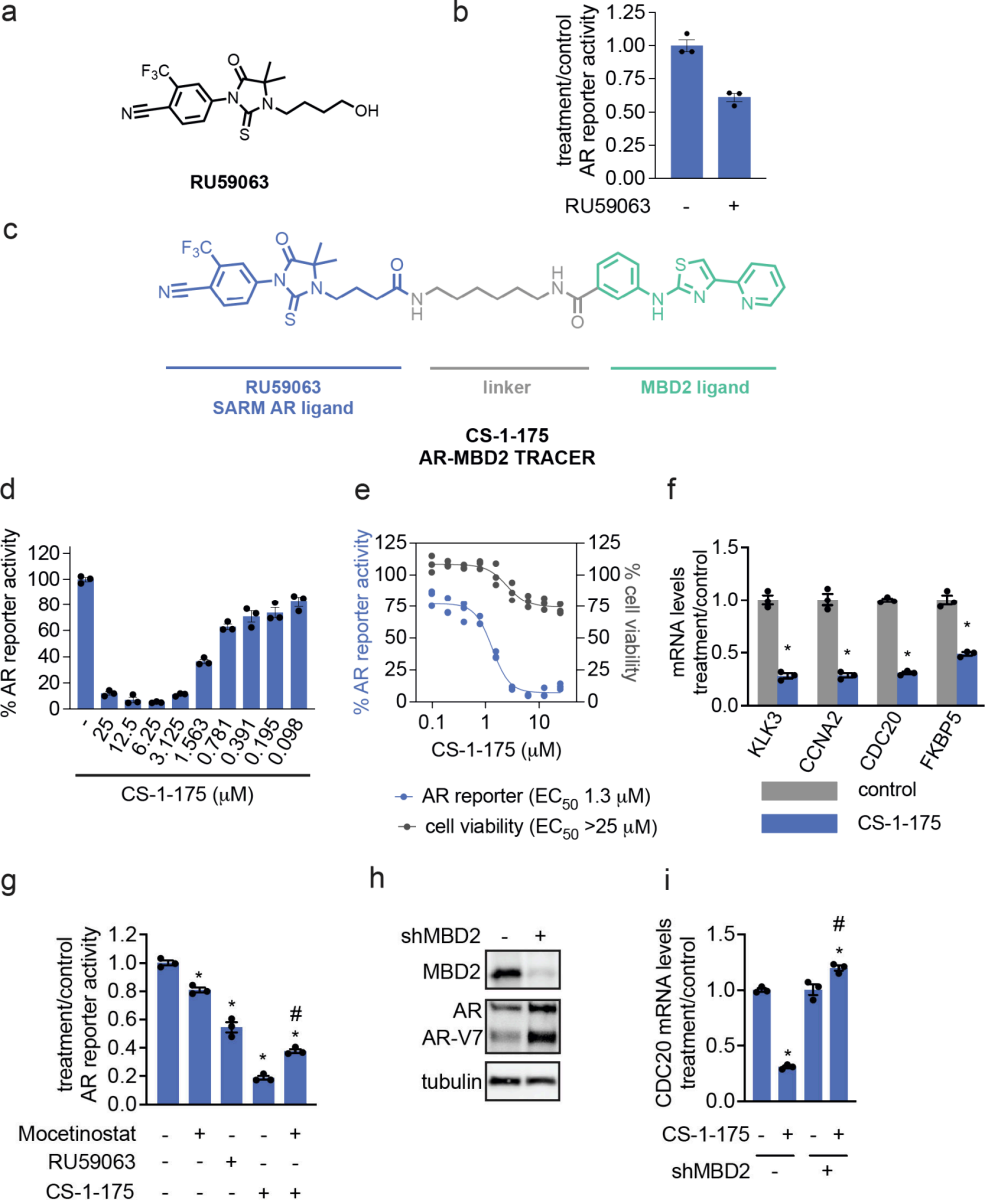
AR TRACER. (a) Structure of AR-targeting SARM RU59063. (b) AR luciferase reporter activity from RU59063 treatment in 22Rv1 androgen-independent prostate cancer cells. 22Rv1 cells were treated with DMSO vehicle or RU59063 (6.25 μM) for 24 h, after which AR transcriptional reporter activity was assessed. (c) Structure of AR TRACER CS-1-175. (d, e) Dose-responsive inhibition of AR luciferase reporter activity with CS-1-175 treatment in 22Rv1 cells compared to DMSO vehicle-treated controls (d), with an EC_50_ of 1300 nM from 24 h treatment (e). Also shown is cell viability assessed by Cell TiterGlo (e). (f) Downregulation of AR target genes with CS-1-175 treatment (6.25 μM) in 22Rv1 cells for 24 h compared to DMSO vehicle-treated controls. (g) CS-1-175-mediated inhibition of AR luciferase reporter activity in 22Rv1 cells is significantly attenuated upon pretreatment with HDAC inhibitor Mocetinostat. 22Rv1 cells were pretreated with DMSO, RU59063 (6.25 μM), or Mocetinostat (0.38 μM) for 1 h prior to treatment of cells with DMSO vehicle or CS-1-175 for 24 h. AR transcriptional reporter activity was subsequently assessed. (h) MBD2 knockdown in 22Rv1 cells. MBD2, AR, AR-V7, and loading control tubulin levels in Control versus shMBD2 cells. **(i)** RT-PCR of AR target gene CDC20. Control and shMBD2 22Rv1 cells were treated with DMSO vehicle or CS-1-175 (6.25 μM) for 24 h, and CDC20 **(i)** levels were assessed by RT-PCR. Data in (b, d–i) are from *n* = 3 biologically independent replicates per group. The blot shown in (h) is representative. Data shown in (b, d–g, i) are individual replicate values and the average (e) or average ± SEM (b, d, f, g, i). Significance is expressed as *p < 0.05 compared to vehicle-treated controls in (f, g, i) and #*p* < 0.05 compared to CS-1-175-treated or Control CS-1-175-treated groups in (g, i).

**Figure 8:**
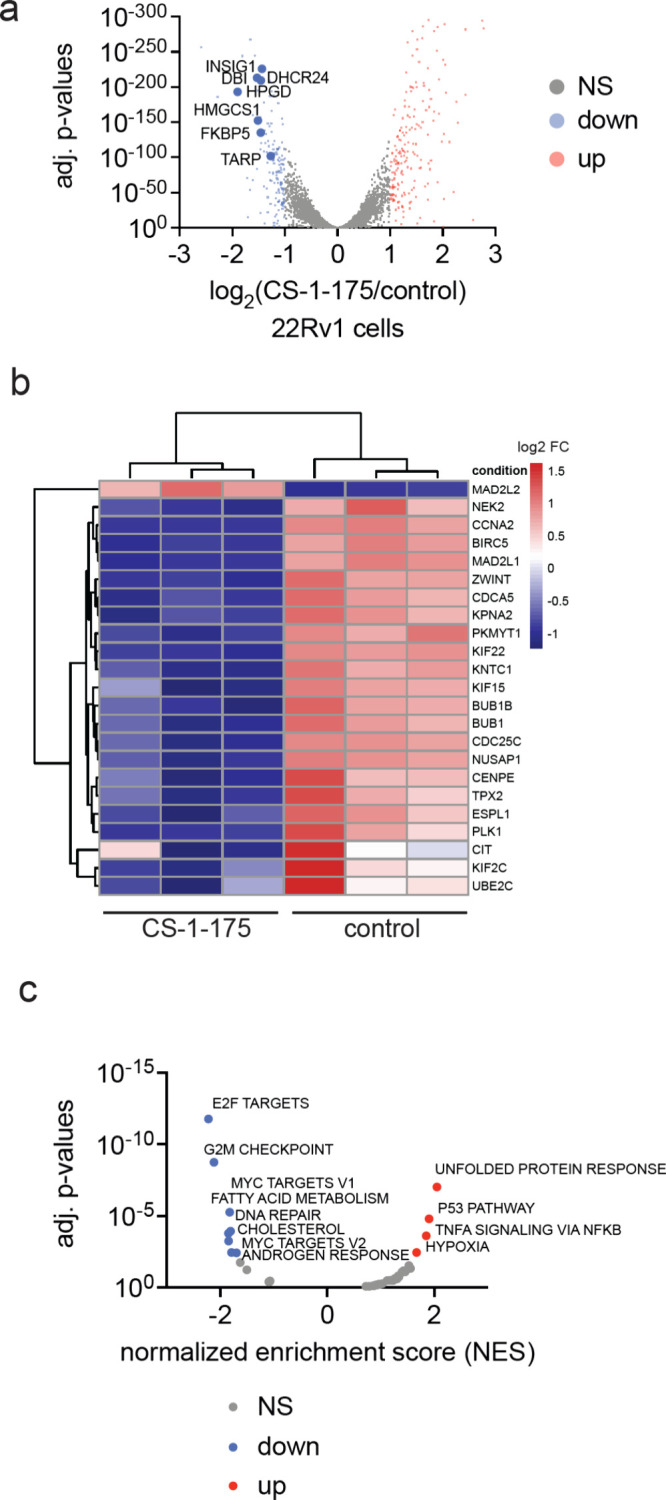
Transcriptomic analysis of AR TRACER CS-1-175. (a–c) 22Rv1 cells were treated with DMSO vehicle or CS-1-175 (12.5 μM) for 24 h, after which mRNA was extracted and subjected to RNA-seq Significantly altered genes were analyzed (a), heatmap analysis of previously identified AR-V7 target genes (b), and hallmark gene set enrichment analysis was performed to identify compound-mediated alterations in biological pathways (c). Data are from *n* = 3 biologically independent replicates per group and are available in Table S5.

## Discussion

The TRACER platform described here introduces a fundamentally new paradigm for pharmacologically targeting transcription factors—an enduring frontier in drug discovery. By co-opting endogenous transcriptional corepressor complexes and tethering them to disease-relevant transcription factor binding sites, TRACERs have the potential to overcome many of the liabilities that have hindered traditional small molecules, degraders, and even emerging modalities in this space. The demonstration that locus-specific repression can be achieved with small molecules in cancer cells establishes proof-of-concept for a modality that is modular, generalizable, and uniquely suited to target proteins long considered undruggable.

Several aspects of this study underscore the strength of this approach. First, TRACERs achieve durable and selective suppression of transcriptional programs, in contrast to the transient and often incomplete inhibition observed with antagonists or degraders of transcription factors with rapid turnover. Second, our results validate the on-target activity of TRACERs through multiple orthogonal approaches, including rescue with MBD2 knockdown, attenuation by HDAC inhibitors, and transcriptomic profiling demonstrating selective repression of bona fide target genes. Third, TRACERs can address clinically important resistance mechanisms. For example, while androgen receptor truncation variants such as AR-V7 evade ligand-directed antagonists and degraders, AR-TRACERs effectively silence both AR and AR-V7 transcriptional activity at their shared loci.

Interestingly, we observe that multiple ER and AR TRACERs bearing chemically diverse linkers, including alkyl, polyethylene glycol, and more rigid architectures, retain potent inhibitory activity. This tolerance to linker variation likely reflects the structural and functional properties of the recruited NuRD complex, which is a large, multisubunit assembly capable of engaging chromatin over extended spatial scales. Rather than requiring a highly constrained ternary geometry, TRACER activity may depend primarily on productive recruitment of NuRD to the target locus, allowing sufficient flexibility in linker composition and length to accommodate effective complex formation and histone deacetylation. This inherent plasticity may represent a key advantage of the TRACER modality relative to more geometrically constrained induced-proximity systems.

At the same time, several caveats merit consideration. The MBD2 ligand we use in this study is an early stage tool molecule and likely requires substantial medicinal chemistry efforts to optimize potency and likely its overall selectivity to be useful for broader applications and further drug discovery endeavors. Likely owing to the suboptimal nature of the ligand, our efforts to demonstrate target engagement at the proteome level using photoaffinity-based chemoproteomics have been unsuccessful so far. Future focus will also be on establishing further evidence of ternary complex formation mediated by our TRACERs between the transcription factor and MBD2 and NuRD complex, and also showing structural underpinning of ternary complex formation. Our proof-of-concept studies have thus far been confined to in vitro models, and the pharmacokinetic, pharmacodynamic, and safety properties of TRACERs in vivo remain to be established. Because TRACERs hijack endogenous corepressor complexes, differential expression or activity of these complexes across tissues may affect the efficacy and selectivity of these complexes. Furthermore, while our transcriptomic analyses suggest locus-selective repression, comprehensive genome-wide profiling will be needed to fully define off-target effects and to ensure that broad epigenetic remodeling does not undermine the therapeutic window. The chemical optimization of recruiter ligands, linker architectures, and transcription factor-binding ligands will also be critical to maximize potency, selectivity, and drug-like properties. Additionally, TRACERs still require a ligand that binds to the transcription factor target of interest, and ligand discovery against transcription factors remains challenging.

Looking ahead, TRACERs open several exciting avenues. The modular nature of this platform suggests it can be extended to a wide range of transcription factors beyond ER and AR, many of which remain untreatable yet are central to cancer and other diseases. Systematic exploration of alternative repressor complexes—such as NCoR/SMRT, SIN3A, or Polycomb family members—could expand the toolkit for tuning repression strength, durability, and context-specificity. From a translational standpoint, TRACERs may be particularly impactful in malignancies driven by transcription factors for which no targeted therapies exist, such as MYC, CTNNB1, NRF2, and YAP/TAZ.^9,11,31–33^ Beyond oncology, the ability to selectively reprogram gene expression programs raises the possibility of using TRACERs in inflammatory disorders, autoimmune diseases, and even rare genetic conditions in which pathological gene activation underlies disease.

In summary, TRACERs establish a new modality for silencing transcriptional drivers of disease by coupling the precision of small molecules with the power of epigenetic reprogramming. By addressing long-standing challenges in drugging transcription factors, this work lays the foundation for a versatile and expandable therapeutic strategy with broad implications across human disease.

## Supplementary Material













## Notes

Supporting Table Legends, Supporting Figures S1, S2, S3, S4, S5, S6, S7, S8, S9, S10, S11, and S12. Supporting Methods. Synthetic Methods and Characterization (PDF)
Table S1 (XLSX)
Table S2 (XLSX)
Table S3 (XLSX)
Table S4 (XLSX)
Table S6 (XLSX)
