# Supplementary material for: Targeted Transcriptional Repression by Induced Proximity
Source: ACS Cent Sci. 2026 Jun 11;12(7):1008–20. doi: 10.1021/acscentsci.5c02277 (PMC13397437; doi:10.1021/acscentsci.5c02277)

Supporting Information

Targeted Transcriptional Repression by Induced Proximity

Christian E. Stieger[1,2,3,4] , Xinru Chen[1,2,3], Christina C. Kuismi[1,2,3], Dustin Dovala[4,5], Daniel Fuller[4,5], Andreas O. Frank[4,5], Mikias Woldegiorgis[4,5], Megan Bruce-Smythe[4,5], Fabian Wu[4,5], Nicolas Pizzato [4,5], Jeffrey McKenna[4,5], Cory Johannessen[4,5], Barna D. Fodor[4,5], Markus Schirle[4,5], and Daniel K. Nomura [1,2,3,4] *

[1] Departments of Chemistry and Molecular and Cell Biology. University of California, Berkeley, Berkeley, CA 94720 USA

[2] Innovative Genomics Institute, Berkeley, CA 94720 USA

[3] Molecular Therapeutics Initiative, Berkeley, CA 94720 USA

[4] Novartis-Berkeley Translational Chemical Biology Institute, Berkeley, CA 94720 USA

[5] Novartis BioMedical Research, Emeryville, CA USA; Cambridge, MA USA; Basel, Switzerland

* Correspondence to cestieger@berkeley.edu and dnomura@berkeley.edu

Supporting Table Legends

Table S1. Proteomic profiling of CS-1-103. Proteomic analysis of T47D cells treated with DMSO vehicle or CS-1-103 (12.5 μM) for 24 h. Data are from n=3 biologically independent replicates per group.

Table S2. RNA sequencing of CS-1-103. RNAseq transcriptomic data of T47D cells co-treated with 1 nM E2 and DMSO vehicle or CS-1-103 (1.56 μM) for 24 h. Data are from n=3 biologically independent replicates per group.

Table S3. RNA sequencing of CS-1-103 in MBD2 knockdown cells. RNAseq transcriptomic data of T47D shMBD2 cells co-treated with 1 nM E2 and DMSO vehicle or CS-1-103 (1.56 μM) for 24 h. Data are from n=3 biologically independent replicates per group.

Table S4. ATTACseq of CS-1-103. ATTAC-seq data from T47D cells treated with DMSO vehicle or CS-1-103 treatment (1.56 μM) with 1 nM E2 over 24 h treatment time.

Table S5. RNA sequencing of CS-1-175. RNAseq transcriptomic data of 22Rv1 treated with DMSO vehicle or CS-1-175 (12.5 μM) for 24 h, after which mRNA was extracted and subjected to RNAseq. Data are from n=3 biologically independent replicates per group.

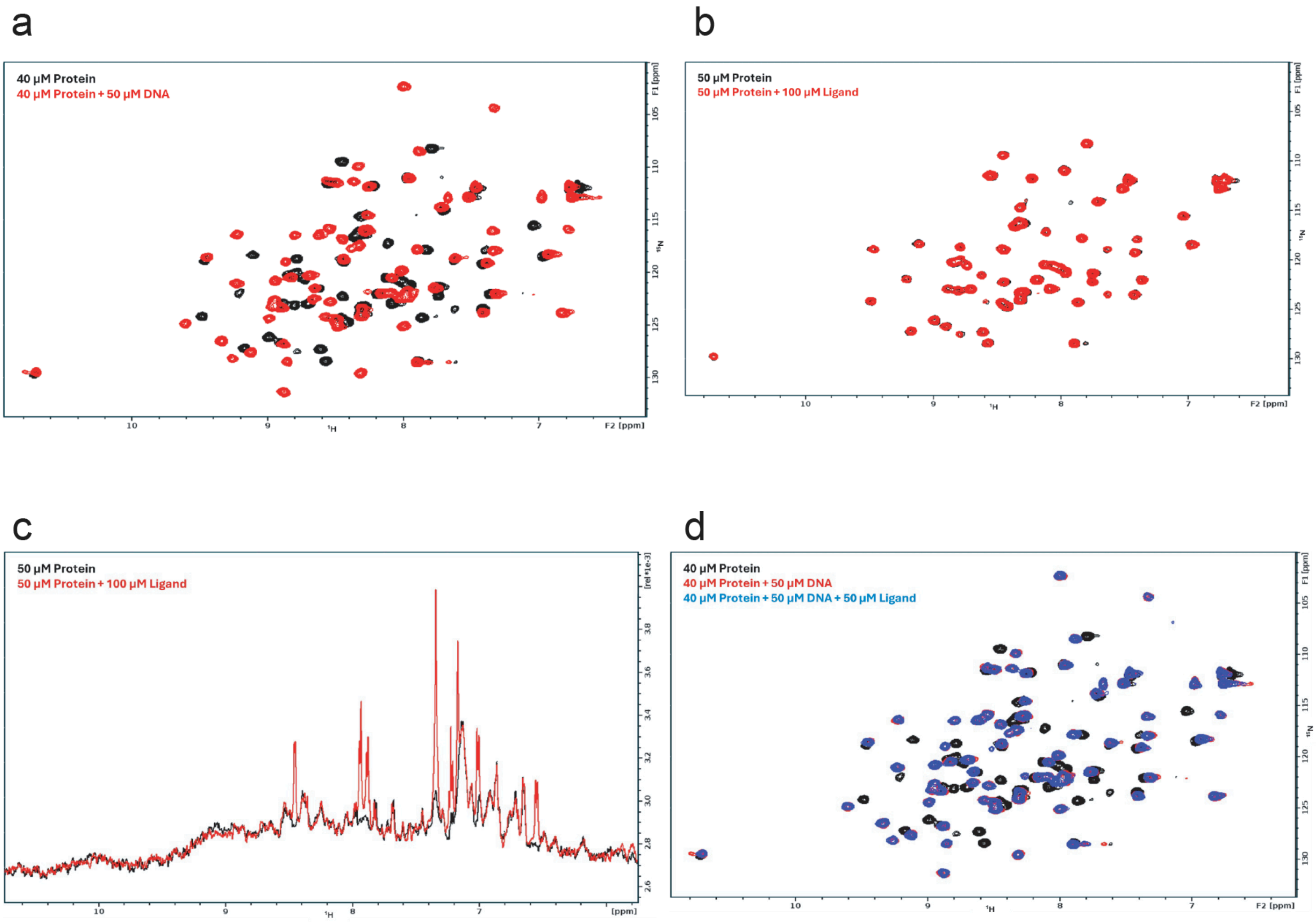

Figure S1. NMR spectroscopy analysis of ligand binding with methyl binding domain of MBD2.(a) Overlay of $^{1}H,^{15}N$-HMQC spectra of 40 μM MBD2(143-220) (black) and 40 μM MBD2 + 50 μM mCpG-DNA (red). Strong chemical shift perturbations were induced by the slight excess of nucleic acid, indicating that the protein is properly folded into its binding-competent structure and that it interacts with the added DNA. **(b)** Overlay of $^{1}H,^{15}N$-HMQC spectra of 50 μM MBD2(143-220) (black) and 50 μM MBD2(143-220) + 100 μM KC-007 (red). None of the protein peaks were perturbed upon addition of a 2:1 excess of the small molecule. The ^{1}H-1D spectra recorded on the same samples (see **(c)**) proved that the ligand is dissolved at the nominal concentration of 100 μM under the conditions employed in the experiments. The combined results from both datasets indicate that KCC-07 does not interact with MBD2(143-220) at the protein and ligand concentrations used. **(c)** Overlay of ^{1}H-1D spectra of 50 μM MBD2(143-220) (black) and 50 μM MBD2(143-220) + 100 μM KCC-07 (red). The section of the spectra shown includes the chemical shift range typical for aromatic and amide protons. Peaks from the small molecule are clearly visible on top of the protein envelope peaks, indicating that the compound is well dissolved in the aqueous solution under the chosen conditions. **(d)** Overlay of $^{1}H,^{15}N$-HMQC spectra of 40 μM MBD2 (black), 40 μM MBD2(143-220) + 50 μM mCpG-DNA (red) and 40 μM MBD2(143-220) + 50 μM mCpG-DNA + 50 μM KCC-07 (blue). While the addition of DNA induced strong chemical shift perturbations of MBD2 peaks, there are no further shifts upon addition of KCC-07. This observation indicates that the small molecule does not interact with DNA-bound MBD2(143-220).

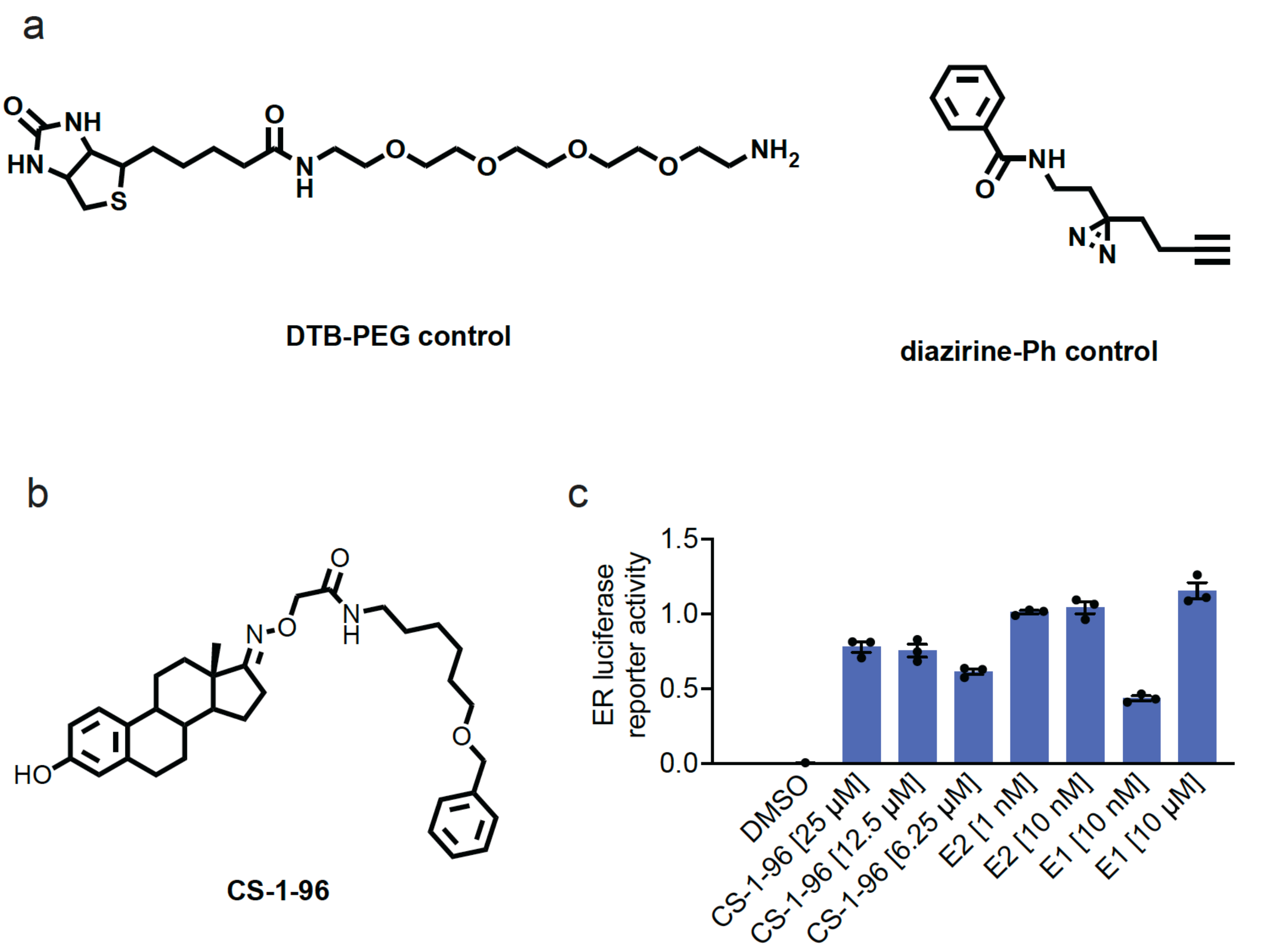

Figure S2. Negative control probes. (a) Structures of negative-control desthiobiotin and alkyne-functionalized diazirine probes. **(b)** Structure of negative-control ER TRACER probe. **(c)** ER luciferase transcriptional reporter assay in T47D cells grown in charcoal-stripped media, where ER agonists were added back, and luciferase reporter activity was assessed. Shown in **(c)** are individual replicate values and average ± sem, from n=3 biologically independent replicates per group.

a

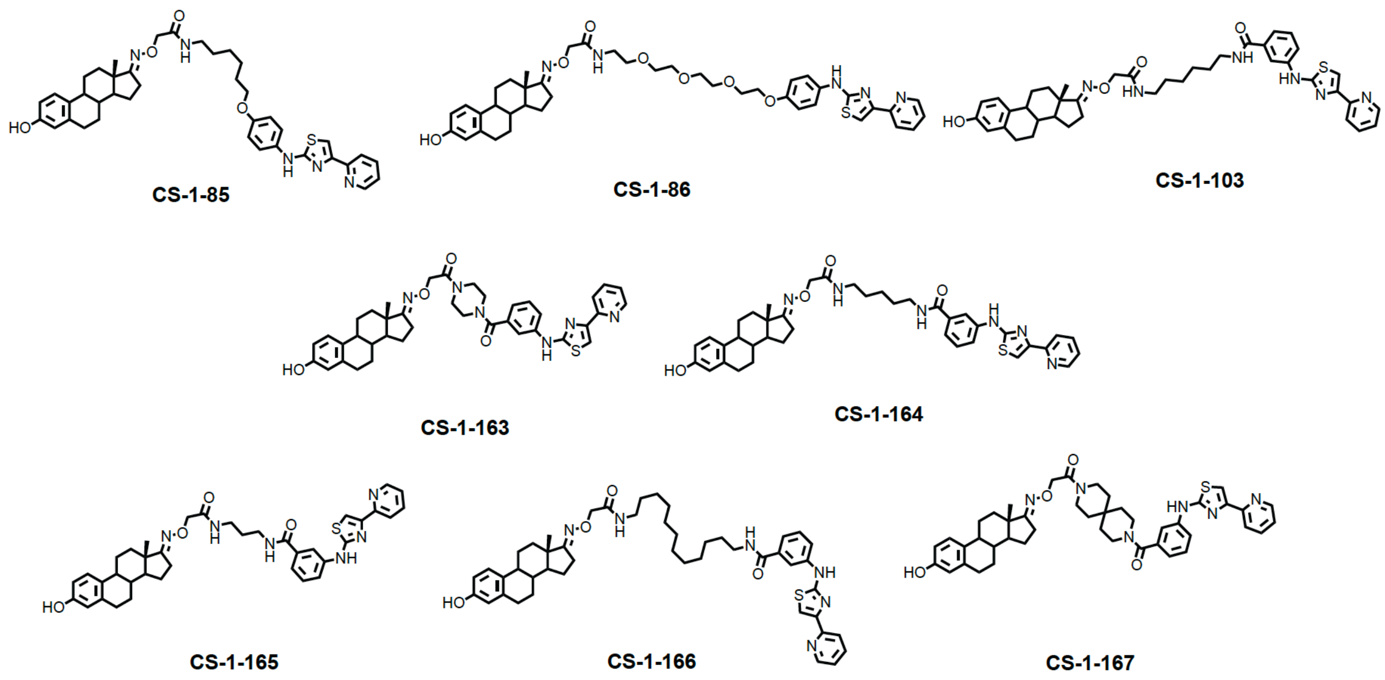

b

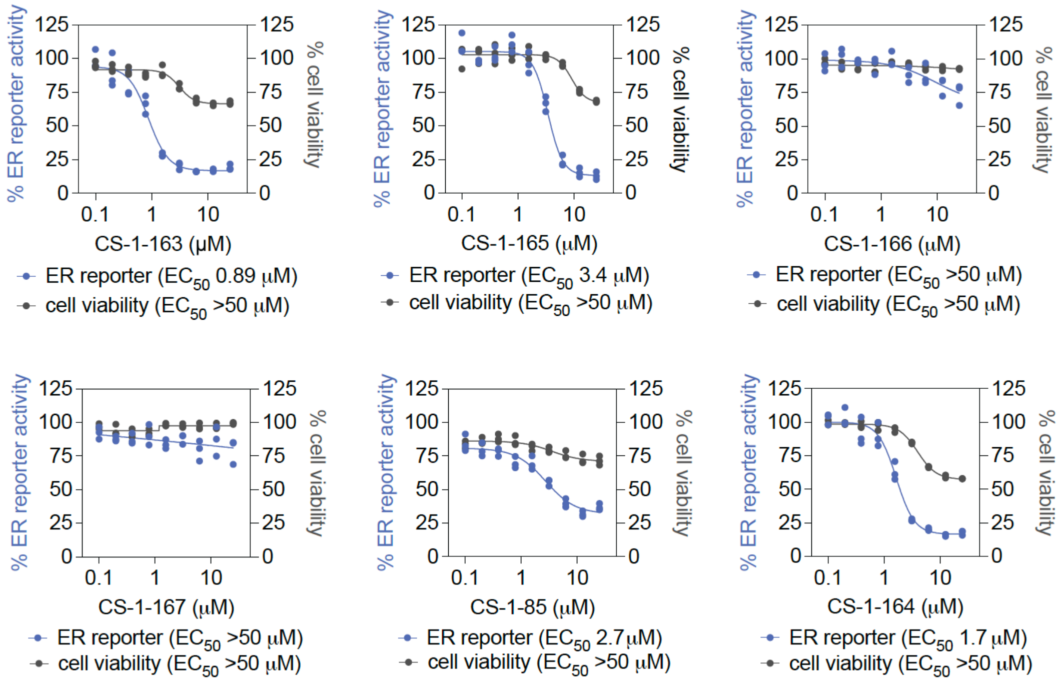

Figure S3. Characterization of ER TRACERs. (a) Structure of ER TRACER CS-1-86. **(b)** Dose-responsive inhibition of ER luciferase transcriptional reporter activity and cell viability (assessed by Cell TiterGlo) in T47D cells upon treatment with DMSO vehicle or TRACER for 24 h, showing EC50 values. Shown in **(b)** are individual replicate values and the average from n=3 biologically independent replicates per group.

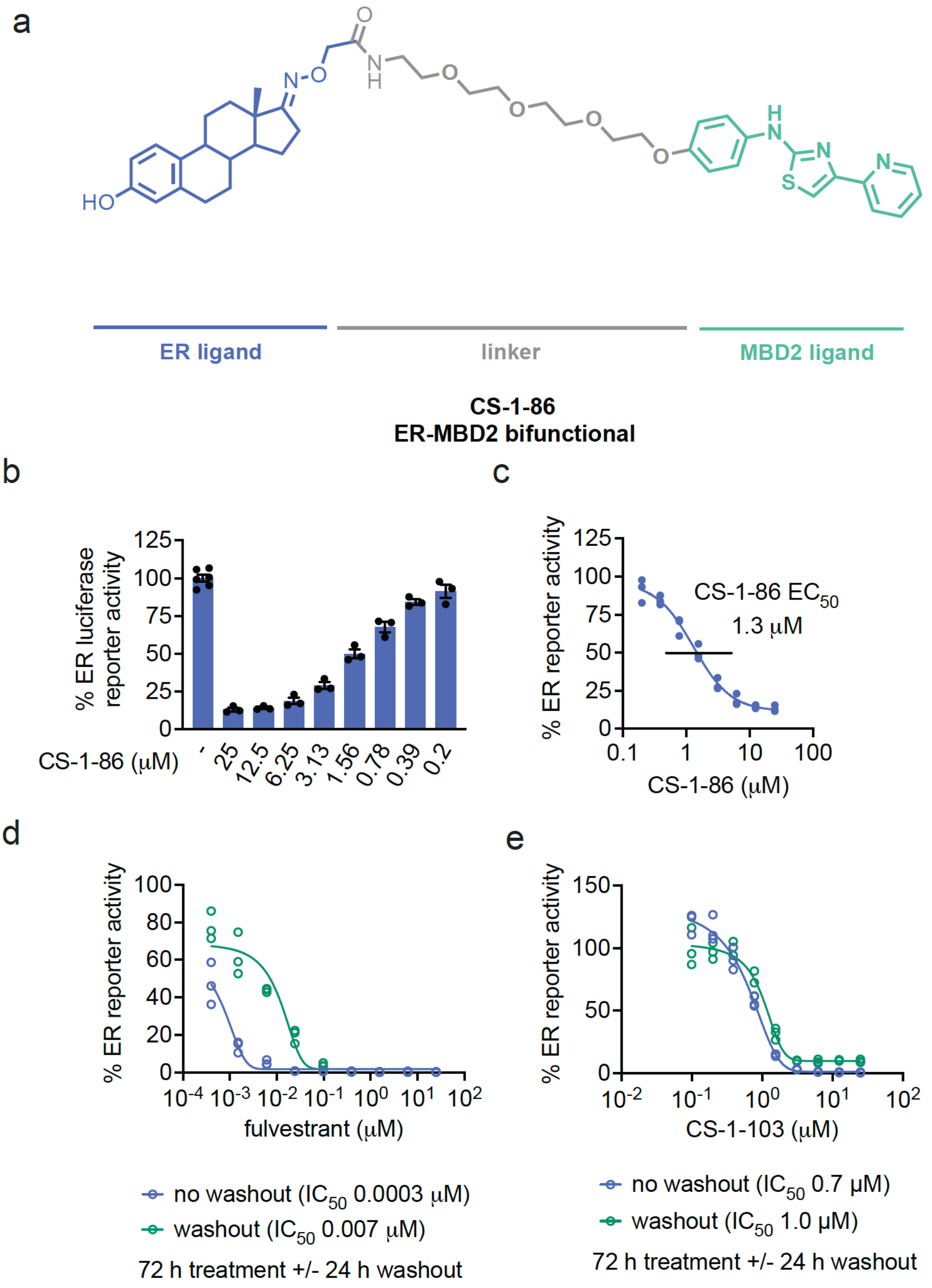

Figure S4. Characterization of CS-1-86, fulvestrant, and CS-1-103. (a) Structure of ER TRACER CS-1-86. **(b,c)** Dose-responsive inhibition of ER luciferase transcriptional reporter activity in T47D cells upon treatment with DMSO vehicle or TRACER CS-1-86 for 24 h, showing an EC_{50} of 1.3 μM. **(d,e)** Dose-response of ER luciferase transcriptional reporter inhibition in T47D cells from treatment of cells with DMSO vehicle, fulvestrant **(d)** or CS-1-103 **(e)** for 72 h or 72 h and then 24 hours of compound washout. Data in **(b-e)** are from n=3 biologically independent replicates per group. Shown in **(b-e)** are individual replicate values and average ± sem **(b)** or the average **(c,d,e)**.

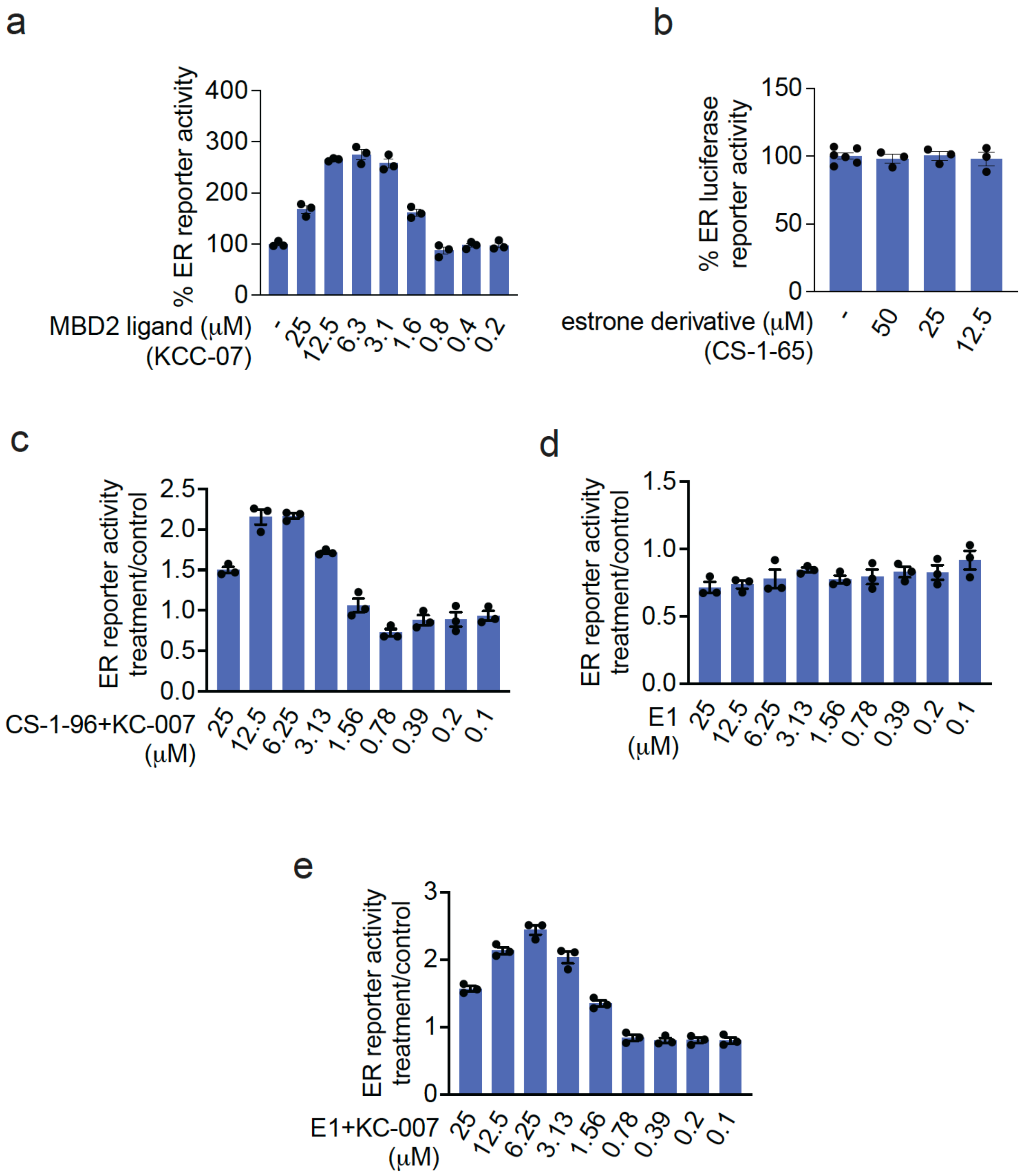

Figure S5. Testing individual components of TRACERs alone or in combination. (a-e) T47D ER luciferase reporter activity after treatment with DMSO vehicle, KCC-07, CS-1-65, CS-1-96 and KC-007, E1, or E1 and KC-007 for 24 h. Shown are individual replicate values and average ± sem from n=3 biologically independent replicates per group.

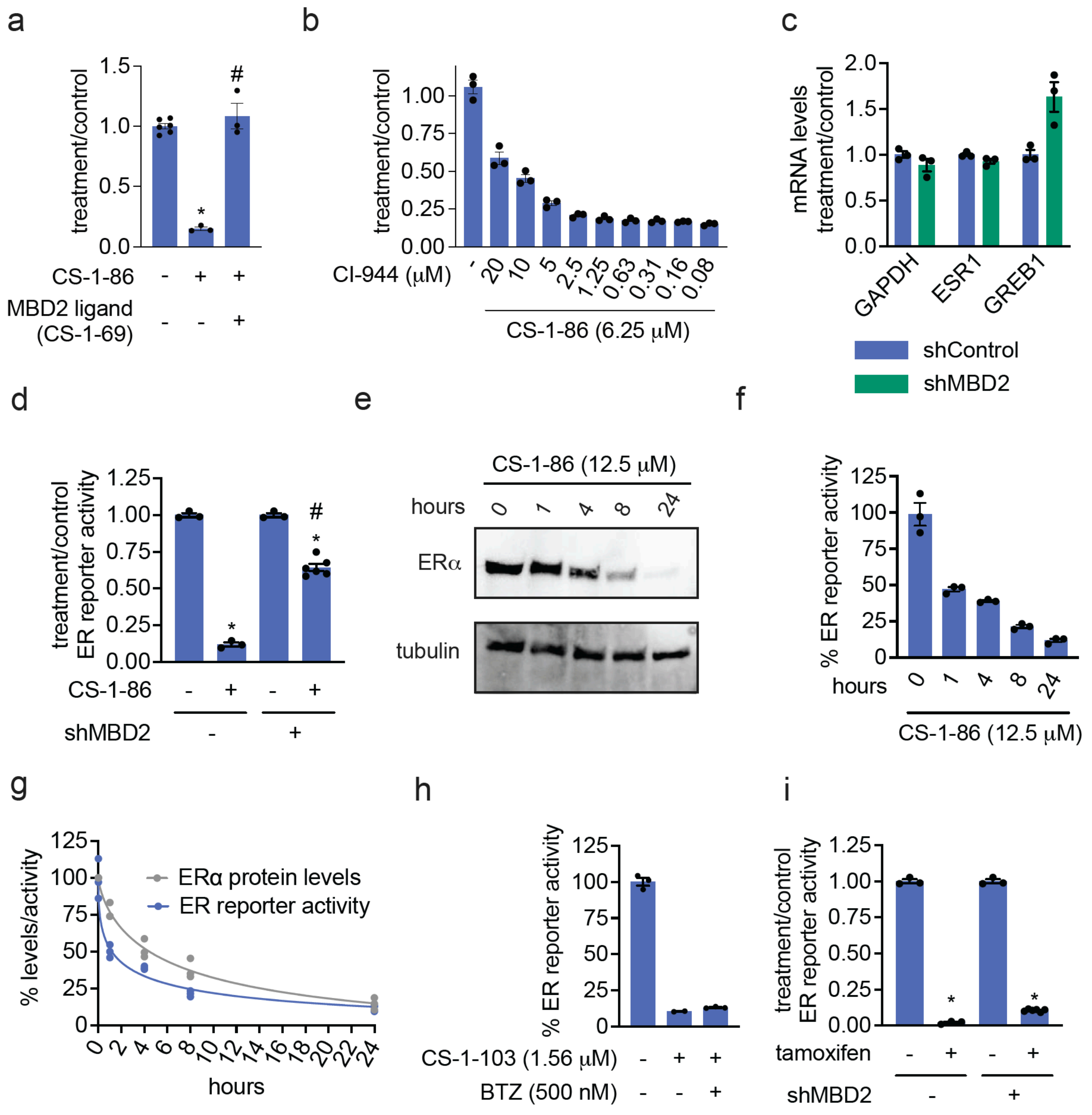

Figure S6. Further characterization of ER TRACERs. (a,b) Attenuation of CS-1-86-mediated ER luciferase reporter inhibition in T47D cells with CS-1-69 or HDAC inhibitor CI-944. ER luciferase reporter T47D cells were co-treated with DMSO vehicle, CS-1-69 (50 μM) **(a)**, or pre-treated with CI-944 **(b)** for 1h prior to treatment of cells with DMSO vehicle or CS-1-86 (12.5 μM or 6.25, respectively) for 24 h, after which ER luciferase transcriptional activity was read out. **(c)** GAPDH, ESR1, and GREB1 mRNA levels in shControl or shMBD2 T47D cells. **(d)** Attenuation of CS-1-86-mediated inhibition of T47D ER luciferase reporter activity upon MBD2 knockdown. T47D shControl versus shMBD2 cells were treated with DMSO vehicle or CS-1-86 (12.5 μM) for 24 h, after which ER luciferase transcriptional activity was read out. **(e,f)** ER protein levels from CS-1-86 treatment. T47D cells were treated with CS-1-86 (12.5 μM), and ER and loading control tubulin levels were assessed by SDS/PAGE and Western blotting **(e)** and quantified in **(f).** **(g)** ER transcriptional reporter activity and ER protein levels from CS-1-86 treatment in a time-course study.

T47D cells were treated with CS-1-86 (12.5 μM) for the designated time-points, and ER luciferase reporter activity and ER protein levels were assessed and plotted. **(h)** CS-1-103-mediated ER transcriptional reporter activity is not attenuated by proteasome inhibitor pre-treatment. ER luciferase reporter T47D cells were pre-treated with DMSO vehicle or bortezomib (500 nM) for 1 h prior to treatment of cells with DMSO vehicle or CS-1-103 (1.56 μM) for 24 h, after which ER luciferase reporter activity was read out. **(i)** Tamoxifen-mediated inhibition of ER transcriptional reporter activity is not MBD2-dependent. T47D Control and shMBD2 cells were treated with DMSO vehicle or tamoxifen (10 μM) for 24 h, after which ER luciferase reporter activity was read out. Data in **(a-i)** are from n=3 biologically independent replicates per group. Blot in **(e)** is representative. Bar graphs and plots in **(a-d,g-i)** show individual replicate values, and bar graphs in **(a-d, d,f,h,i)** show average ± sem and the plot in **(g)** shows the average. Significance expressed as *p<0.05 compared to vehicle-treated controls in **(a,d,i)** and #p<0.05 compared to CS-1-86 treatment alone in **(a)** or CS-1-86 treatment in Control cells in **(d).**

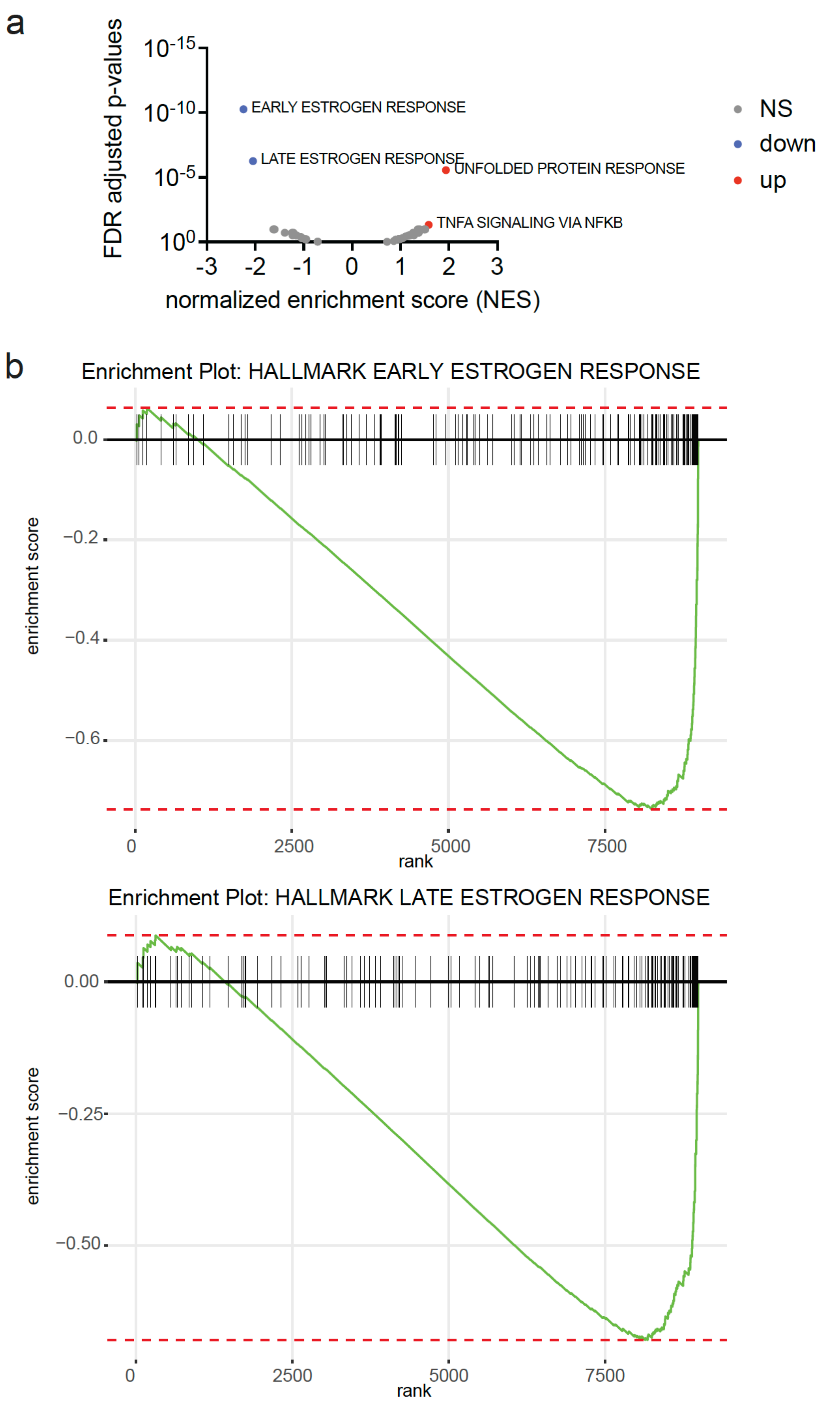

Figure S7. Gene enrichment analysis of ER TRACER RNAseq data. (a) fGSEA hallmark gene enrichment analysis of ER TRACER. **(b)** Enrichment plot of preset hallmark gene sets of cells treated with ER-TRACER compared to control. Top ranked downregulated gene sets are shown. **(c)** Enrichment plot of ER-TRACER regulated genes on a representative ChEA 2022 ESR1 dataset.

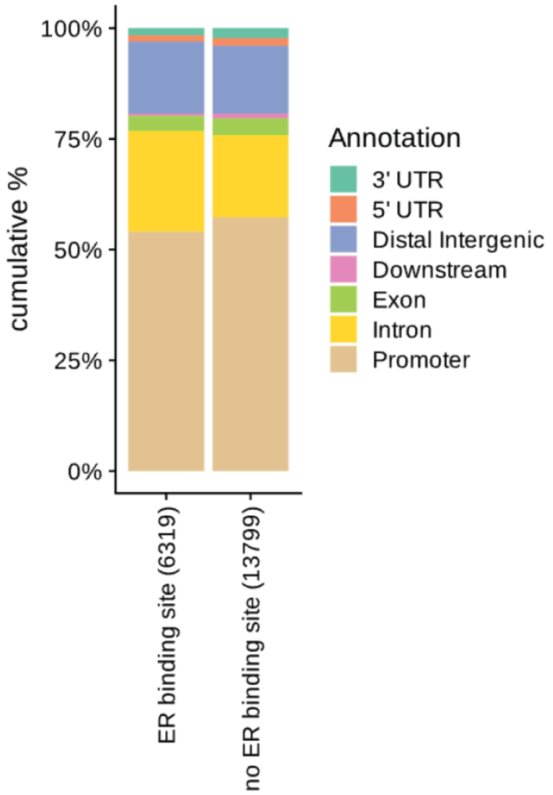

Figure S8. Genomic feature distributions of ATAC-seq peaks stratified by ER occupancy (CUT&RUN). Genomic feature annotations were plotted in a cumulative bar graph to illustrate the distributions of genomic features of ATAC-seq peaks with or without ER binding sites.

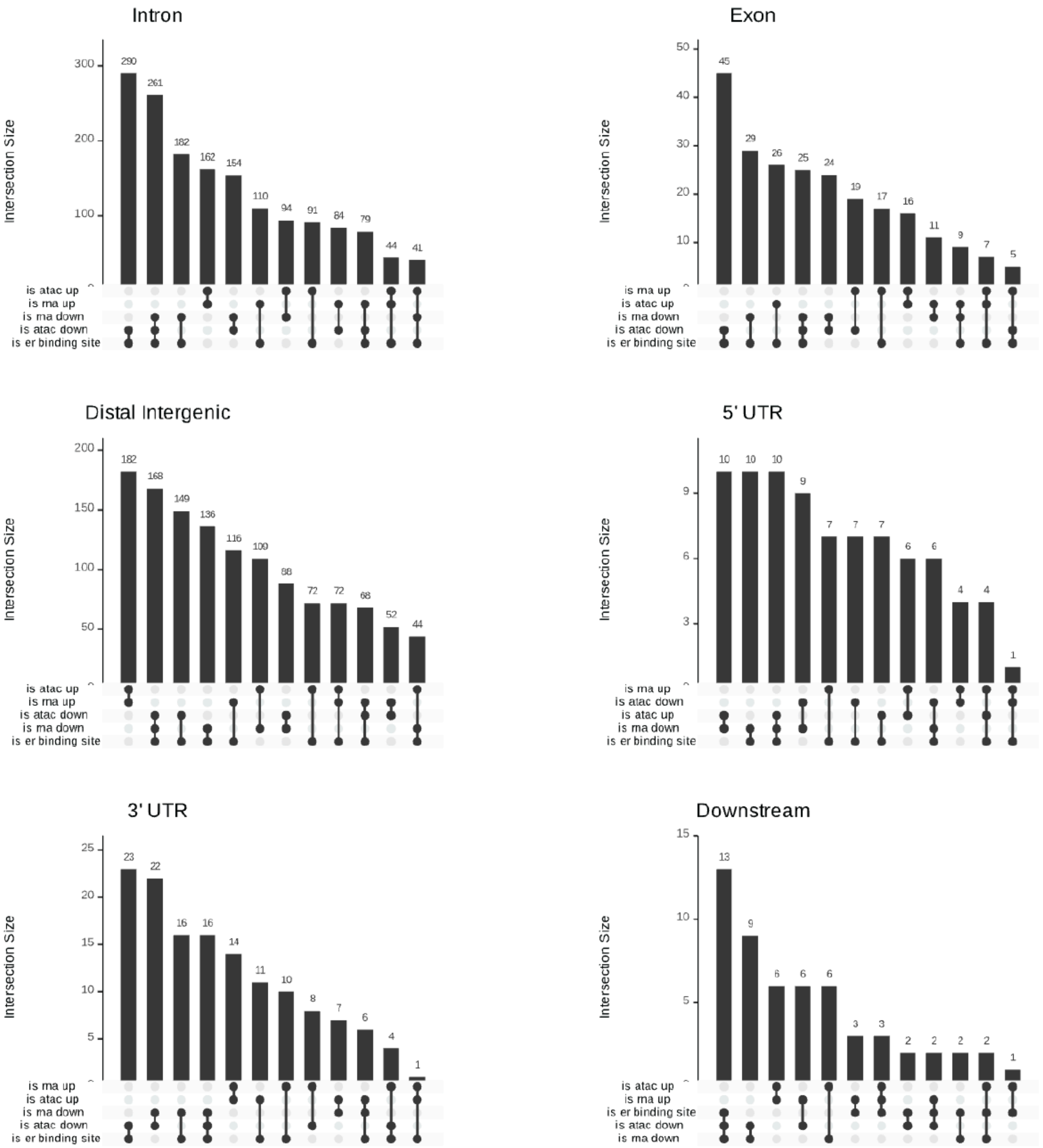

Figure S9. Genomic distribution of coordinated chromatin and transcriptional changes. UpSet plots illustrating the intersection of differential accessibility (ATAC-seq), differential expression (RNA-seq), and ER occupancy (CUT&RUN), stratified by genomic annotation. Changes in RNA expression were assigned to regulatory elements based on the proximity of the nearest transcription start site (TSS). Categories denote the directionality of change (Up/Down) for both accessibility and expression.

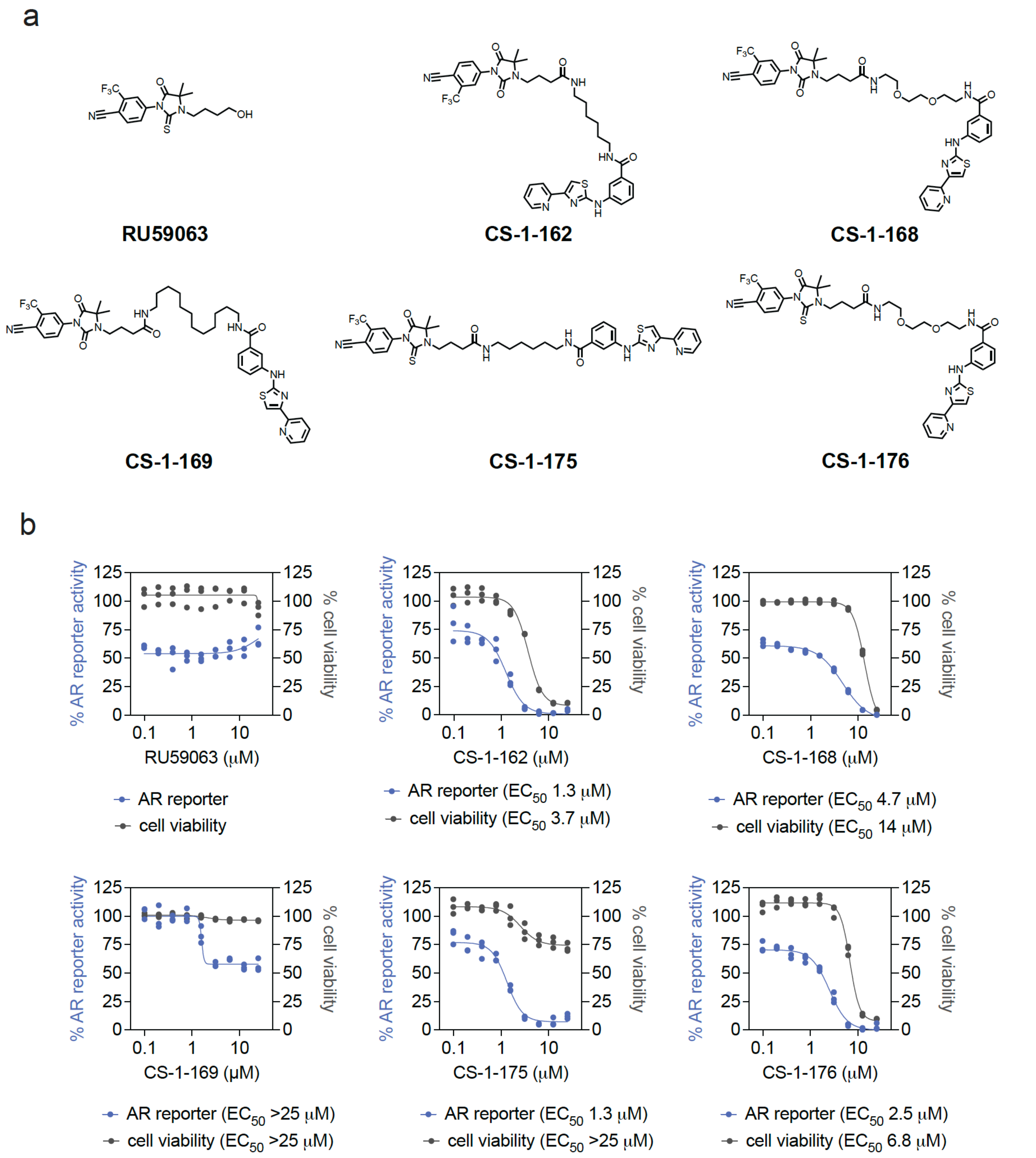

Figure S10. Characterization of AR TRACERs. (a) Structure of SARM RU59063 and MBD2-based AR TRACERs with varying linkers. **(b)** AR luciferase transcriptional reporter activity and cell viability in 22Rv1 cells. AR luciferase reporter 22Rv1 cells were treated with RU59063, CS-1-162, CS-1-168, CS-1-169, CS-1-175, or CS-1-176 for 24 h after which AR luciferase reporter activity was read out and cell viability was also assessed by Cell TiterGlo. Shown below each plot are EC_{50} values for both AR transcriptional reporter activity and cell viability. Data in **(b)** are from n=3 biologically independent replicates per group and shown are individual replicate values.

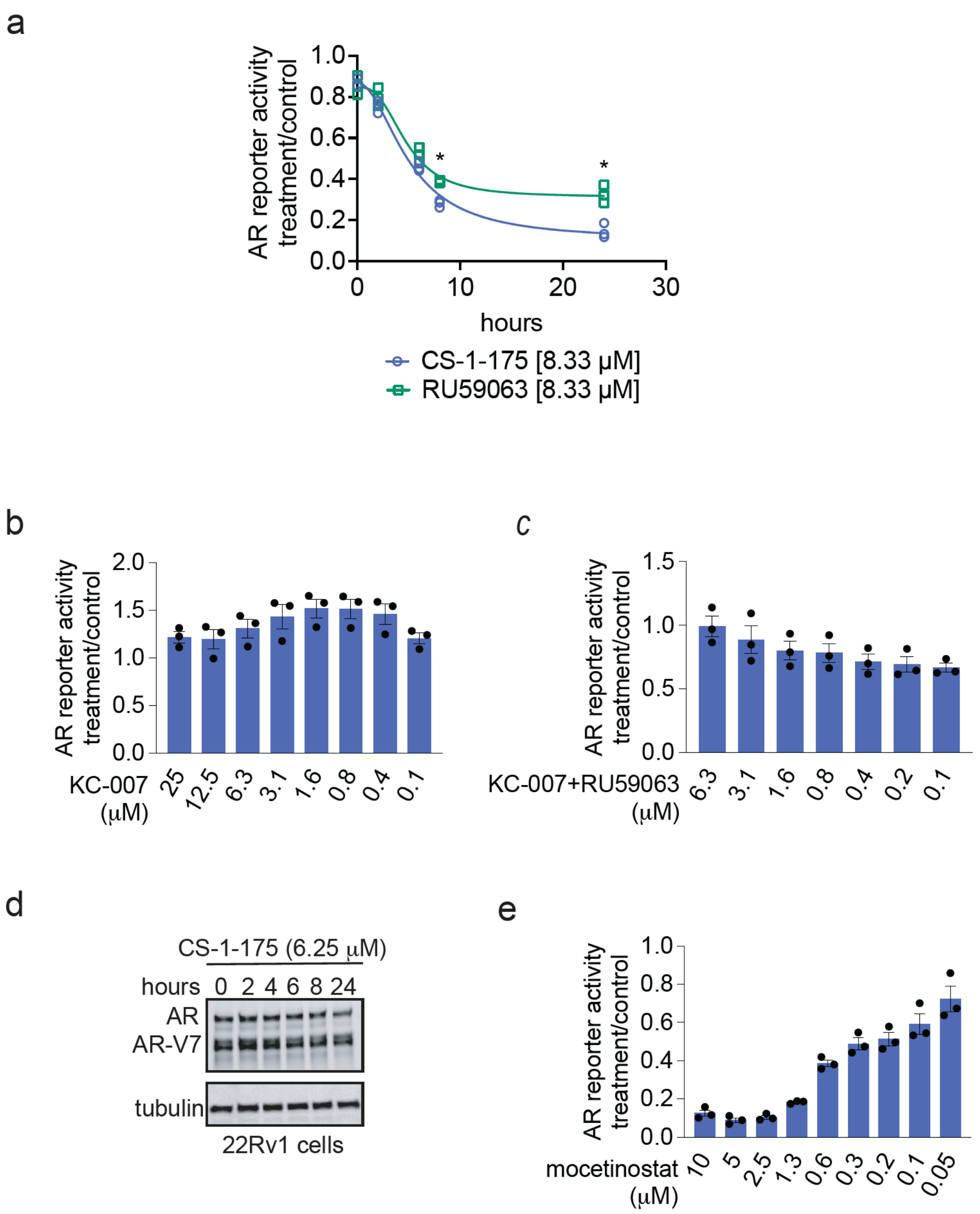

Figure S11. Characterization of AR TRACER and its individual components. (a) AR transcriptional reporter activity in 22Rv1 cells. 22Rv1 AR luciferase reporter cells were treated with DMSO vehicle, CS-1-175, or RU59063, and AR transcriptional activity was assessed. **(b-c)** AR transcriptional reporter activity in 22Rv1 cells treated with DMSO vehicle, KC-007 **(b)**, or KC-007 and RU59063 **(c)** for 24 h. **(d)** AR and AR-V7 protein levels in 22Rv1 cells. 22Rv1 cells were treated with DMSO vehicle or CS-1-175 for 24 h, after which AR and AR-V7 and loading control tubulin levels were assessed by SDS/PAGE and Western blotting. **(e)** AR transcriptional activity in 22Rv1 cells treated with DMSO vehicle or HDAC inhibitor mocetinostat for 24 h. Data in **(a-e)** are from n=3 biologically independent replicates per group. Plots and bar graphs show individual replicate values and the average **(a)** or average ± sem **(b,c,e)**. Blot in **(d)** is representative.

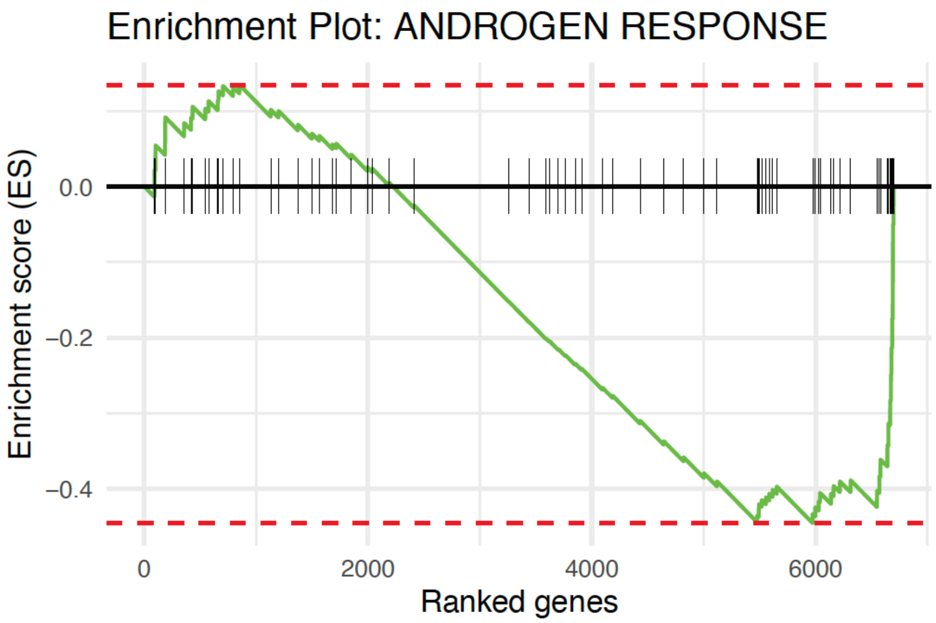

Figure S12. Transcriptomic analysis of AR TRACER CS-1-175. 22Rv1 cells were treated with DMSO vehicle or CS-1-175 (12.5 μM) for 24 h, after which mRNA was extracted and subjected to RNA-seq. Shown is an enrichment plot of hallmark androgen response genes from these data.

Supporting Methods

Protein Production

Methyl Binding Domain of MBD2: The methyl binding domain of MBD2 (residues 143-220) was codon optimized and synthesized in line with an N-terminal His-SUMO tag in a pET-based expression plasmid. The plasmid was used to transform BL21(DE3) *E. coli* cells, and a single colony was used to inoculate an overnight culture in LB media. This overnight culture was then used to inoculate 2 x 1 L cultures in terrific broth (supplemented with 50 mM sodium phosphate pH 8.0 and 50 μg/mL kanamycin), which were grown at 37 °C while shaking at 225 rpm in 2.8 L Fernbach flasks. These cultures were allowed to grow until the OD_{600} reached 0.8-1.0, at which point the temperature was reduced to 19 °C and the cultures were supplemented with 0.5 mM isopropyl-D-1-thiogalactopyranoside (IPTG) and allowed to grow overnight. The following day, cultures were harvested via centrifugation at 6,000 x *g* for 10 minutes, followed by resuspension in a 4:1 (v/w) ratio in Lysis Buffer (50 mM Tris pH 8.0, 400 mM NaCl, 1 mM TCEP, 5% glycerol). Cell lysis was performed via sonication on ice, and was then clarified via centrifugation at 45,000 x *g* for 30 minutes. The supernatant was subjected to immobilized metal affinity chromatography (IMAC) by application to a column containing approximately 7.5 mL of Ni-NTA resin pre-equilibrated with Lysis Buffer. Following loading, the resin was washed with 5 column volumes (CV) of Lysis Buffer, followed by 5 CV of Lysis Buffer supplemented with 40 mM imidazole, followed by elution with 5 CV of Lysis Buffer supplemented with 500 mM imidazole. The eluate was treated with ULP1 (SUMO protease) and allowed to dialyze overnight in 3 L of Lysis Buffer overnight. Complete cleavage was observed the following day, and the protein was subjected to reverse IMAC by application to a column containing approximately 7.5 mL of Ni-NTA resin pre-equilibrated with Lysis Buffer. The flow-through was collected, concentrated, and applied to a size exclusion chromatography (SEC) column on an AKTA FPLC (Superdex 75 26/60 with a flow rate of 4 mL/min) pre-equilibrated with SEC Buffer (25 mM HEPES pH 7.5, 150 mM NaCl, 1 mM TCEP, 5% glycerol). Pure fractions in the included volume were pooled, concentrated, and stored at -70 °C. For ^{15}N-labeled MBD2 methyl binding domain, the same construct and purification procedure was used; however, the cells were grown in M9 minimal media made with ^{15}N-labeled NH_4Cl (CIL NLM-467-25).

NuRD Core Complex: The NuRD Core Complex was purified similar to previously reported [1], by co-expression of three CMV-driven expression plasmids in Expi293F cells. These were His-FLAG-TEV-MTA1(162-354), TwinStrep-MBD2(145-411), and untagged HDAC1(1-482). Plasmids were transfected in a 1:1:1 ratio via Expifectamine using standard procedures. Cells were harvested 4 days post transfection and resuspended 10:1 (v/w) into Lysis Buffer (50 mM tris, pH 7.5, 100 mM potassium acetate, 10% glycerol, 0.3% Triton X100, 1 x cOmplete protease inhibitor) and lysed via sonication on ice. Cell lysate was clarified by centrifugation at 43,000 x *g* for 45 minutes. The supernatant was incubated in batch with 2 mL of anti-FLAG M2 affinity resin pre-equilibrated with Buffer A (50 mM tris, pH 7.5, 100 mM potassium acetate, 10% glycerol, 0.3% Triton X100, 1 x cOmplete protease inhibitor), allowed to bind for one hour, and then applied to an empty column. The resin was then washed with 25 CV of Buffer B (50 mM tris, pH 7.5, 100 mM potassium acetate, 5% glycerol). The resin was collected and incubated in batch with 40 mL of Buffer B containing 100 μg/mL RNase A for one hour, before application to another empty column and washing with 25 CV of Buffer C (25 mM tris, pH 7.5, 75 mM potassium acetate, 0.5 mM TCEP). The resin was collected and incubated in 4 mL of Buffer C supplemented with 25 μg/mL TEV protease while nutating at 4 °C overnight. The resin was

then applied to an empty column and the flow-through collected. The flow-through was concentrated and applied to a SEC column (Superose 6 10/30) pre-equilibrated with Buffer C with a flow rate of 0.5 mL/min. Peak fractions in the included volume were analyzed by ESI-LC/MS and anSEC. Those containing the NuRD complex were pooled, concentrated, and stored at -70 °C.

NMR spectroscopy

We prepared multiple NMR samples to test the functionality of our recombinant MBD2(143-220) protein and probe for interactions with the noted compound: our reference samples contained 40 µL of 40 µM or 50 µM MBD2, 25 mM Hepes pH 7.5, 150 mM NaCl, 10% D_2O, 11.1 µM DSS, 1% DMSO and 1 mM d-TCEP. Our test samples had the same composition but also contained 50 µM or 100 µM of the noted compound (at a 1% total concentration of DMSO) and/or 50 µM of mCpG-DNA. For all samples, we recorded ^{1}H-1D and ^{1}H,^{15}N-SOFAST-HMQC spectra at 298K using a Bruker Neo 600 MHz NMR spectrometer equipped with a 1.7 mm TCI cryo probe. A standard Bruker 1H-1D setup with excitation sculpting water suppression (pulse sequence *zgesgp*) was used to record the proton spectra. The Bruker *BB_2d_sofast_hmqc* pulse sequence using a 90º flip angle and heteronuclear *garp4* decoupling was applied to record the 2D spectra. The recycling delay was 1 s, the ^{1}J(NH) coupling constant was set at 90 Hz, and the number of scans (dummy scans) was 64 (32). The number of recorded points in the proton (nitrogen) dimension was 1k (128), the spectral width in the proton (nitrogen) dimension was 15 (36) ppm, and the center frequency in the proon (nitrogen) dimension was 4.67 (118) ppm. The proton excitation frequency was 8.5 ppm using an excitation bandwidth of 4.5 ppm. The duration of each ^{1}H,^{15}N-2D experiment was 150 minutes. 2k (256) points were processed in the direct (indirect) dimension while using Gaussian and squared sine window functions. Spectra were analyzed using Bruker Topspin 4.5.

ASMS Binding Assay

The ASMS binding assay was conducted on NuRD complex, MBD2, and HDAC1 (Cayman Chem., catalog #: 10009231) to distinguish binding to individual components compared to the complex. A control plate without targets was checked for compound aggregation and false positives due to poor solubility. To prepare assay ready plates, 180 nL of compound stock solution in 8 point dose response (10 mM top concentration, 1:3 dilution) was transferred using a Labcyte Echo into a 384-well Greiner low-volume plate. The assay buffer consists of 25 mM HEPES, pH 7.5, 100 mM NaCl, and 0.0005 % MNG-3. 15 µl of 500 nM protein was dispensed into each well of the assay-ready plates and 15 µl buffer only was added to the compound only control plate. All plates were incubated at room temperature for 1 hour prior to run. Plates were centrifuged and then loaded onto a UPLC auto sampler at 8 °C for ASMS analysis. The run was set up on an Agilent 1290 Infinity II UPLC 2D hybrid system, comprising two pumps, one diode array detector, and one Agilent 6545XT Q-ToF. To start the run, a 5 µL injection is made and sent through a 2.1 × 50 mm 5 µm 60Å PolyHydroxyEthyl A SEC column (PolyLC) using phosphate buffered saline solution (PBS) as an isocratic mobile phase. The column is maintained at 8 °C. The eluent containing protein and potential ligand binder is collected in a 50 µl sample loop and sent to the second column, which is a 2.1 × 50 mm C18 1.7 µm reversed BEH column (Waters) maintained at 60 °C. The mobile phase consists of (A) 0.1% formic acid with 10 mM ammonium formate and (B) 0.1% formic acid in 50:50 ACN/MeOH. Gradient A:B 98:2 hold for 0.8 min; ramp to 1:99 by 2.3 min, hold until 3.3 min; return to 98:2 by 3.5 min. The UHPLC eluent is diverted

to waste from 0 to 0.8. The SEC column is flushed with a 50% ACN solution from 0.33 to 1.6 minutes during the run, effectively removing residual small molecules. Subsequently, the column is re-equilibrated with PBS for 2 minutes. Data was analyzed using a workflow in Analytical Studio Pro by Virscidian (Version 14.2, Virscidian, Cary, NC).

Cell Culture

T47D wild-type (obtained from UC Berkeley Cell Culture Facility) and Estrogen Receptor Luciferase Reporter T47D Stable Cell Line (obtained from Signosis, SL-0002) was cultured in RPMI-1640 medium containing 10% (v/v) fetal bovine serum (FBS) and 1x glutamine. For hormone competition experiments, cells were grown in phenol-red-free RPMI supplemented with 10% charcoal-treated FBS for at least 72 h prior to treatment.
22Rv1wild-type and Androgen Receptor Luciferase Reporter 22Rv1 cells (both from UC Berkeley Cell Culture Facility) were cultured in RPMI-1640 medium containing 10% (v/v) fetal bovine serum (FBS) and 1× GlutaMAX.
HEK293T (UC Berkeley Cell Culture Facility) were cultured in DMEM containing 10% (v/v) fetal bovine serum (FBS).
All cell lines were maintained at 37°C and 5% CO_2.

Pulldown analysis using MBD2-Biotin-Probe CS-1-174

Ramos or 22rv1 cell lysate was prepared by sonication of washed cell pellets (30e6 cells) in PBS (4x 30 second pulses 10% Amplitude). Cell lysates were normalized to 1 mg/ml, aliquoted and stored at -80 °C until further usage.
Prior to experiments, 500 µl agarose Streptavidin beads (ThermoFisher, 20353) were pretreated with 1 mM CS-1-174 for 1 h at room temperature. After 6 washes with PBS containing 0.2% NP-40 beads were resuspended in 500 µl PBS and stored at 4 °C until usage.
Immediately before the pulldown experiments, cell lysates were thawed on ice and aliquotes of 1 mg protein were incubated with 50 µl pre-coated streptavidin beads at room temperature. After 1 h, beads were collected and washed twice with PBS. Bound proteins were eluted by boiling in 1x Laemmli buffer for 5 minutes and analyzed by SDS-PAGE and Western blot analysis.

Photoaffinity Labelling and Pull-Down

RAMOS cells (3e6 cells/ml) were treated with either DMSO vehicle or PAL probe (25 µM) for 1 hour in phenol red-free and serum-free medium and subsequently irradiated for 20 minutes at 365 nm. Cells were collected by centrifugation (400 rcf, 5 min), washed twice with PBS and lysed in RPIA-buffer supplemented with Benzonase (1:1000) and iodoacetamide (50 mM) and lysates were normalized to 2 mg/ml. CuAAC was performed to append biotin picolyl azide (100 µM final conc. Sigma-Aldrich, 900912). The click-reaction mixture was incubated at 37 °C for 60 minutes with agitation. Proteins were further processed using the SP2E protocol developed by Kielkowski and co-workers. [2] Proteins bound to magnetic streptavidin beads were

eluted by boiling in 8M Urea supplemented with 1x Laemmli buffer and 20 mM Biotin for 10 minutes, and subsequently analyzed by SDS-PAGE and Western blot analysis.

Luciferase Reporter Assay

Cells were seeded in white 96-well plates at 20,000 cells/well in 100 µL complete medium and allowed to adhere overnight. 10x stocks of the respective small molecule in complete medium were added to the wells to a final DMSO concentration of 1%. Cells were incubated for the indicated time before luciferase signal was measured using the Bright-Glo® Luciferase Assay System (Promega, E2620). In parallel, cell viability was assessed using CellTiter-Glo® 2.0 assay (Promega, G9242). Values for luciferase activity and viability were normalized to the DMSO and blank controls in Prism 10 (GraphPad), and curves were fit using a nonlinear regression. Additionally, the luciferase signal was normalized to viability.

MBD2 Lentiviral Knockdown Studies

Lentiviral stable MBD2 knockdown studies in T47D and 22rv1 cells were performed, as previously described. 2 µg of the following plasmids, shRNA construct of MBD2 (carrying a puromycin resistance gene, obtained from Vectorbuilder), psPAX2 (carrying GAG, REV, and pol genes), and pMD2G (carrying the VSVG pseudotyping gene), were dissolved in 1.2 mL of Gibco Opti-MEM Reduced Serum Medium (catalog no. 31985–062). In parallel, lipofectamine 2000 (Invitrogen, 11668019) was diluted in 1.2 mL of Gibco Opti-MEM Reduced Serum Medium. After 5 min, after which the two tubes were mixed and incubated for another 30 min at room temperature and subsequently the mixture was added to HEK293T cells at 30-40% confluence (cultured in DMEM + 10% heat inactivated FBS). The following day the medium was replaced to fresh DMEM + 10% heat inactivated FBS, and the cells were incubated for 48-72 h. On the day of infection, the virus containing medium was collected from the HEK293T cells filtered through a sterile 0.45 µm filter and combined with an equal volume of the target cell line media containing polybrene (Sigma-Aldrich, TR-1003-G). The lentiviral mixture was then added to the target cells and incubated for 24 h. Following a medium exchange to target cell line medium and 24 h incubation, infected cells were selected using puromycin (2.5 µg/ml) for 96 h followed by recovery in complete medium.

Western Blotting

Cells were lysed using RIPA buffer supplemented with Benzonase® Nuclease (1:1000; Millipore, 70746) for 15 min at 37°C and protein concentration was determined using Pierce™ BCA Protein Assay Kit (Thermo Fisher) according to the manufacturers protocol. The protein amount was normalized between the samples and proteins were separated on precast 4–20% Criterion TGX gels (Bio-Rad) followed by a transfer to a nitrocellulose membrane using the Trans-Blot Turbo transfer system (Bio-Rad). After the transfer, the membrane was blocked with Tris-buffered saline containing Tween 20 (TBST) containing 5% bovine serum albumin (BSA) for 1 h at RT. After blocking, target proteins were probed with primary antibodies in TBST with

5% BSA (primary antibody dilutions were performed according to the manufacturer). Incubation with primary antibodies was performed overnight at 4°C. After washing the membrane trice with TBST (5 min each) the membrane was incubated with IR680 (anti-mouse) or IR800 (anti-rabbit) conjugated secondary antibodies (1:10,000 dilution) for 1 h at RT. After 3 washes with TBST the membrane was imaged on a ChemiDoc Imaging System (Bio-Rad).
The following antibodies were used in this study: alpha-tubulin (mouse, Cell Signaling Technology, 3873S), GAPDH (mouse, proteintech, 60004-1-Ig), MBD2 (rabbit, Invitrogen, MA5-57472), Estrogen Receptor alpha (rabbit, Cell Signaling Technology, 8644S) Androgen Receptor (rabbit, Cell Signaling Technology, 5153S), IRDye 680RD goat anti-Mouse (LICOR 926–60870), and IRDye 800CW goat anti-rabbit (LICOR 926–32211).

RT-qPCR

20,000 cells were plated in 96-well tissue culture-treated plates (Corning 3513) and returned to the incubator to adhere overnight. The following day, compounds or DMSO control were added to the cells in biological triplicate, and plates were returned to the incubator for 24 h. The following day, medium was removed, and cells were lysed using Luna® Cell Ready Lysis Module (New England Biolabs, E3032S) according to the manufacturers protocol. RNA concentrations were determined using a NanoDrop Spectrophotometer (Thermo Fisher), and approx. 0.5 µg of RNA was used with Luna® Universal One-Step RT-qPCR Kit. RT-qPCR reactions were prepared in technical triplicate in TempPlate® 96-well PCR plates (USA Scientific) with 20 µL final volume. Thermal cycling was performed on a the CFX Connect Real-Time PCR Detection System (BioRad) by using the manufacturers recommendation. Relative fold-change was determined using the 2^-ddC_t method.
The following primers were used in this study: GAPDH (Hs.PT.39a.22214836), beta-Actin (Hs.PT.39a.22214847), ESR1 (Hs.PT.58.14846478), GREB1 (Hs.PT.58.26216464), MBD2 (Hs.PT.58.2050541), KLK3 (Hs.PT.58.20358116), FKBP5 (Hs.PT.58.39051416), CCNA2 (Hs.PT.56a.4535284), CDC20 (Hs.PT.58.20297042)

Quantitative Tandem Mass Tagging (TMT)-Based Proteomic Profiling

Cells were treated when they reached 70-80% confluency, with either DMSO vehicle or 12.5 µM of the respective TRACER for 24 hours. Total cell lysates were prepared using RIPA buffer supplemented with Benzonase® Nuclease (1:1000; Millipore, 70746) for 15 min at 37°C and protein concentration was determined using Pierce™ BCA Protein Assay Kit (Thermo Fisher) according to the manufacturers protocol. 100 µg of protein per replicate was reduced using 10 µM TCEP (37°C, 30 min) and alkylated using 20 µM iodoacetamide (room temperature, 30 min). Subsequently, protein samples were precipitated onto mixed hydrophilic/hydrophobic Sera-Mag™ Carboxylate-Modified Magnetic Beads (Cytiva) by the addition of EtOH to 80% (v/v). Beads were wasghed twice with 80% EtOH followed by resuspending the beads in 25 µl 8 M Urea in Tris-buffer pH 8.5. The sample was diluted to 2 M Urea and trypsin was added for overnight digestion (1:100, 37°C). After digestion, peptides were precipitated onto the magnetic beads by the addition of

acetonitrile to >95% (v/v). Beads were washed twice with acetonitrile and digested peptides were eluted using H_2O with 2% DMSO. 30 µg peptide per sample were labeled with TMTsixplex™ (Thermo Fisher Scientific, 90061), in accordance with the manufacturer's protocol. TMT samples were then consolidated and fractionated using high pH reversed-phase peptide fractionation kits (Thermo Fisher Scientific, 84868) according to the manufacturer's protocol. Fractions were vacuum concentrated, then reconstituted in 0.1% (v/v) formic acid and centrifuged at 20,000 g (5 min) in preparation for LC-MS/MS analysis.

Mass spectrometry analysis was performed on an Orbitrap Eclipse Tribrid Mass Spectrometer with a High Field Asymmetric Waveform Ion Mobility (FAIMS Pro) Interface (Thermo Fisher Scientific) with an UltiMate 3000 Nano Flow Rapid Separation LCnano System (Thermo Fisher Scientific). Offline fractionated samples (5 µL aliquot of 25 µL sample) were injected via an autosampler (Thermo Fisher Scientific) onto a 5 µL sample loop, which was subsequently eluted onto an Acclaim PepMap 100 C18 HPLC column (75 µm × 50 cm, NanoViper). The peptides were separated at a flow rate of 0.3 µL/min using the following gradient: 2% buffer B (100% acetonitrile with 0.1% formic acid) in buffer A (95:5 water/acetonitrile, 0.1% formic acid) for 5 min, followed by a gradient from 2−40% buffer B from 5−159 min, 40−95% buffer B from 159−160 min, held at 95% B from 160−179 min, 95% to 2% buffer B from 179−180 min, and then 2% buffer B from 180−200 min. The voltage applied to the nano-LC electrospray ionization source was 2.1 kV. Data were acquired through an MS1 master scan (Orbitrap analysis, resolution 120,000, 400−1800 m/z, RF lens 30%, heated capillary temperature 250 °C) with dynamic exclusion (repeat count 1, duration 60 sec). Data-dependent data acquisition comprised a full MS1 scan, followed by sequential MS2 scans based on 2 sec cycle times. FAIMS compensation voltages (CVs) of −35, −45, and −55 were applied. MS2 analysis consisted of a quadrupole isolation window of 0.7 m/z of the precursor ion followed by a higher energy collision dissociation (HCD) energy of 38% with an orbitrap resolution of 50,000.

Raw-files were analyzed using the Chaparral Platform and SagePro™. Trypsin cleavage specificity (cleavage at K, R, except if followed by P) allowed for up to 2 missed cleavages. Carbamidomethylation of cysteine residues (+57.02146) and TMT modification of peptide *N*-termini and lysine residues were set as static modification and methionine oxidation (+15.9949) was set as variable modification. MS1 tolerance was set to 10 ppm and MS2 tolerance to 300 ppm. Reporter-ion quantification was performed based on MS3 scans.

Cellular Treatments for RNA-seq, CUT&RUN, and ATAC-seq Experiments

T47D wild-type cells were grown in phenol-red-free RPMI supplemented with 10% charcoal-treated FBS (cFBS) for at 72h. The day before the experiment, cells were seeded into a 6-well tissue culture plate and cultured for 24 h. Afterwards, cells were treated with 1 nM E2 and DMSO vehicle or CS-1-103 (1.56 µM) for 24 h. 22Rv1 cells were cultured in 6-well tissue culture plates until 70-80% confluent and treated with DMSO vehicle or 12.5 µM CS-1-175 for 24 h in complete medium.

RNA-seq Analysis

T47D cells were cultured in phenol red free medium containing 10% carcoal stripped FBS for a minimum of four days prior to treatment for RNAseq. T47D or 22rv1 cells were treated with indicated compound concentrations and/or 1% DMSO for 24 h. For T47D cells, the treatment medium was additionally supplemented with 1 nM E2.

After treatment, RNA was isolated using the Monarch® Total RNA Miniprep Kit (NEB, T2010S). Total RNA quality, as well as poly-dT enriched mRNA quality, were assessed on an Agilent 2100 Bioanalyzer. Libraries were prepared using the KAPA mRNA Hyper Prep kit (Roche KK8581). Truncated universal stub adapters were ligated to cDNA fragments, which were then extended using 10 cycles of PCR using unique dual indexing primers into full length Illumina libraries. Library quality was checked on an AATI (now Agilent) Fragment Analyzer and transferred to the Vincent J. Coates Genomics Sequencing Laboratory (GSL), another QB3-Berkeley Core Research Facility at UC Berkeley.

FASTQ raw files were aligned and quantified using Kallisto[3] and filtered for >10 transcripts per million (tpm) for all replicates of at least one condition followed by differential gene expression analysis using DESeq2[4] and a threshold of |log2(FoldChange)| > 1 and an adjusted *p*-value < 0.05 was applied to identify differentially expressed genes. fGSEA analysis[5] was performed to identify significantly regulated MSigDB Hallmark gene sets[6]. Significantly downregulated genes were additionally compared to the ChEA 2022 TF-target dataset using Enrichr[7,8].

CUT&RUN

ER-binding sites in T47D were mapped using a modified CUT&RUN protocol described previously[9] with the anti-ESR1 (Epicypher, #13-2011) antibody. Sequenced CUT&RUN reads were processed as described by Skene et al.[10]

ATAC-seq analysis

Reads were counted over merged IDR-conservative ATAC-seq peaks in T47D across treatments, and peaks passing CPM > 5 in at least one sample group were retained. Differential expression was conducted using DEseq2. For visualization, a volcano plot was generated from filtered peaks, highlighting the top 500 downregulated peaks by p-value. Motif analysis on the top 500 downregulated peaks was performed using HOMER (size=given) with HOMER-generated background.

DEseq2 (1.46.0) was used for differential analysis. Rsubread (2.20.0) was used to count reads in peaks. HOMER (5.1) was used for motif analyses.

Integrative data analysis

Differential analysis results from ATAC-seq were annotated for overlap with high-confidence ER CUT&RUN sites (IDR-conservative – p-value ≥ 10-10). Differential expression from RNA-seq was integrated by joining promoter-annotated ATAC peaks to genes, producing a promoter-centric dataset. Binary indicators were defined as follows: ER binding site = 1 for overlaps; ATAC up or down = 1 if p-value ≤ 10-2 with sign determining the direction; RNA up or down = 1 if p-value ≤ 10-5 with sign determining the direction. Promoter entries with at least two active indicators (row sum ≥ 2) were retained and visualized with an UpSet plot to summarize intersections among ER binding and ATAC/RNA changes. For motif analysis, promoters were grouped by their combined indicator categories; HOMER was run with size="given" using a background of promoters lacking ER binding and nonsignificant in both ATAC (p-value > 5*10-2) and RNA (p-value > 5*10-

2). Known-motif enrichments from ER-containing categories were combined, filtered to motifs meeting rank ≤ 5 and p-value ≤ 10−10, reshaped to a motif-by-category matrix (log odds ratios), and correlated with the binary category design to yield a point-biserial correlation matrix.
ComplexHeatmap (2.22.0) was used to plot the correlation heatmap. UpSetR (1.4.0) was used to plot the Upset plot. ggplot2 (4.0.0), cowplot (1.2.0) and ggtext (0.1.2) were used to plot the volcano plot. HOMER (5.1) was used for motif analyses.

Synthetic Methods and Characterization

General Considerations

All chemical reactions were carried out under air with non-dry solvents, unless otherwise noted. Reagents were purchased at the highest commercial quality and used without further purification, unless otherwise stated.
Room temperature is defined as between 19-22 °C.
Reactions were stirred magnetically and monitored by thin layer chromatography (TLC) using TLC plates precoated with silica gel 60 F254 on aluminium (Merck KGaA). Detection was by UV (254 nm and 365 nm) or chemical stain (KMnO4, ninhydrin, iodine).
Solvents were removed in vacuo using a Buchi R-300 Rotavapor (equipped with an I-300 Pro Interface, B-300 Base Heating Bath, Welch 2037B-01 DryFast pump, and VWR AD15R-40-V11B Circulating Bath).
Automated flash chromatography was performed on a Biotage® Selekt instrument, equipped with a UV detector. Chromatograms were recorded at 254 and 280 nm.
High-resolution mass spectra (HRMS) were obtained on a Q Exactive Plus mass spectrometer (Thermo Fisher Scientific).
^{1}H and ^{13}C Nuclear Magnetic Resonance (NMR) spectra were recorded on BRUKER NEO or a JEOUL spectrometer operating at 500 or 400 MHz for ^{1}H and at 126 or 101 MHz for ^{13}C NMR, respectively. Measurements were carried out at ambient temperature. Chemical shifts (δ) are reported in ppm with the residual solvent signal as internal standard (chloroform at 7.26 and 77.2 ppm for ^{1}H NMR and ^{13}C NMR, respectively). The multiplicity of each signal is indicated as s = singlet, d = doublet, t = triplet, q = quartet, quin = quintet, m = multiplet (i.e. complex peak obtained due to overlap). Coupling constants (J) are reported in Hertz (Hz). ^{13}C NMR spectra were recorded with broadband ^{1}H decoupling. Product peaks overlapping with the solvent signal were determined using two-dimensional ^{1}H-^{13}C-HSQC NMR.
UPLC-UV/MS traces were recorded on either a Waters H-class or an Agilent 1290 Infinity II instrument. The Waters instrument was equipped with a quaternary solvent manager, a Waters autosampler, a Waters TUV detector and a Waters Acquity QDa detector with an Acquity UPLC BEH C18 1.7 µm, 2.1 x 50 mm RP column (Waters Corp., USA). Both instruments use A: 0.1 % TFA in H2O; B: 0.1% TFA in MeCN and
all methods have a flow rate of 0.6 mL/min. If not specified, the following gradient was used: 5% B 0.0 - 0.5 min, 5-95% B 0.5 - 3.0 min, 95% B 3.0 - 3.9 min, 5% B 3.9 - 5.0 min.

General Procedure A (Amide coupling)

A mixture of the corresponding carboxylic acid and HATU (1 equiv.) was dissolved in N,N-dimethylformamide (DMF) (0.1 M) followed by the addition of TEA (5 equiv.) was added and the reaction mixture stir for 2 minutes. The corresponding amine (1 equiv.) was dissolved in DMF and added to the reaction mixture. After 2 h at room temperature the reaction was purified by silica gel flash chromatography using a ternary gradient (Hexanes->Etylacetate->Methanol) on a Biotage® Selekt.

General Procedure B (Boc/tBu deprotection)

The Boc/tBu protected compound was dissolved in a minimal amount of MeOH and 4 M HCl in dioxane was added dropwise. After 1 h at r.t. the solvent was removed under a stream of nitrogen to obtain the desired compound as an HCl-salt. Deprotected compounds were used without further purification.

2-((((13*S*,*E*)-3-hydroxy-13-methyl-6,7,8,9,11,12,13,14,15,16-decahydro-17*H*-cyclopenta[*a*]phenanthren-17-ylidene)amino)oxy)acetic acid (**CS-1-65**):

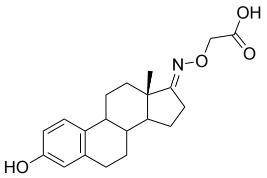

Estrone (500.0 mg, 1.85 mmol) and O-(carboxylmethyl)hydroxylamine (610 mg, 5.5 mmol) were dissolved in 16 ml dry pyridine and stirred overnight at room temperature. Afterwards the mixture was poured into 60 ml 10% HCl and extracted with EtOAc (3x 50 ml). The combined organic layers were washed with brine and dried over $MgSO_4$, filtered and evaporation before purification of the residue by flash column chromatography (DCM/MeOH 0-20%) to yield 605 mg of the desired product as a white powder (89% yield). Spectral data matched reported values from the literature.

^{1}H NMR (400 MHz, METHANOL-*D_4*) δ 7.57 (d, *J* = 2.9 Hz, 1H), 7.06 (d, *J* = 8.3 Hz, 1H), 6.55 (dd, *J* = 8.4, 2.7 Hz, 1H), 6.49 (s, 1H), 4.50 (t, *J* = 4.5 Hz, 2H), 2.81 – 2.74 (m, 1H), 2.56 (t, *J* = 6.1 Hz, 2H), 2.45 – 2.25 (m, 1H), 2.18 (d, *J* = 9.7 Hz, 1H), 2.06 – 1.83 (m, 3H), 1.71 – 1.25 (m, 7H), 0.91 (d, *J* = 2.9 Hz, 3H).
^{13}C NMR (101 MHz, METHANOL-*D_4*) δ 173.06, 156.57, 154.52, 137.67, 131.15, 126.15, 115.08, 112.68, 69.72, 52.89, 44.53, 43.97, 38.31, 33.93, 29.45, 27.24, 26.18, 25.98, 22.82, 16.87.

4-((4-(pyridin-2-yl)thiazol-2-yl)amino)phenol (**CS-1-69**):

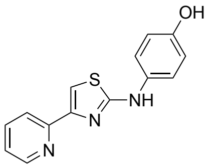

The target compound was synthesized via the Hantzsch thiazole synthesis. 2-bromo-1-(pyridin-2-yl)ethan-1-one (1 mmol) and 1-(4-hydroxyphenyl)thiourea (1 mmol) were dissolved in 20 ml EtOH (20 mL) and stirred at reflux for 1 h. The mixture was cooled to r.t., diluted with water (50 mL), and the pH was adjusted to approx. 8. After 2 h, the formed precipitate was filtered, washed with water and purified by flash chromatography (Hex/EtOAc 0−50%) to give the thiazole in 95% yield. (255 mg)

^{1}H NMR (400 MHz, $CDCl_3$/METHANOL-*D_4*) δ 8.47 (d, *J* = 2.7 Hz, 1H), 7.97 (d, *J* = 8.0 Hz, 1H), 7.79 (td, *J* = 7.8, 1.8 Hz, 1H), 7.34 (d, *J* = 8.7 Hz, 2H), 7.25 (s, 1H), 7.22 (d, *J* = 6.0 Hz, 1H), 6.81 (d, *J* = 8.9 Hz, 2H).
^{13}C NMR (101 MHz, $CDCl_3$/METHANOL-*D_4*) δ 167.74, 153.59, 153.06, 150.58, 149.16, 138.11, 133.82, 123.09, 121.95, 121.78 (2C), 116.30 (2C), 106.17.
HRMS for $C_{14}H_{11}N_3OS$ $[M+H]^+$ calc.: 270.0696 Da; found: 270.0691 Da

benzyl(6-(2-((((13*S*,*E*)-3-hydroxy-13-methyl-6,7,8,9,11,12,13,14,15,16-decahydro-17*H*-cyclopenta[*a*] phenanthren-17-ylidene)amino)oxy)acetamido)hexyl)carbamate (**CS-1-96**):

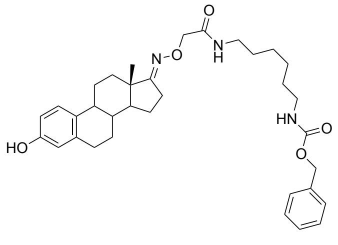

Was prepared according to **General Procedure A** starting from **CS-1-65** (10 mg) and benzyl (6-aminohexyl)carbamate. (3 mg, 18% yield)

^{1}H NMR (400 MHz, DMSO-D_6) δ 9.00 (s, 1H), 7.95 (s, 1H), 7.39 (t, *J* = 5.8 Hz, 1H), 7.37 – 7.24 (m, 6H), 7.20 (t, *J* = 5.6 Hz, 1H), 7.04 (d, *J* = 8.5 Hz, 1H), 6.50 (dd, *J* = 8.5, 3.0 Hz, 1H), 6.44 (s, 1H), 4.97 (s, 2H), 4.30 (s, 2H), 3.09 (q, *J* = 6.8 Hz, 2H), 2.96 (q, *J* = 6.6 Hz, 2H), 2.28 (d, *J* = 10.3 Hz, 1H), 2.14 (s, 1H), 1.85 (s, 3H), 1.53 – 1.42 (m, 2H), 1.37 (s, 11H), 1.23 (s, 4H), 0.87 (s, 3H).
^{13}C NMR (101 MHz, DMSO-D_6) δ 171.92, 169.41, 162.84, 156.60, 155.53, 137.84, 137.58, 130.55, 128.85, 128.24, 126.55, 115.47, 113.31, 72.78, 65.58, 52.87, 44.59, 44.04, 38.78, 38.52, 38.33, 36.31, 34.48, 31.31, 29.95, 29.75, 29.58, 26.49, 26.35, 23.06, 17.65.
HRMS for $C_{34}H_{45}N_3O_5$ $[M+H]^+$ calc.: 576.3432 Da; found: 576.3434 Da

N-(2-(3-(but-3-yn-1-yl)-3*H*-diazirin-3-yl)ethyl)-3-((4-(pyridin-2-yl)thiazol-2-yl)amino) benzamide (**CS-1-102**):

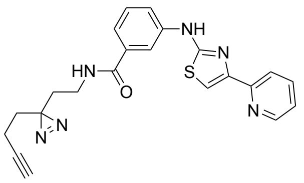

Was prepared according to **General Procedure A** starting from 3-((4-(pyridin-2-yl)thiazol-2-yl)amino)benzoic acid (8 mg) and 2-(3-(but-3-yn-1-yl)-3*H*-diazirin-3-yl)ethan-1-amine. (7 mg, 61% yield)

^{1}H NMR (400 MHz, DMSO-D_6) δ 10.42 (s, 1H), 8.55 (d, *J* = 4.6 Hz, 1H), 8.39 (t, *J* = 5.6 Hz, 1H), 8.13 (t, *J* = 2.0 Hz, 1H), 7.99 (d, *J* = 7.9 Hz, 1H), 7.95 – 7.80 (m, 2H), 7.54 (s, 1H), 7.48 – 7.33 (m, 2H), 7.33 – 7.23 (m, 1H), 3.14 (q, *J* = 6.7 Hz, 2H), 2.79 (t, *J* = 2.6 Hz, 1H), 2.65 (s, 1H), 1.98 (td, *J* = 7.4, 2.6 Hz, 2H), 1.62 (dt, *J* = 12.7, 7.2 Hz, 4H).
^{13}C NMR (101 MHz, DMSO-D_6) δ 166.99, 163.75, 152.62, 150.88, 149.96, 141.73, 137.76, 136.17, 129.51, 123.22, 120.92, 120.12, 119.85, 116.62, 107.68, 83.70, 72.32, 34.97, 32.53, 31.85, 27.86, 13.25.
HRMS for $C_{22}H_{21}N_6OS$ $[M+H]^+$ calc.: 417.1492 Da; found: 417.1456 Da

2-((((13*S*,*E*)-3-hydroxy-13-methyl-6,7,8,9,11,12,13,14,15,16-decahydro-17*H*-cyclopenta [*a*]phenanthren-17-ylidene)amino)oxy)-*N*-(6-(4-((4-(pyridin-2-yl)thiazol-2-yl)amino) phenoxy)hexyl)acetamide (**CS-1-85**):

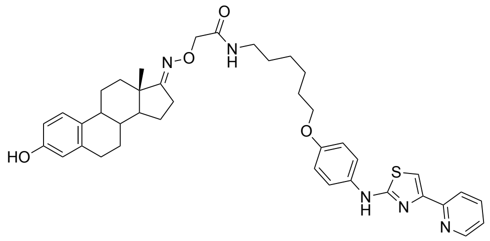

Was prepared according to **General Procedure A** starting from **CS-1-65** and **CS-1-71**. (6.3 mg, 63% yield)

^{1}H NMR (500 MHz, DMSO) δ 10.10 (s, 1H), 8.57 (dt, *J* = 4.7, 1.4 Hz, 1H), 8.28 (s, 1H), 8.05 – 7.95 (m, 1H), 7.88 (td, *J* = 7.7, 1.8 Hz, 1H), 7.72 – 7.55 (m, 2H), 7.47 (s, 1H), 7.42 (t, *J* = 6.0 Hz, 1H), 7.31 (ddd, *J* = 7.5, 4.8, 1.2 Hz, 1H), 7.02 (d, *J* = 8.5 Hz, 1H), 6.94 – 6.87 (m, 2H), 6.49 (dd, *J* = 8.4, 2.7 Hz, 1H), 6.43 (d, *J* = 2.6 Hz, 1H), 4.31 (s, 2H), 3.92 (t, *J* = 6.5 Hz, 2H), 3.13 (dq, *J* = 13.4, 6.6 Hz, 2H), 2.80 – 2.64 (m, 2H), 2.55 (s, 2H), 2.28 (dd, *J* = 13.9, 3.6 Hz, 1H), 2.14 (t, *J* = 10.3 Hz, 1H), 1.86 (ddt, *J* = 17.4, 14.1, 3.5 Hz, 2H), 1.69 (p, *J* = 6.7 Hz, 2H), 1.56 – 1.22 (m, 14H), 0.87 (s, 3H).
HRMS for $C_{40}H_{47}N_5O_4S$ $[M+H]^+$ calc.: 694.3422 Da; found: 694.3416 Da
Retention time Gradient A: 2.675 min

2-((((13*S*,*E*)-3-hydroxy-13-methyl-6,7,8,9,11,12,13,14,15,16-decahydro-17*H*-cyclopenta [*a*]phenanthren-17-ylidene)amino)oxy)-*N*-(2-(2-(2-(2-(4-((4-(pyridin-2-yl)thiazol-2-yl)amino)phenoxy)ethoxy)ethoxy)ethoxy) ethyl)acetamide (**CS-1-86**):

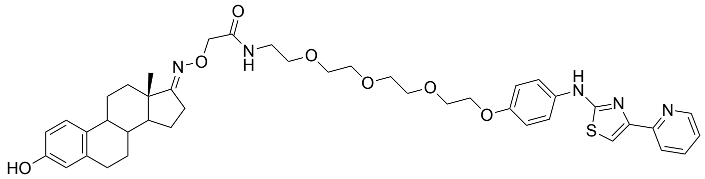

Was prepared according to **General Procedure A** starting from **CS-1-65** and **CS-1-76**. (10 mg, 51% yield)

^{1}H NMR (400 MHz, DMSO-*D*$_6$) δ 10.06 (s, 1H), 8.95 (s, 1H), 8.53 (d, *J* = 4.6 Hz, 1H), 7.93 (d, *J* = 7.9 Hz, 1H), 7.84 (td, *J* = 7.7, 1.7 Hz, 1H), 7.59 (d, *J* = 9.0 Hz, 2H), 7.43 (s, 1H), 7.35 (t, *J* = 5.6 Hz, 1H), 7.31 – 7.22 (m, 1H), 6.99 (d, *J* = 8.5 Hz, 1H), 6.90 (d, *J* = 9.0 Hz, 2H), 6.46 (dd, *J* = 8.4, 2.4 Hz, 1H), 6.39 (d, *J* = 2.4 Hz, 1H), 4.29 (s, 2H), 4.01 (d, *J* = 4.8 Hz, 2H), 3.69 (dd, *J* = 5.3, 3.7 Hz, 2H), 3.59 – 3.44 (m, 8H), 3.39 (t, *J* = 6.0 Hz, 2H), 3.29 – 3.18 (m, 2H), 3.13 (d, *J* = 5.2 Hz, 1H), 2.74 – 2.64 (m, 2H), 2.32 – 2.19 (m, 1H), 2.10 (t, *J* = 8.1 Hz, 1H), 1.89 – 1.76 (m, 3H), 1.51 – 1.41 (m, 1H), 1.40 – 1.20 (m, 6H), 0.83 (s, 3H).
^{13}C NMR (101 MHz, $CDCl_3$) δ 170.65, 169.16, 155.58, 154.08, 153.97, 149.39, 143.32, 138.16, 137.12, 136.96, 133.83, 126.47 (2C), 122.63, 121.76 (2C), 120.99, 118.67, 115.65 (2C), 113.23, 106.03, 72.69, 70.88, 70.68, 70.60, 70.36, 70.11, 69.88, 67.91, 53.00, 44.73, 44.65, 44.03, 38.76, 38.24, 36.58, 34.16, 29.55, 27.31, 26.19, 23.09.
HRMS for $C_{42}H_{51}N_5O_7S$ $[M+H]^+$ calc.: 769.3509 Da; found: 770.3580 Da

N-(6-(2-((((13*S*,*E*)-3-hydroxy-13-methyl-6,7,8,9,11,12,13,14,15,16-decahydro-17*H*-cyclopenta [*a*]phenanthren-17-ylidene)amino)oxy)acetamido)hexyl)-3-((4-(pyridin-2-yl)thiazol-2-yl)amino)benzamide (**CS-1-103):**

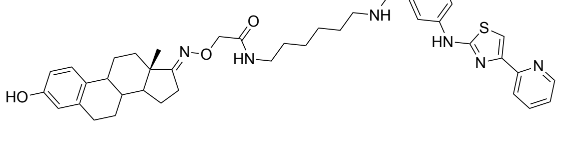

Was prepared according to **General Procedure A** starting from **CS-1-65** and **CS-1-99**. (3.4 mg, 47% yield)

^{1}H NMR (400 MHz, DMSO-D_6) δ 10.43 (s, 1H), 8.55 (d, *J* = 4.9 Hz, 1H), 8.36 (d, *J* = 5.0 Hz, 2H), 8.15 (s, 1H), 7.99 (d, *J* = 7.9 Hz, 1H), 7.94 – 7.78 (m, 2H), 7.53 (s, 1H), 7.37 (dd, *J* = 14.1, 5.7 Hz, 3H), 7.28 (dd, *J* = 7.5, 4.8 Hz, 1H), 6.96 (d, *J* = 8.4 Hz, 1H), 6.44 (dd, *J* = 8.5, 2.6 Hz, 1H), 6.39 (d, *J* = 2.7 Hz, 1H), 4.27 (s, 2H), 3.21 (q, *J* = 6.6 Hz, 2H), 3.07 (q, *J* = 6.5 Hz, 2H), 2.75 – 2.59 (m, 2H), 2.50 (m, 2H), 2.23 (d, *J* = 12.9 Hz, 1H), 2.08 (s, 2H), 1.87 – 1.73 (m, 3H), 1.48 (dt, *J* = 10.4, 5.3 Hz, 3H), 1.43 – 1.21 (m, 11H), 0.82 (s, 3H).
^{13}C NMR (101 MHz, DMSO-D_6) δ 171.93, 169.46, 166.87, 163.75, 155.52, 152.64, 149.94, 142.20, 141.67, 137.73, 137.55, 136.42, 132.45, 130.61, 126.53, 123.21, 120.91, 120.11, 119.66, 116.64, 115.46, 113.31, 107.62, 76.04, 52.84, 44.58, 44.02, 38.51, 38.31, 34.46, 29.74 (3C), 29.56, 27.74, 27.37, 26.74, 26.51, 26.34 (2C), 17.64.
HRMS for $C_{41}H_{49}N_6O_4S$ $[M+H]^+$ calc.: 721.3531 Da; found: 721.3525 Da

2-((((13*S*,*E*)-3-hydroxy-13-methyl-6,7,8,9,11,12,13,14,15,16-decahydro-17*H*-cyclopenta [*a*]phenanthren-17-ylidene)amino)oxy)-1-(4-(3-((4-(pyridin-2-yl)thiazol-2-yl)amino) benzoyl)piperazin-1-yl)ethan-1-one (**CS-1-163**):

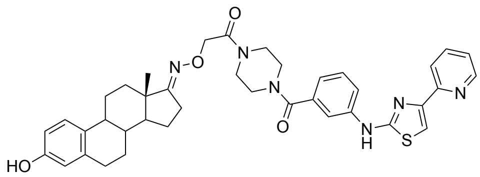

Was prepared according to **General Procedure A** starting from **CS-1-65** and **CS-1-158**. (5.6 mg, 81% yield)

^{1}H NMR (500 MHz, DMSO) δ 10.51 (s, 1H), 9.02 (s, 1H), 8.59 (dd, *J* = 5.0, 1.6 Hz, 1H), 7.99 (d, *J* = 7.8 Hz, 1H), 7.92 (td, *J* = 7.7, 1.8 Hz, 1H), 7.82 (d, *J* = 8.0 Hz, 2H), 7.62 (s, 1H), 7.45 (t, *J* = 7.9 Hz, 1H), 7.34 (dd, *J* = 7.4, 4.8 Hz, 1H), 7.03 (d, *J* = 7.9 Hz, 2H), 6.51 (dd, *J* = 8.4, 2.6 Hz, 1H), 6.44 (d, *J* = 2.6 Hz, 1H), 4.65 (s, 2H), 3.64 – 3.46 (m, 4H), 2.80 – 2.67 (m, 2H), 2.44 (s, 1H), 2.27 (d, *J* = 13.0 Hz, 1H), 2.14 (s, 1H), 1.94 – 1.73 (m, 4H), 1.55 – 1.20 (m, 6H), 0.85 (s, 3H). (Proton signal of two piperazine CH_2-groups overlaps with H_2O peak)
HRMS for $C_{39}H_{42}N_6O_4S$ $[M+H]^+$ calc.: 691.3061 Da; found: 691.3054 Da
Retention time Gradient A: 2.403 min

N-(5-(2-((((13*S*,*E*)-3-hydroxy-13-methyl-6,7,8,9,11,12,13,14,15,16-decahydro-17*H*-cyclopenta [*a*]phenanthren-17-ylidene)amino)oxy)acetamido)pentyl)-3-((4-(pyridin-2-yl)thiazol-2-yl)amino)benzamide (**CS-1-164**):

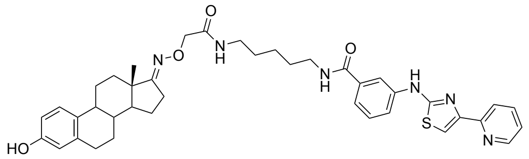

Was prepared according to **General Procedure A** starting from **CS-1-65** and **CS-1-157**. (5.2 mg, 73% yield)

^{1}H NMR (500 MHz, DMSO) δ 10.47 (s, 1H), 8.66 – 8.56 (m, 1H), 8.41 (t, *J* = 5.6 Hz, 1H), 8.31 (s, 1H), 8.19 (d, *J* = 2.4 Hz, 1H), 8.03 (d, *J* = 7.8 Hz, 1H), 7.98 – 7.85 (m, 2H), 7.57 (s, 1H), 7.43 (dd, *J* = 13.7, 6.1 Hz, 3H), 7.32 (dd, *J* = 7.6, 4.5 Hz, 1H), 7.01 (d, *J* = 8.6 Hz, 1H), 6.51 – 6.41 (m, 2H), 4.31 (s, 2H), 3.26 (q, *J* =

6.8 Hz, 3H), 3.13 (q, *J* = 6.7 Hz, 2H), 2.76 – 2.57 (m, 3H), 2.34 – 2.22 (m, 1H), 2.13 (s, 1H), 1.93 – 1.77 (m, 4H), 1.60 – 1.24 (m, 12H), 0.86 (s, 3H).
HRMS for $C_{40}H_{46}N_6O_4S$ $[M+H]^+$ calc.: 707.3374 Da; found: 707.3363 Da
Retention time Gradient A: 2.408 min

N-(3-(2-((((13*S*,*E*)-3-hydroxy-13-methyl-6,7,8,9,11,12,13,14,15,16-decahydro-17*H*-cyclopenta [*a*]phenanthren-17-ylidene)amino)oxy)acetamido)propyl)-3-((4-(pyridin-2-yl)thiazol-2-yl)amino)benzamide (**CS-1-165**):

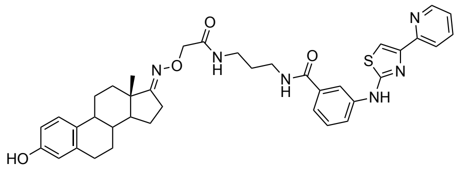

Was prepared according to **General Procedure A** starting from **CS-1-65** and **CS-1-160**. (5.3 mg, 78% yield)

^{1}H NMR (500 MHz, DMSO) δ 10.47 (s, 1H), 8.59 (dd, *J* = 4.9, 1.7 Hz, 1H), 8.48 (t, *J* = 5.8 Hz, 1H), 8.28 (d, *J* = 12.4 Hz, 2H), 8.03 (d, *J* = 7.9 Hz, 1H), 7.97 – 7.82 (m, 2H), 7.68 (t, *J* = 6.2 Hz, 1H), 7.58 (s, 1H), 7.41 (d, *J* = 7.4 Hz, 2H), 7.32 (dd, *J* = 7.6, 4.8 Hz, 1H), 6.97 (d, *J* = 8.5 Hz, 1H), 6.48 (dd, *J* = 8.4, 2.7 Hz, 1H), 6.41 (d, *J* = 2.6 Hz, 1H), 4.36 (s, 2H), 3.35 – 3.29 (m, 2H), 3.21 (q, *J* = 6.4 Hz, 2H), 2.76 – 2.57 (m, 3H), 2.23 (dq, *J* = 13.2, 3.7 Hz, 1H), 2.08 (d, *J* = 11.8 Hz, 1H), 1.94 – 1.74 (m, 3H), 1.67 (p, *J* = 6.8 Hz, 2H), 1.50 (td, *J* = 13.2, 4.0 Hz, 1H), 1.43 – 1.18 (m, 5H), 0.85 (s, 3H).
HRMS for $C_{38}H_{42}N_6O_4S$ $[M+H]^+$ calc.: 679.3061 Da; found: 679.3057 Da
Retention time Gradient A: 2.329 min

N-(12-(2-((((13*S*,*E*)-3-hydroxy-13-methyl-6,7,8,9,11,12,13,14,15,16-decahydro-17*H*-cyclopenta [*a*]phenanthren-17-ylidene)amino)oxy)acetamido)dodecyl)-3-((4-(pyridin-2-yl)thiazol-2-yl)amino)benzamide (**CS-1-166**):

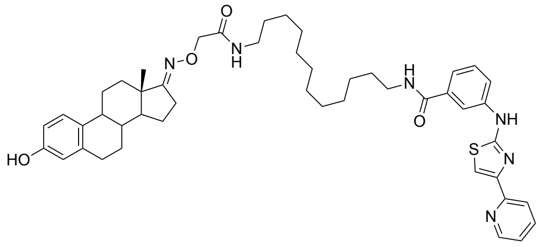

Was prepared according to **General Procedure A** starting from **CS-1-65** and **CS-1-159**. (4.3 mg, 51% yield)

^{1}H NMR (400 MHz, DMSO-D_6) δ 10.42 (s, 1H), 8.55 (d, *J* = 4.8 Hz, 1H), 8.44 – 8.31 (m, 1H), 8.15 (s, 1H), 7.99 (d, *J* = 7.9 Hz, 1H), 7.93 – 7.79 (m, 2H), 7.53 (s, 1H), 7.45 – 7.23 (m, 4H), 6.99 (d, *J* = 8.5 Hz, 1H), 6.47 (dd, *J* = 8.4, 2.6 Hz, 1H), 6.40 (d, *J* = 2.6 Hz, 1H), 4.26 (s, 2H), 3.20 (t, *J* = 6.6 Hz, 2H), 3.06 (ddq, *J* = 19.6, 13.1, 6.6 Hz, 2H), 2.70 (d, *J* = 13.7 Hz, 2H), 2.51 (d, *J* = 8.6 Hz, 1H), 2.24 (d, *J* = 13.0 Hz, 1H), 2.09 (d, *J* = 9.9 Hz, 1H), 1.89 – 1.73 (m, 3H), 1.52 – 1.43 (m, 3H), 1.37 – 1.15 (m, 24H), 0.83 (s, 3H).
HRMS for $C_{44}H_{51}F_3N_8O_4S$ $[M+H]^+$ calc.: 845.3779 Da; found: 845.3772 Da
Retention time Gradient A: 2.962 min

2-((((13*S*,*E*)-3-hydroxy-13-methyl-6,7,8,9,11,12,13,14,15,16-decahydro-17*H*-cyclopenta[*a*]phenanthren-17-ylidene)amino)oxy)-1-(9-(3-((4-(pyridin-2-yl)thiazol-2-yl)amino)benzoyl)-3,9-diazaspiro[5.5]undecan-3-yl)ethan-1-one (**CS-1-167**):

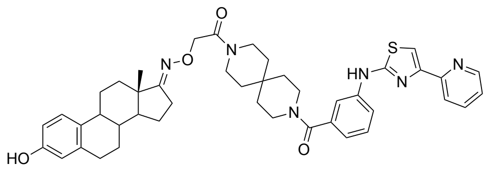

Was prepared according to **General Procedure A** starting from **CS-1-65** and **CS-1-161**. (2.9 mg, 62% yield)

^{1}H NMR (400 MHz, DMSO-*D*$_6$) δ 10.42 (s, 1H), 8.95 (s, 1H), 8.56 (d, *J* = 4.7 Hz, 1H), 7.92 (d, *J* = 7.8 Hz, 1H), 7.86 (t, *J* = 7.9 Hz, 1H), 7.77 (s, 1H), 7.72 (d, *J* = 8.1 Hz, 1H), 7.55 (s, 1H), 7.37 (t, *J* = 7.8 Hz, 1H), 7.29 (dd, *J* = 7.3, 4.8 Hz, 1H), 6.99 (d, *J* = 8.5 Hz, 1H), 6.93 (d, *J* = 7.5 Hz, 1H), 6.50 – 6.43 (m, 1H), 6.39 (d, *J* = 2.6 Hz, 1H), 4.56 (s, 2H), 3.59 (s, 2H), 3.40 – 3.33 (m, 5H), 2.84 – 2.63 (m, 2H), 2.50 (s, 1H), 2.43 – 2.35 (m, 1H), 2.31 – 2.20 (m, 1H), 2.10 (s, 1H), 1.89 – 1.71 (m, 3H), 1.59 – 1.17 (m, 18H), 0.82 (s, 3H).
HRMS for $C_{44}H_{50}N_6O_4S$ $[M+H]^+$ calc.: 759.3687 Da; found: 759.3679 Da
Retention time Gradient A: 2.530 min

N-(6-(4-(3-(4-cyano-3-(trifluoromethyl)phenyl)-5,5-dimethyl-4-oxo-2-thioxoimidazolidin-1-yl)butanamido)hexyl)-3-((4-(pyridin-2-yl)thiazol-2-yl)amino)benzamide (**CS-1-175):**

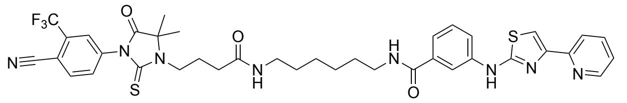

Was prepared according to **General Procedure A** starting from **CS-1-170** and **CS-1-99**. (8.6 mg, 56% yield)

^{1}H NMR (400 MHz, DMSO-*D*$_6$) δ 10.41 (s, 1H), 8.55 (d, *J* = 2.7 Hz, 1H), 8.38 (t, *J* = 5.7 Hz, 1H), 8.30 (d, *J* = 8.5 Hz, 1H), 8.19 (s, 1H), 8.14 (s, 1H), 7.98 (dd, *J* = 11.1, 7.9 Hz, 2H), 7.91 – 7.82 (m, 2H), 7.79 (t, *J* = 5.7 Hz, 1H), 7.54 (s, 1H), 7.38 (d, *J* = 7.1 Hz, 2H), 7.29 (dd, *J* = 7.8, 4.6 Hz, 1H), 3.67 – 3.58 (m, 2H), 3.22 (d, *J* = 6.4 Hz, 2H), 3.01 (q, *J* = 6.5 Hz, 2H), 2.13 (t, *J* = 7.4 Hz, 2H), 1.97 – 1.89 (m, 2H), 1.49 (s, 6H), 1.43 – 1.17 (m, 8H).
^{13}C NMR (101 MHz, DMSO-*D*$_6$) δ 178.73, 175.83, 171.63, 166.88, 163.76, 152.59, 150.83, 149.91, 141.68, 138.66, 137.79, 136.54, 136.44, 134.52, 131.50, 129.44, 128.59, 123.23, 122.76, 120.92, 120.14, 119.69, 116.65, 115.59, 108.91, 107.69, 65.72, 43.54, 38.96, 33.08, 29.68 (2C), 26.75, 26.73, 24.43 (2C), 22.71.
HRMS for $C_{38}H_{39}F_3N_8O_3S_2$ $[M+H]^+$ calc.: 777.2611 Da; found: 777.2606 Da

N-(2-(2-(2-(4-(3-(4-cyano-3-(trifluoromethyl)phenyl)-5,5-dimethyl-4-oxo-2-thioxoimidazolidin-1-yl)butanamido)ethoxy)ethoxy)ethyl)-3-((4-(pyridin-2-yl)thiazol-2-yl)amino)benzamide (**CS-1-176**):

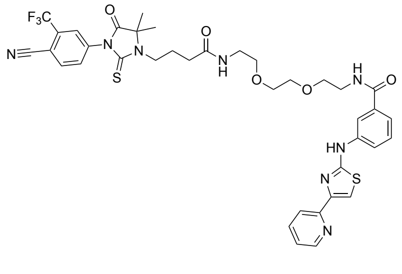

Was prepared according to **General Procedure A** starting from **CS-1-170** and **CS-1-138**. (9.8 mg, 61% yield)

^{1}H NMR (400 MHz, DMSO-*D*$_6$) δ 10.42 (s, 1H), 8.61 – 8.52 (m, 1H), 8.43 (t, *J* = 5.7 Hz, 1H), 8.30 (d, *J* = 8.2 Hz, 1H), 8.23 – 8.17 (m, 1H), 8.15 (t, *J* = 1.8

Hz, 1H), 7.98 (ddd, *J* = 12.3, 8.1, 1.5 Hz, 2H), 7.88 (dtd, *J* = 16.0, 6.9, 2.6 Hz, 2H), 7.54 (s, 1H), 7.46 – 7.36 (m, 2H), 7.35 – 7.25 (m, 1H), 3.68 – 3.57 (m, 2H), 3.56 – 3.44 (m, 6H), 3.39 (dt, *J* = 12.0, 5.8 Hz, 4H), 3.17 (q, *J* = 5.8 Hz, 2H), 2.14 (t, *J* = 7.5 Hz, 2H), 1.99 – 1.88 (m, 3H), 1.49 (s, 6H).

^{13}C NMR (101 MHz, DMSO-D_6) δ 178.73, 175.84, 171.96, 167.02, 163.74, 152.61, 150.86, 149.93, 141.73, 138.66, 137.79, 136.54, 136.04, 134.52, 131.50, 129.49, 128.59, 123.22, 122.76, 120.92, 120.11, 119.85, 116.71, 115.60, 108.91, 107.68, 70.09 (2C), 69.69, 69.46, 65.72, 43.51, 39.06, 32.97, 24.33, 22.70 (2C).

HRMS for $C_{38}H_{39}F_3N_8O_5S_2$ $[M+H]^+$ calc.: 809.2510 Da; found: 809.2509 Da

N-(12-(4-(3-(4-cyano-3-(trifluoromethyl)phenyl)-5,5-dimethyl-2,4-dioxoimidazolidin-1-yl)butanamido) dodecyl)-3-((4-(pyridin-2-yl)thiazol-2-yl)amino)benzamide (**CS-1-169**):

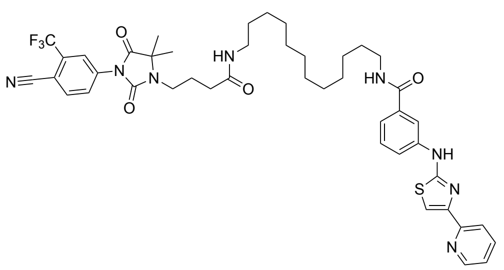

Was prepared according to **General Procedure A** starting from **CS-1-154**. (6.7 mg, 81% yield)

^{1}H NMR (400 MHz, DMSO-*D*$_6$) δ 10.41 (s, 1H), 8.55 (ddd, *J* = 4.8, 1.8, 0.9 Hz, 1H), 8.37 (t, *J* = 5.6 Hz, 1H), 8.26 (d, *J* = 8.4 Hz, 1H), 8.15 (d, *J* = 2.2 Hz, 2H), 7.99 (dt, *J* = 8.2, 1.8 Hz, 2H), 7.89 – 7.81 (m, 2H), 7.72 (t, *J* = 5.6 Hz, 1H), 7.53 (s, 1H), 7.46 – 7.33 (m, 2H), 7.33 – 7.23 (m, 1H), 3.30 – 3.15 (m, 4H), 2.97 (q, *J* = 6.6 Hz, 2H), 2.10 (t, *J* = 7.5 Hz, 2H), 1.90 – 1.72 (m, 2H), 1.60 – 1.45 (m, 2H), 1.42 (d, *J* = 4.5 Hz, 8H), 1.28 (d, *J* = 27.5 Hz, 6H), 1.18 (s, 10H).

^{13}C NMR (101 MHz, DMSO-*D*$_6$) δ 175.21, 171.81, 166.90, 163.74, 162.86, 153.06, 152.62, 150.86, 149.94, 141.67, 137.72, 137.39, 136.56, 136.45, 130.46, 129.42, 124.57, 123.19, 120.91, 120.13, 119.66, 116.62, 115.75, 107.64, 107.17, 62.16, 38.98, 33.11, 29.66 (2C), 29.53 (4C), 29.32, 29.29, 27.00, 26.95, 25.68, 23.06 (2C). two carbons are overlapping with the solvent peak.

HRMS for $C_{44}H_{51}F_3N_8O_4S$ $[M+H]^+$ calc.: 845.3779 Da; found: 845.3772 Da

N-(2-(2-(2-(4-(3-(4-cyano-3-(trifluoromethyl)phenyl)-5,5-dimethyl-2,4-dioxoimidazolidin-1-yl)butanamido) ethoxy)ethoxy)ethyl)-3-((4-(pyridin-2-yl)thiazol-2-yl)amino)benzamide (**CS-1-168**):

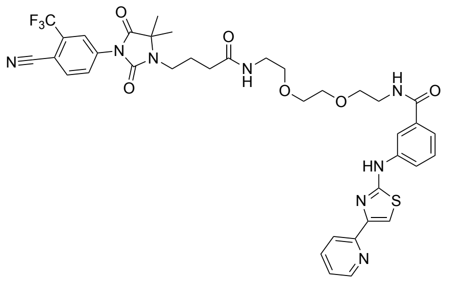

Was prepared according to **General Procedure A** starting from **CS-1-154**. (7.4 mg 92% yield)

^{1}H NMR (400 MHz, DMSO-*D*$_6$) δ 10.42 (s, 1H), 8.53 (dd, *J* = 4.9, 1.6 Hz, 1H), 8.43 (s, 1H), 8.23 (d, *J* = 8.2 Hz, 1H), 8.19 – 8.09 (m, 2H), 7.98 (td, *J* = 5.3, 2.5 Hz, 2H), 7.92 – 7.81 (m, 3H), 7.52 (s, 1H), 7.38 (d, *J* = 7.0 Hz, 2H), 7.28 (d, *J* = 12.4 Hz, 1H), 3.38 (dt, *J* = 16.2, 5.6 Hz, 4H), 3.27 (dt, *J* = 21.8, 7.5 Hz, 2H), 3.15 (q, *J* = 5.8 Hz, 2H), 2.11 (t, *J* = 7.5 Hz, 2H), 1.84 – 1.74 (m, 2H), 1.39 (s, 6H).

^{13}C NMR (101 MHz, DMSO-*D*$_6$) δ 175.26, 172.38, 167.17, 163.75, 157.3, 153.06, 152.57, 150.80, 149.91, 141.69, 137.85, 137.31, 136.53, 135.94, 131.47, 130.45, 129.54, 124.52, 123.31, 120.98, 120.10, 119.92, 116.62, 115.74, 107.68, 104.62, 70.04 (2C), 69.58, 69.38, 62.18, 39.04, 33.01, 25.56, 23.01(2C). (two carbon overlap with solvent peak)

HRMS for $C_{38}H_{39}F_3N_8O_6S$ $[M+H]^+$ calc.: 793.2738 Da; found: 793.2729 Da

N-(6-(4-(3-(4-cyano-3-(trifluoromethyl)phenyl)-5,5-dimethyl-2,4-dioxoimidazolidin-1-yl)butanamido)hexyl)-3-((4-(pyridin-2-yl)thiazol-2-yl)amino)benzamide (**CS-1-162**):

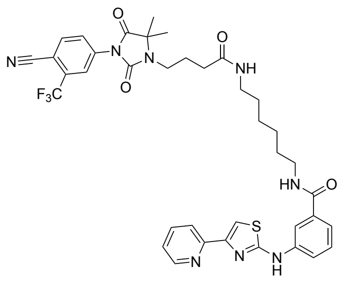

Was prepared according to **General Procedure A** starting from **CS-1-154**. (8.3 mg 73% yield**)**

^{1}H NMR (400 MHz, DMSO-D_6) δ 10.41 (s, 1H), 8.55 (dd, *J* = 4.9, 1.7 Hz, 1H), 8.38 (t, *J* = 5.6 Hz, 1H), 8.27 (dd, *J* = 8.5, 4.6 Hz, 1H), 8.19 – 8.11 (m, 2H), 8.05 – 7.96 (m, 2H), 7.95 – 7.80 (m, 2H), 7.75 (t, *J* = 5.6 Hz, 1H), 7.53 (s, 1H), 7.38 (d, *J* = 7.4 Hz, 2H), 7.28 (dd, *J* = 7.5, 5.0 Hz, 1H), 3.29 – 3.18 (m, 2H), 3.00 (q, *J* = 6.5 Hz, 2H), 2.11 (t, *J* = 7.6 Hz, 2H), 1.80 (q, *J* = 7.8 Hz, 3H), 1.54 – 1.23 (m, 14H).
^{13}C NMR (101 MHz, DMSO-D_6) δ 175.21, 171.83, 166.88, 163.75, 153.06, 152.62, 150.87, 149.95, 143.87, 141.67, 137.73, 137.40, 136.56, 136.42, 130.47, 129.43, 124.58, 124.14, 123.21, 120.90, 120.13, 119.67, 116.64, 115.75, 107.64, 107.18, 62.16, 38.92, 33.12, 29.67, 26.70 (2C), 25.70, 23.06 (2C). (two carbon overlap with solvent peak)
HRMS for $C_{38}H_{39}F_3N_8O_4S$ $[M+H]^+$ calc.: 761.2840 Da; found: 761.2834 Da

tert-butyl (6-(4-((4-(pyridin-2-yl)thiazol-2-yl)amino)phenoxy)hexyl)carbamate (**CS-1-71**):

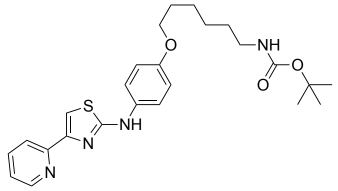

CS-1-69, *tert*-butyl (6-bromohexyl)carbamate (0.1 mmol each) and K_2CO_3 (0.6 mmol) were dissolved/suspended in MeCN (15 ml) and heated to reflux. Once TLC indicated full consumption of the starting material, the reaction was allowed to cool to r.t., precipitates were filtered off, the solvent was removed under reduced pressure and the crude was purified via flash chromatography (Hex/EtOAc, 0-80%) to obtain **CS-1-71** in 81% yield (38 mg). Before subsequent reactions, the compound was deprotected according to **General Procedure B** and used without further purification**.**

^{1}H NMR (400 MHz, CHLOROFORM-*D*) δ 8.49 (dd, *J* = 5.0, 1.6 Hz, 1H), 7.96 (s, 1H), 7.88 (d, *J* = 7.8 Hz, 1H), 7.60 (td, *J* = 7.7, 1.8 Hz, 1H), 7.30 – 7.22 (m, 3H), 7.08 (dd, *J* = 7.6, 4.8 Hz, 1H), 6.78 (d, *J* = 8.8 Hz, 2H), 3.83 (t, *J* = 6.4 Hz, 3H), 3.04 (d, *J* = 6.7 Hz, 2H), 1.67 (p, *J* = 6.5 Hz, 2H), 1.36 (s, 18H), 1.29 (d, *J* = 6.4 Hz, 3H).
^{13}C NMR (101 MHz, $CDCl_3$) δ 166.97, 156.13, 155.76, 152.75, 151.09, 149.38, 136.90, 133.80, 122.44, 121.79 (2C), 120.99, 115.37 (2C), 105.79, 79.14, 68.25, 40.60, 30.98, 29.26, 28.52 (3C), 26.62, 25.83.
HRMS for $C_{20}H_{24}N_4OS$ $[M+H]^+$ calc.: 469.2268 Da; found: 469.2266 Da
HRMS for $C_{20}H_{24}N_4OS$ $[M+H]^+$ calc.: 369.1744 Da; found: 369.1733 Da (after Boc deprotection)

tert-butyl (2-(2-(2-(2-(4-((4-(pyridin-2-yl)thiazol-2-yl)amino)phenoxy)ethoxy)ethoxy) ethoxy)ethyl)carbamate (**CS-1-76**):

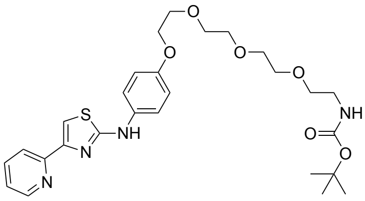

CS-1-69, *tert*-butyl (2-(2-(2-(2-bromoethoxy)ethoxy)ethoxy)ethyl) carbamate (0.1 mmol each) and K_2CO_3 (0.6 mmol) were dissolved/suspended in MeCN (15 ml) and heated to reflux. Once TLC indicated full consumption of the starting material, the reaction was allowed to cool to r.t., precipitates were filtered off, the solvent was removed under reduced pressure and the crude was purified via flash chromatography (Hex/EtOAc, 30-100%) to obtain **CS-1-76** in 77% yield (42 mg). Before subsequent reactions, the compound was deprotected according to **General Procedure B** and used without further purification.

^{1}H NMR (400 MHz, $CDCl_3$) δ 8.58 (d, *J* = 4.8 Hz, 1H), 7.95 (d, *J* = 7.8 Hz, 1H), 7.71 (t, *J* = 7.7 Hz, 1H), 7.54 (s, 1H), 7.34 – 7.32 (m, 3H), 7.21 – 7.14 (m, 1H), 6.91 (d, *J* = 8.9 Hz, 2H), 4.15 – 4.08 (m, 2H), 3.85 (t, *J* = 4.9 Hz, 2H), 3.76 – 3.50 (m, 10H), 3.30 (d, *J* = 5.3 Hz, 2H), 1.43 (s, 9H).
^{13}C NMR (101 MHz, $CDCl_3$) δ 166.67, 156.10, 155.52, 152.75, 151.21, 149.44, 136.91, 133.98, 122.48, 121.71, 120.96, 115.63, 105.91, 79.28, 70.93, 70.74, 70.67, 70.34, 70.30, 69.87, 67.90, 40.46, 28.52.
HRMS for $C_{27}H_{36}N_4O_6SNa^+$ $[M+Na]^+$ calc.: 567.2248 Da; found: 567.2236 Da

N-(6-aminohexyl)-3-((4-(pyridin-2-yl)thiazol-2-yl)amino)benzamide (**CS-1-99**):

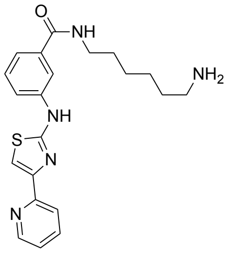

Was prepared according to **General Procedure A** starting from 3-((4-(pyridin-2-yl)thiazol-2-yl)amino)benzoic acid and *tert*-butyl (6-aminohexyl)carbamate. After purification of the Boc-protected intermediate, deprotection according to **General Procedure B** afforded **CS-1-99** as a yellow solid that was used without further purification. (115 mg, 89%)

^{1}H NMR (500 MHz, DMSO-D_6) δ 10.60 (s, 1H), 8.62 – 8.57 (m, 1H), 8.48 (t, *J* = 5.7 Hz, 1H), 8.43 (s, 1H), 8.24 – 8.18 (m, 1H), 8.03 (d, *J* = 7.8 Hz, 1H), 7.96 (dt, *J* = 7.0, 2.3 Hz, 1H), 7.89 (td, *J* = 7.7, 1.8 Hz, 1H), 7.58 (s, 1H), 7.48 – 7.38 (m, 2H), 7.33 (ddd, *J* = 7.6, 4.7, 1.3 Hz, 1H), 3.27 (q, *J* = 6.6 Hz, 2H), 2.75 (t, *J* = 7.5 Hz, 2H), 1.53 (tt, *J* = 11.0, 4.8 Hz, 4H), 1.42 – 1.30 (m, 4H).
^{13}C NMR (126 MHz, DMSO-D_6) δ 166.85, 163.72, 152.58, 150.79, 149.90, 141.67, 137.68, 136.30, 129.38, 123.16, 120.83, 120.04, 116.60, 107.58, 39.13, 29.44, 27.76, 26.46, 26.07. (one carbon overlaps with solvent peak)
HRMS for $C_{21}H_{26}N_5OS$ $[M+H]^+$ calc.: 396.1853 Da; found: 396.1844 Da

N-(2-(2-(2-aminoethoxy)ethoxy)ethyl)-3-((4-(pyridin-2-yl)thiazol-2-yl)amino)benzamide (**CS-1-138**):

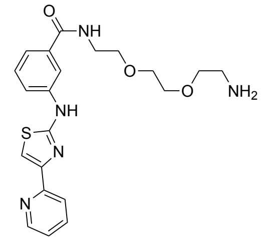

Was prepared according to **General Procedure A** starting from 3-((4-(pyridin-2-yl)thiazol-2-yl)amino)benzoic acid and *tert*-butyl (6-aminohexyl)carbamate. After purification of the Boc-protected intermediate, deprotection according to **General Procedure B** afforded **CS-1-138** as a yellow solid that was used without further purification. (145 mg, 86%)

^{1}H NMR (400 MHz, DMSO-D_6) δ 11.06 (s, 1H), 8.77 (d, *J* = 5.2 Hz, 1H), 8.66 (t, *J* = 5.7 Hz, 1H), 8.46 (s, 1H), 8.33 (s, 1H), 8.13 (d, *J* = 7.8 Hz, 2H), 8.08 (s, 1H), 7.82 (t, *J* = 5.4 Hz, 1H), 7.46 (dt, *J* = 15.3, 7.7 Hz, 2H), 3.56 (s, 6H), 3.44 (q, *J* = 5.6 Hz, 2H), 3.10 – 2.97 (m, 2H), 2.91 (q, *J* = 5.6 Hz, 2H).
^{13}C NMR (101 MHz, DMSO-D_6) δ 166.99, 164.51, 146.79, 145.21, 143.81, 141.20, 135.97, 129.62, 125.18, 120.83, 120.44, 116.80, 114.52, 106.23, 70.20, 69.95, 69.41, 67.13, 45.84, 38.99.
HRMS for $C_{21}H_{25}N_5O_3S$ $[M+H]^+$ calc.: 428.1751 Da; found: 428.1741 Da

N-(3-aminopropyl)-3-((4-(pyridin-2-yl)thiazol-2-yl)amino)benzamide (**CS-1-157**):

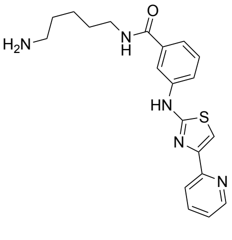

Was prepared according to **General Procedure A** starting from 3-((4-(pyridin-2-yl)thiazol-2-yl)amino)benzoic acid and *tert*-butyl (5-pentyl)carbamate. After purification of the Boc-protected intermediate, deprotection according to **General Procedure B** afforded **CS-1-159** as a yellow solid that was used without further purification. (168 mg, 88%) A small portion was purified via semipreparative HPLC for analytical purposes.

^{1}H NMR (500 MHz, DMSO-D_6) δ 10.57 (s, 1H), 8.63 – 8.58 (m, 1H), 8.47 (t, *J* = 5.6 Hz, 1H), 8.35 (s, 1H), 8.24 – 8.17 (m, 1H), 8.04 (d, *J* = 7.8 Hz, 1H), 7.96 (dt, *J* = 6.8, 2.4 Hz, 1H), 7.90 (td, *J* = 7.7, 1.8 Hz, 1H), 7.59 (s, 1H), 7.49 – 7.40 (m, 2H), 7.34 (ddd, *J* = 7.5, 4.7, 1.2 Hz, 1H), 3.28 (q, *J* = 6.6 Hz, 2H), 2.78 (s, 2H), 1.58 (dp, *J* = 10.7, 7.4 Hz, 4H), 1.38 (qd, *J* = 8.7, 6.1 Hz, 2H).
HRMS for $C_{20}H_{23}N_5OS$ $[M+H]^+$ calc.: 382.1696 Da; found: 382.1690 Da
Retention time Gradient A: 1.623 min

piperazin-1-yl(3-((4-(pyridin-2-yl)thiazol-2-yl)amino)phenyl)methanone (**CS-1-158**):

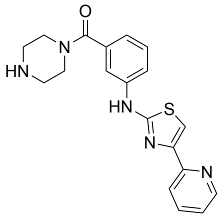

Was prepared according to **General Procedure A** starting from 3-((4-(pyridin-2-yl)thiazol-2-yl)amino)benzoic acid and *tert*-butyl piperazine-1-carboxylate. After purification of the Boc-protected intermediate, deprotection according to **General Procedure B** afforded **CS-1-158** as a yellow solid that was used without further purification. (114 mg, 62%) A small portion was purified via semipreparative HPLC for analytical purposes.

^{1}H NMR (500 MHz, DMSO-D_6) δ 10.52 (s, 1H), 8.92 (s, 2H), 8.62 (dd, *J* = 4.8, 1.6 Hz, 1H), 8.00 (d, *J* = 7.8 Hz, 1H), 7.95 (td, *J* = 7.7, 1.8 Hz, 1H), 7.89 (dd, *J* = 8.2, 2.3 Hz, 1H), 7.79 (d, *J* = 2.0 Hz, 1H), 7.63 (s, 1H), 7.47 (t, *J* = 7.9 Hz, 1H), 7.41 – 7.35 (m, 1H), 7.08 (d, *J* = 7.5 Hz, 1H), 3.19 (s, 4H). Proton signal for the piperazine CH_2-groups closer to the amide overlaps with H_2O signal.
HRMS for $C_{19}H_{19}N_5OS$ $[M+H]^+$ calc.: 366.1383 Da; found: 366.1376 Da
Retention time Gradient A: 1.539 min

N-(12-aminododecyl)-3-((4-(pyridin-2-yl)thiazol-2-yl)amino)benzamide (**CS-1-159**):

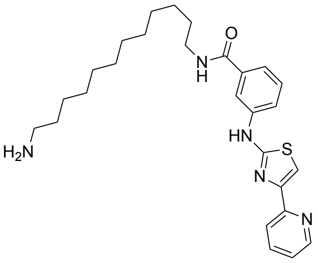

Was prepared according to **General Procedure A** starting from 3-((4-(pyridin-2-yl)thiazol-2-yl)amino)benzoic acid and *tert*-butyl (12-dodecyl)carbamate. After purification of the Boc-protected intermediate, deprotection according to **General Procedure B** afforded **CS-1-159** as a yellow solid that was used without further purification. (191 mg, 83%)

^{1}H NMR (400 MHz, TFA/$CDCl_3$/DMSO-D_6) δ 10.47 (s, 1H), 8.62 (d, *J* = 5.1 Hz, 1H), 8.31 (t, *J* = 5.6 Hz, 1H), 8.20 (p, *J* = 2.7 Hz, 1H), 8.11 – 8.04 (m, 2H), 7.94 (dt, *J* = 7.9, 1.8 Hz, 1H), 7.75 (s, 1H), 7.52 – 7.42 (m, 1H), 7.41 – 7.31 (m, 2H), 3.23 (q, *J* = 6.7 Hz, 2H), 2.71 (td, *J* = 7.7, 5.5 Hz, 2H), 1.49 (q, *J* = 7.4 Hz, 4H), 1.20 (d, *J* = 6.5 Hz, 16H).
HRMS for $C_{27}H_{37}N_5OS$ $[M+H]^+$ calc.: 480.2792 Da; found: 480.2783 Da
Retention time Gradient A: 2.031 min

N-(3-aminopropyl)-3-((4-(pyridin-2-yl)thiazol-2-yl)amino)benzamide (**CS-1-160**):

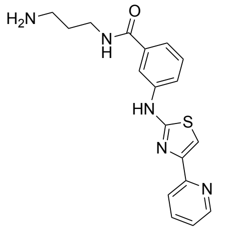

Was prepared according to **General Procedure A** starting from 3-((4-(pyridin-2-yl)thiazol-2-yl)amino)benzoic acid and *tert*-butyl (3-aminopropyl)carbamate. After purification of the Boc-protected intermediate, was deprotection according to **General Procedure B** afforded **CS-1-160** as a yellow solid that was used without further purification. (127 mg, 89%) A small portion was purified via semipreparative HPLC for analytical purposes.

^{1}H NMR (400 MHz, DMSO-D_6) δ 10.49 (s, 1H), 8.61 (t, *J* = 5.8 Hz, 1H), 8.59 – 8.52 (m, 1H), 8.23 (s, 1H), 8.19 (d, *J* = 2.4 Hz, 1H), 7.99 (d, *J* = 7.9 Hz, 1H), 7.89 (ddt, *J* = 17.4, 9.6, 5.3 Hz, 2H), 7.54 (s, 1H), 7.46 – 7.36 (m, 2H), 7.33 – 7.24 (m, 1H), 3.32 (q, *J* = 6.4 Hz, 2H), 2.82 (t, *J* = 7.4 Hz, 2H), 1.79 (p, *J* = 7.0 Hz, 2H).
HRMS for $C_{18}H_{19}N_5OS$ $[M+H]^+$ calc.: 354.1383 Da; found: 354.1377 Da
Retention time Gradient A: 1.579 min

(3-((4-(pyridin-2-yl)thiazol-2-yl)amino)phenyl)(3,9-diazaspiro[5.5]undecan-3-yl) methanone (**CS-1-161**):

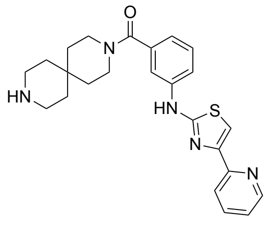

Was prepared according to **General Procedure A** starting from 3-((4-(pyridin-2-yl)thiazol-2-yl)amino)benzoic acid and *tert*-butyl (6-aminopropyl)carbamate. After purification of the Boc-protected intermediate, was deprotection according to **General Procedure B** afforded **CS-1-157** as a yellow solid that was used without further purification. (76 mg, 83%)

^{1}H NMR (400 MHz, DMSO-D_6) δ 10.54 (s, 1H), 8.64 – 8.47 (m, 1H), 8.35 (s, 1H), 7.92 (d, *J* = 7.8 Hz, 1H), 7.85 (td, *J* = 7.6, 1.8 Hz, 1H), 7.76 (d, *J* = 7.6 Hz, 2H), 7.54 (s, 1H), 7.38 (t, *J* = 7.8 Hz, 1H), 7.28 (ddd, *J* = 7.4, 4.7, 1.3 Hz, 1H), 6.92 (d, *J* = 7.5 Hz, 1H), 3.30 (s, 4H), 2.95 (s, 4H), 1.72 – 1.31 (m, 8H).
HRMS for $C_{24}H_{27}N_5OS$ $[M+H]^+$ calc.: 434.2009 Da; found: 434.2004 Da
Retention time Gradient A: 1.620 min

4-(3-(4-cyano-3-(trifluoromethyl)phenyl)-5,5-dimethyl-2,4-dioxoimidazolidin-1-yl)butanoic acid (**CS-1-154**):

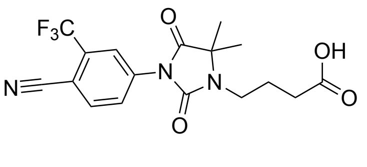

CS-1-54 was prepared according to a literature procedure reported by Gustafson et al.[11] After oxidation was complete, the reaction was diluted with 1 M HCl and extracted with Et_2O. Combined organic layers were dried over $MgSO_4$ filtered and evaporated. The compound obtained after lyophilization from water/MeCN as a white powder and used without further purification.

^{1}H NMR (400 MHz, METHANOL-D_4) δ 8.17 (d, *J* = 2.1 Hz, 1H), 8.11 – 7.98 (m, 2H), 3.43 (dd, *J* = 8.6, 6.4 Hz, 2H), 2.40 (t, *J* = 7.2 Hz, 2H), 2.06 – 1.91 (m, 2H), 1.56 – 1.47 (m, 6H).
HRMS for $C_{17}H_{17}F_3N_3O_4$ $[M+H]^+$ calc.: 384.1166 Da; found: 384.1159 Da

4-(3-(4-cyano-3-(trifluoromethyl)phenyl)-5,5-dimethyl-4-oxo-2-thioxoimidazolidin-1-yl) butanoic acid (**CS-1-170**)

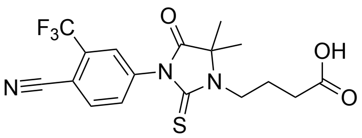

CS-1-170 was prepared analogously to **CS-1-154** and obtained as an amorphous solid.

^{1}H NMR (400 MHz, METHANOL-D_4) δ 8.14 – 7.98 (m, 2H), 7.87 (dd, J = 8.3, 2.2 Hz, 1H), 3.85 – 3.71 (m, 2H), 2.40 (t, J = 7.2 Hz, 2H), 2.20 – 2.02 (m, 1H), 1.56 (s, 6H).
HRMS for $C_{17}H_{17}F_3N_3O_3S$ $[M+H]^+$ calc.: 400.0937 Da; found: 400.0927 Da

Supplementary References

(1) Millard, C. J.; Fairall, L.; Ragan, T. J.; Savva, C. G.; Schwabe, J. W. R. The Topology of Chromatin-Binding Domains in the NuRD Deacetylase Complex. *Nucleic Acids Res.* **2020**, *48* (22), 12972–12982. https://doi.org/10.1093/nar/gkaa1121.

(2) Becker, T.; Wiest, A.; Telek, A.; Bejko, D.; Hoffmann-Röder, A.; Kielkowski, P. Transforming Chemical Proteomics Enrichment into a High-Throughput Method Using an SP2E Workflow. *JACS Au* **2022**, *2* (7), 1712–1723. https://doi.org/10.1021/jacsau.2c00284.

(3) Bray, N. L.; Pimentel, H.; Melsted, P.; Pachter, L. Near-Optimal Probabilistic RNA-Seq Quantification. *Nat. Biotechnol.* **2016**, *34* (5), 525–527. https://doi.org/10.1038/nbt.3519.

(4) Love, M. I.; Huber, W.; Anders, S. Moderated Estimation of Fold Change and Dispersion for RNA-Seq Data with DESeq2. *Genome Biol.* **2014**, *15* (12), 550. https://doi.org/10.1186/s13059-014-0550-8.

(5) Korotkevich, G.; Sukhov, V.; Budin, N.; Shpak, B.; Artyomov, M. N.; Sergushichev, A. Fast Gene Set Enrichment Analysis. bioRxiv February 1, 2021, p 060012. https://doi.org/10.1101/060012.

(6) Liberzon, A.; Birger, C.; Thorvaldsdóttir, H.; Ghandi, M.; Mesirov, J. P.; Tamayo, P. The Molecular Signatures Database Hallmark Gene Set Collection. *Cell Syst.* **2015**, *1* (6), 417–425. https://doi.org/10.1016/j.cels.2015.12.004.

(7) Chen, E. Y.; Tan, C. M.; Kou, Y.; Duan, Q.; Wang, Z.; Meirelles, G. V.; Clark, N. R.; Ma’ayan, A. Enrichr: Interactive and Collaborative HTML5 Gene List Enrichment Analysis Tool. *BMC Bioinformatics* **2013**, *14* (1), 128. https://doi.org/10.1186/1471-2105-14-128.

(8) Keenan, A. B.; Torre, D.; Lachmann, A.; Leong, A. K.; Wojciechowicz, M. L.; Utti, V.; Jagodnik, K. M.; Kropiwnicki, E.; Wang, Z.; Ma’ayan, A. ChEA3: Transcription Factor Enrichment Analysis by Orthogonal Omics Integration. *Nucleic Acids Res.* **2019**, *47* (W1), W212–W224. https://doi.org/10.1093/nar/gkz446.

(9) Ting, P. Y.; Borikar, S.; Kerrigan, J. R.; Thomsen, N. M.; Aghania, E.; Hinman, A. E.; Reyes, A.; Pizzato, N.; Fodor, B. D.; Wu, F.; Belew, M. S.; Mao, X.; Wang, J.; Chitnis, S.; Niu, W.; Hachey, A.; Cobb, J. S.; Savage, N. A.; Burke, A.; Paulk, J.; Dovala, D.; Lin, J.; Clifton, M. C.; Ornelas, E.; Ma, X.; Ware, N. F.; Sanchez, C. C.; Taraszka, J.; Terranova, R.; Knehr, J.; Altorfer, M.; Barnes, S. W.; Beckwith, R. E. J.; Solomon, J. M.; Dales, N. A.; Patterson, A. W.; Wagner, J.; Bouwmeester, T.; Dranoff, G.; Stevenson, S. C.; Bradner, J. E. A Molecular Glue Degrader of the WIZ Transcription Factor for Fetal Hemoglobin Induction. *Science* **2024**, *385* (6704), 91–99. https://doi.org/10.1126/science.adk6129.

(10) Skene, P. J.; Henikoff, J. G.; Henikoff, S. Targeted in Situ Genome-Wide Profiling with High Efficiency for Low Cell Numbers. *Nat. Protoc.* **2018**, *13* (5), 1006–1019. https://doi.org/10.1038/nprot.2018.015.

(11) Gustafson, J. L.; Neklesa, T. K.; Cox, C. S.; Roth, A. G.; Buckley, D. L.; Tae, H. S.; Sundberg, T. B.; Stagg, D. B.; Hines, J.; McDonnell, D. P.; Norris, J. D.; Crews, C. M. Small-Molecule-Mediated Degradation of the Androgen Receptor through Hydrophobic Tagging. *Angew. Chem. Int. Ed.* **2015**, *54* (33), 9659–9662. https://doi.org/10.1002/anie.201503720.

CS-1-69:

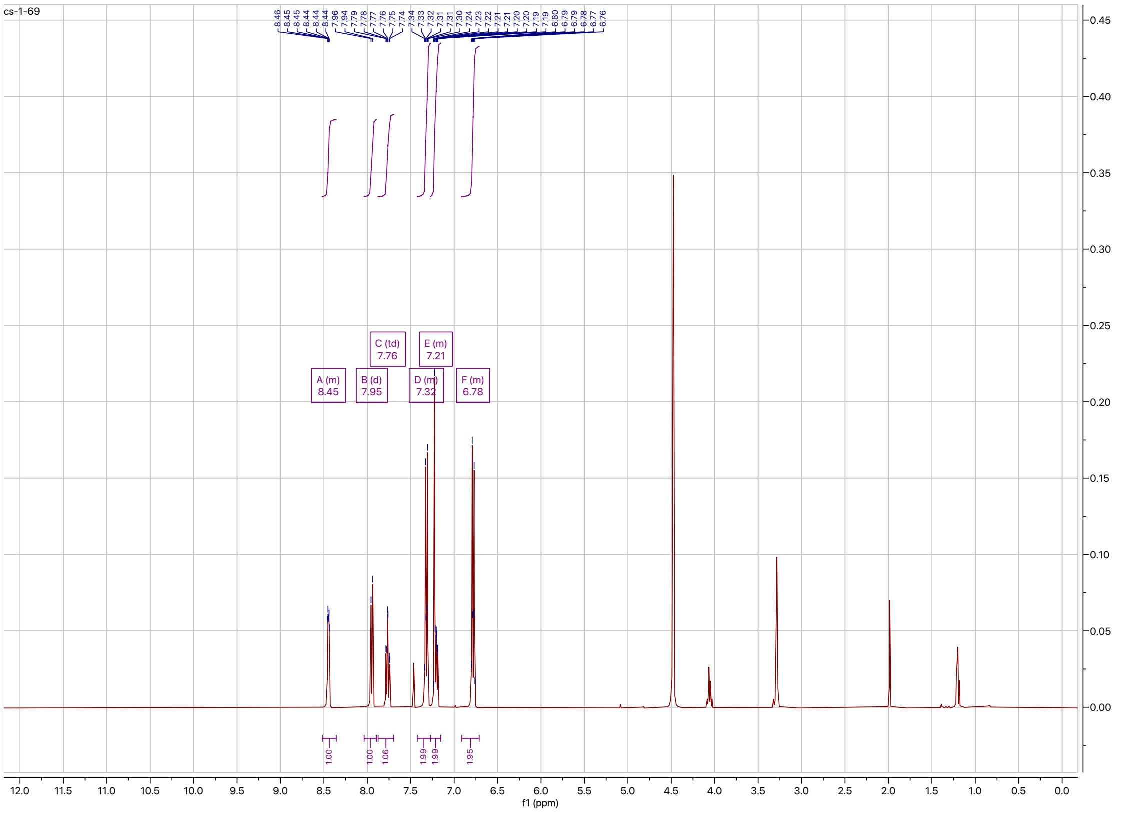
cs-1-69
A (m) 8.45
B (d) 7.95
C (td) 7.76
D (m) 7.32
E (m) 7.21
F (m) 6.78
f1 (ppm)

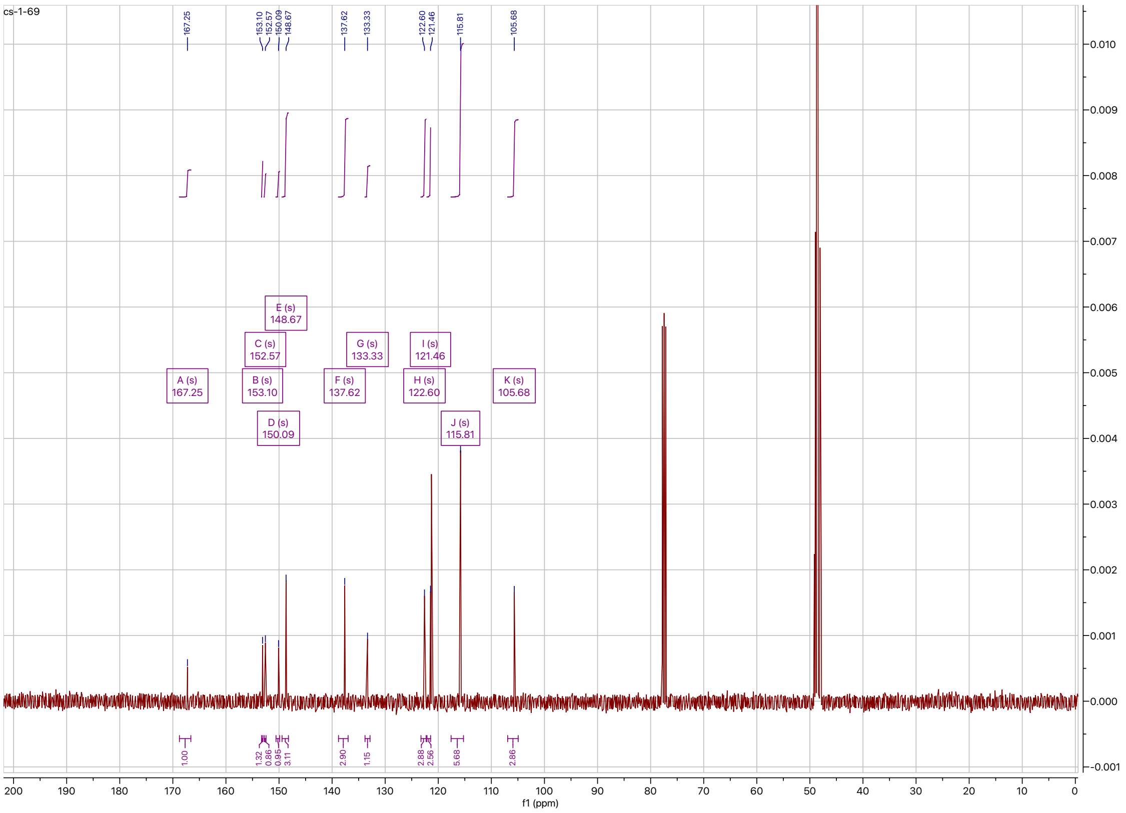
cs-1-69
A (s) 167.25
B (s) 153.10
C (s) 152.57
D (s) 150.09
E (s) 148.67
F (s) 137.62
G (s) 133.33
H (s) 122.60
I (s) 121.46
J (s) 115.81
K (s) 105.68
f1 (ppm)

CS-1-71:

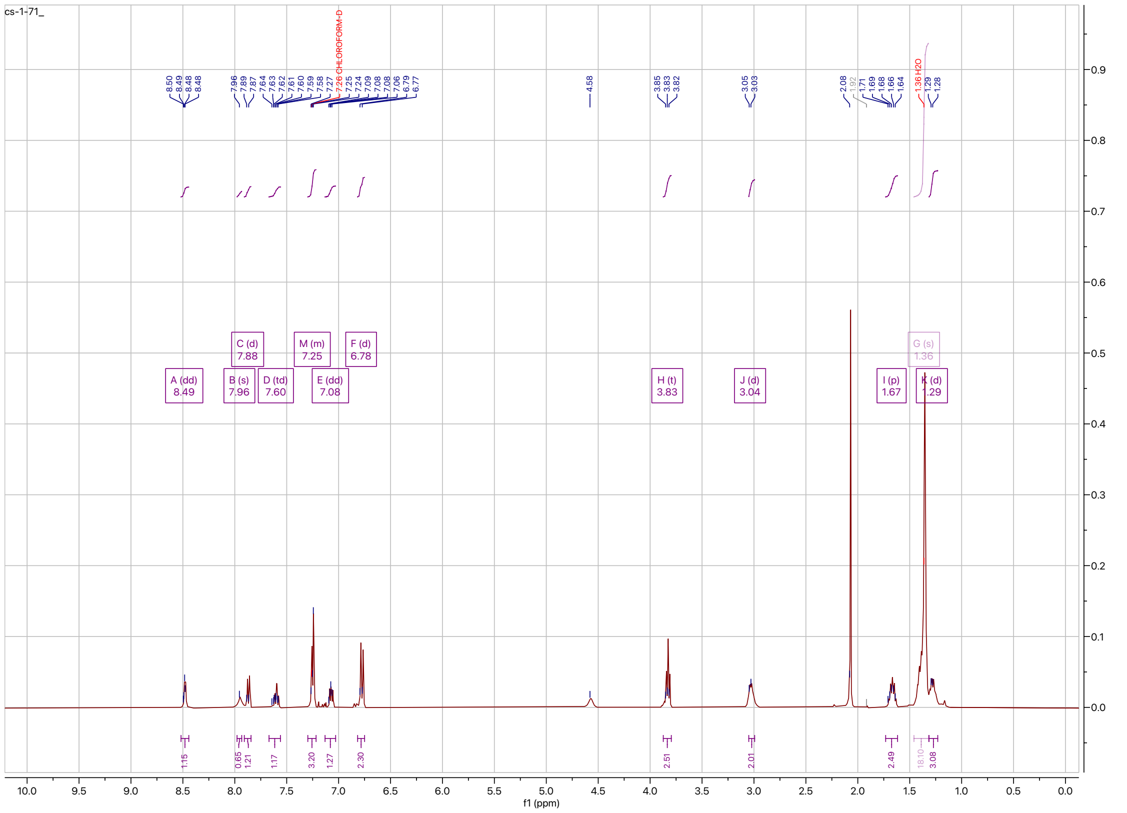

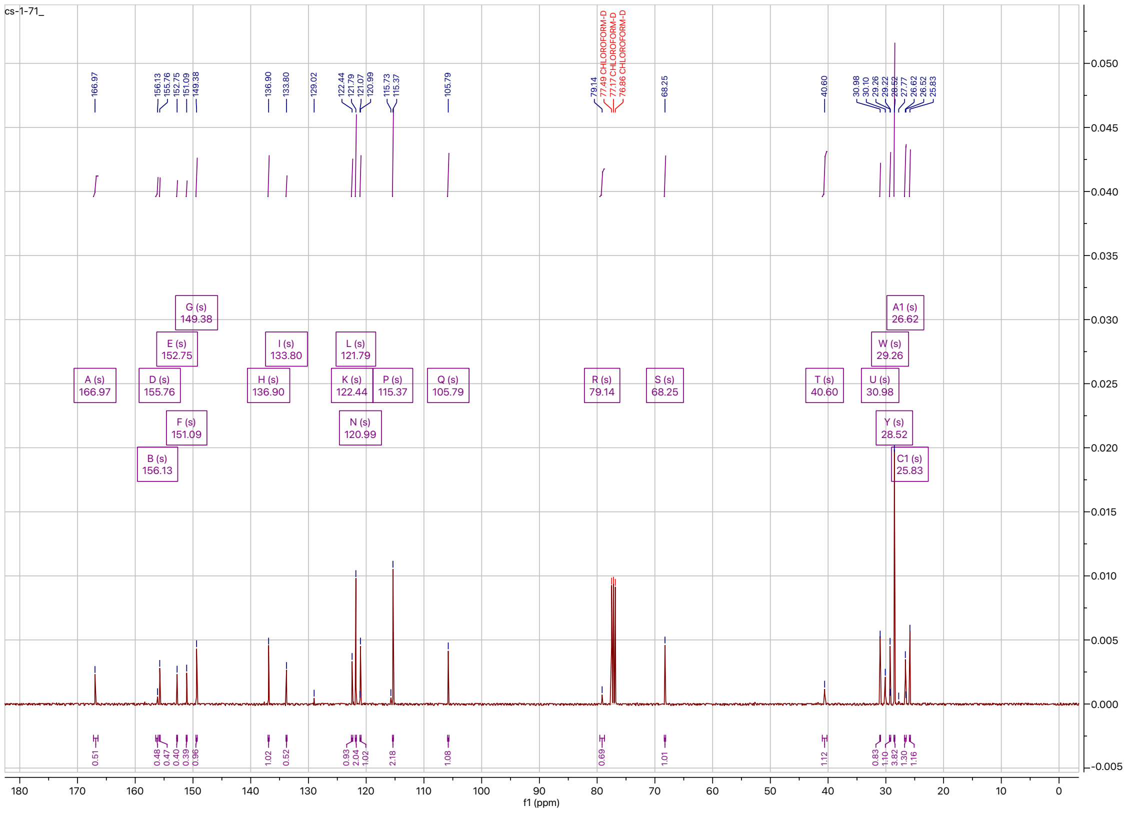

CS-1-76:

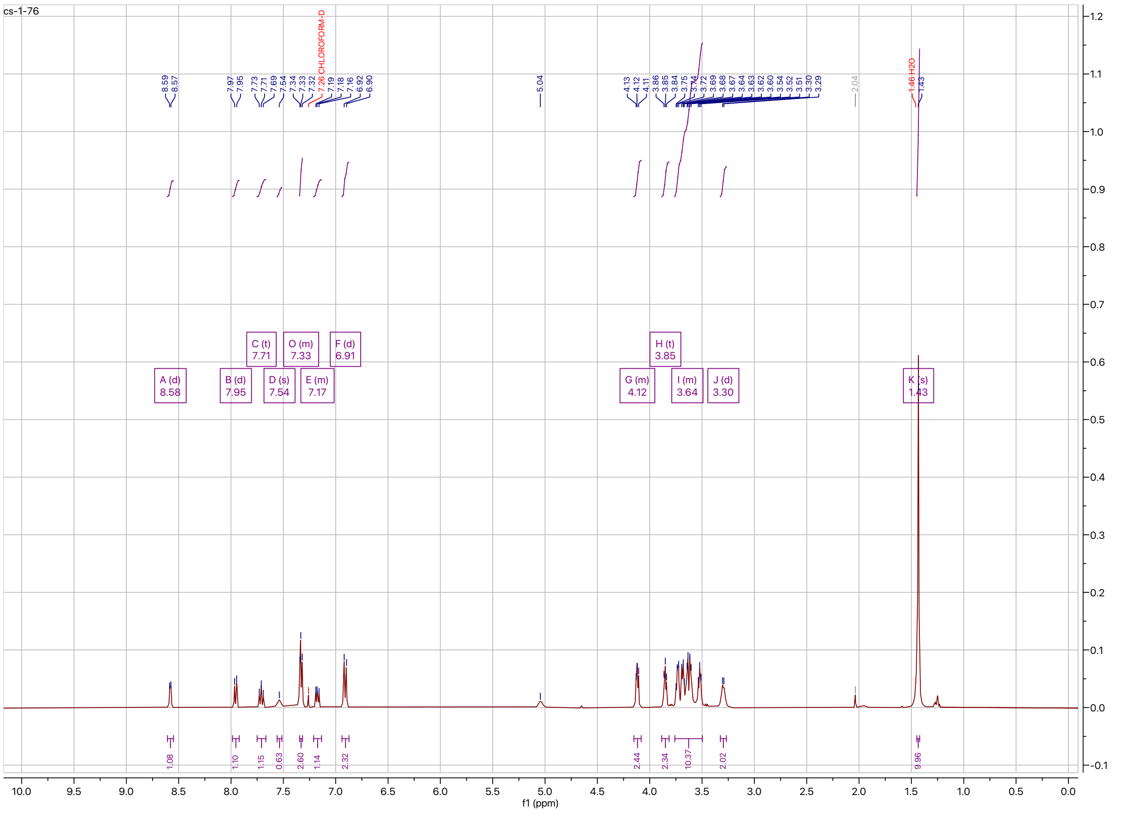

cs-1-76
8.59
8.57
7.97
7.95
7.73
7.71
7.69
7.54
7.34
7.33
7.32
7.26 CHLOROFORM-D
7.19
7.18
7.16
6.92
6.90
5.04
4.13
4.12
4.11
3.86
3.85
3.84
3.75
3.74
3.72
3.69
3.68
3.67
3.64
3.63
3.62
3.60
3.54
3.52
3.51
3.30
3.29
2.04
1.46 H2O
1.43
C (t) 7.71
O (m) 7.33
F (d) 6.91
H (t) 3.85
A (d) 8.58
B (d) 7.95
D (s) 7.54
E (m) 7.17
G (m) 4.12
I (m) 3.64
J (d) 3.30
K (s) 1.43
1.08
1.10
1.15
0.63
2.60
1.14
2.32
2.44
2.34
10.37
2.02
9.96
f1 (ppm)

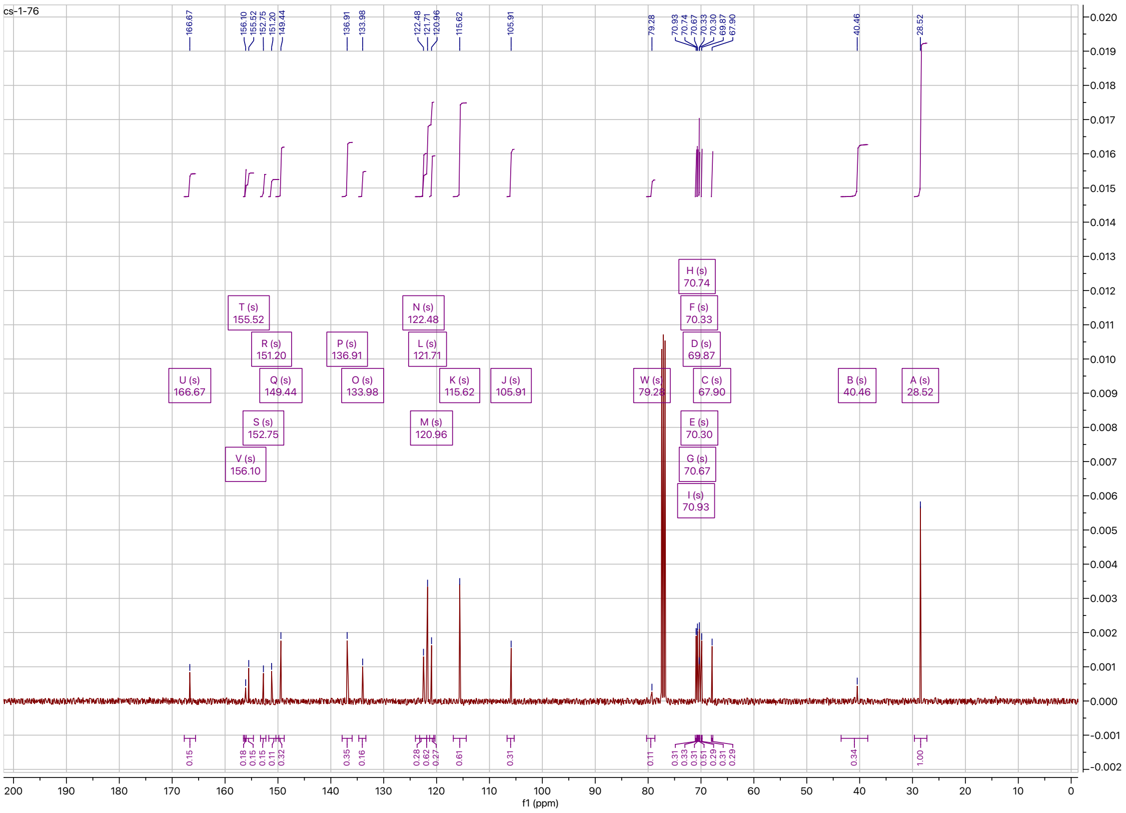

cs-1-76
166.67
156.10
155.52
152.75
151.20
149.44
136.91
133.98
122.48
121.71
120.96
115.62
105.91
79.28
70.93
70.74
70.67
70.33
70.30
69.87
67.90
40.46
28.52
H (s) 70.74
T (s) 155.52
N (s) 122.48
F (s) 70.33
R (s) 151.20
P (s) 136.91
L (s) 121.71
D (s) 69.87
U (s) 166.67
Q (s) 149.44
O (s) 133.98
K (s) 115.62
J (s) 105.91
W (s) 79.28
C (s) 67.90
B (s) 40.46
A (s) 28.52
S (s) 152.75
M (s) 120.96
E (s) 70.30
V (s) 156.10
G (s) 70.67
I (s) 70.93
f1 (ppm)

CS-1-85:

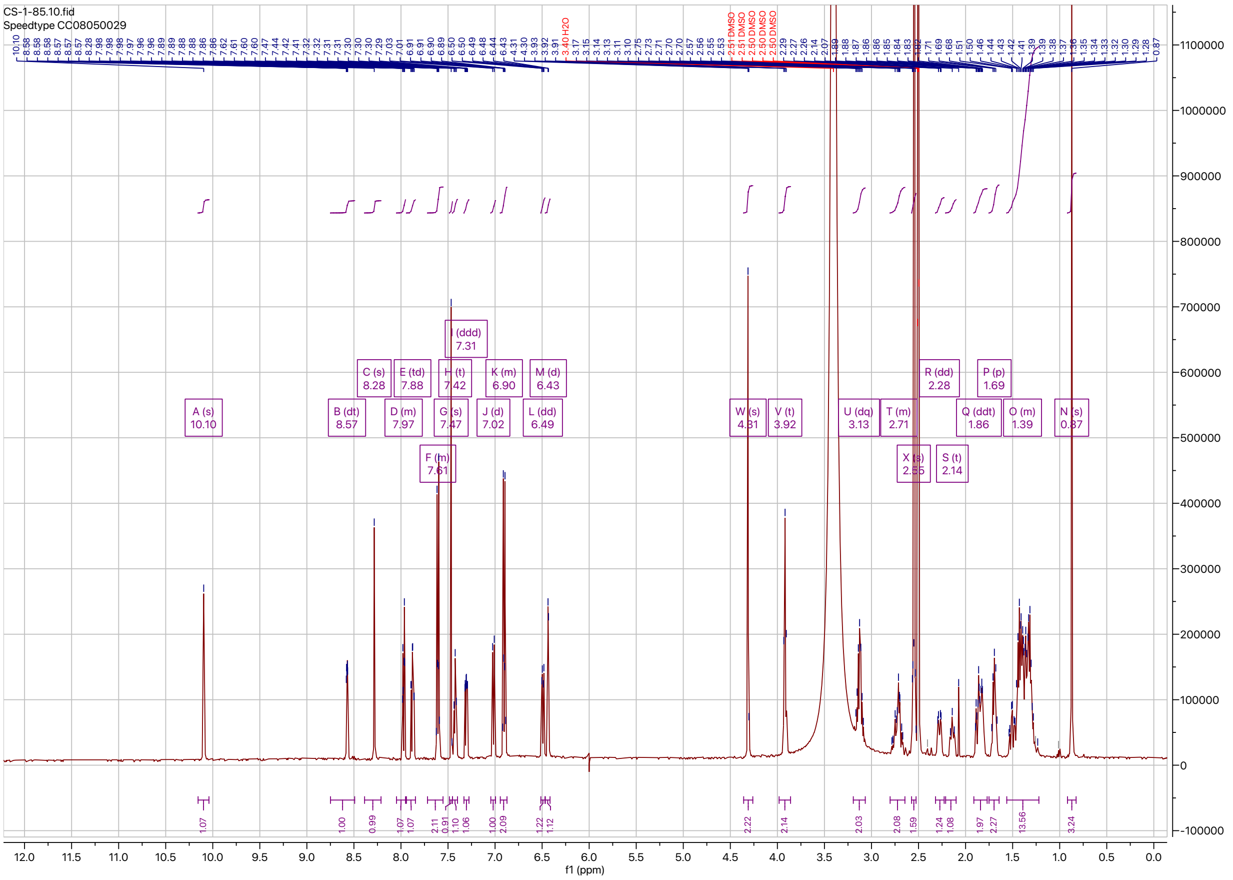

CS-1-85.10.fid
Speedtype CC08050029
A (s) 10.10
B (dt) 8.57
C (s) 8.28
D (m) 7.97
E (td) 7.88
F (m) 7.61
G (s) 7.47
H (t) 7.42
I (ddd) 7.31
J (d) 7.02
K (m) 6.90
L (dd) 6.49
M (d) 6.43
W (s) 4.31
V (t) 3.92
U (dq) 3.13
T (m) 2.71
X (s) 2.55
R (dd) 2.28
S (t) 2.14
Q (ddt) 1.86
P (p) 1.69
O (m) 1.39
N (s) 0.87
f1 (ppm)

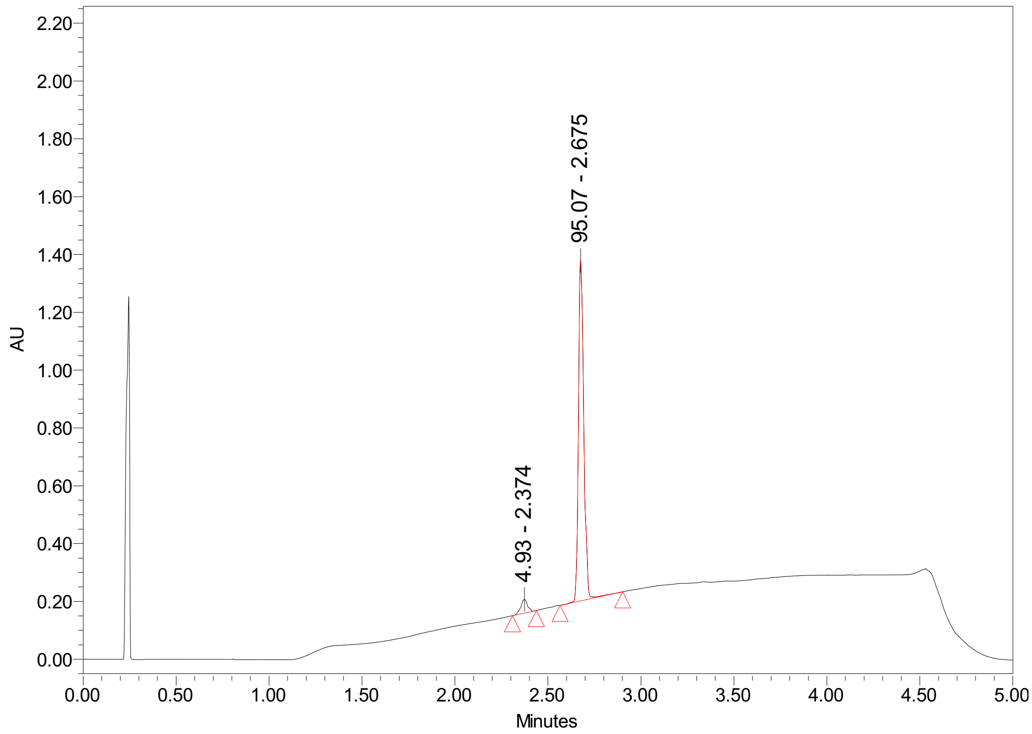

AU
Minutes
4.93 - 2.374
95.07 - 2.675

CS-1-86:

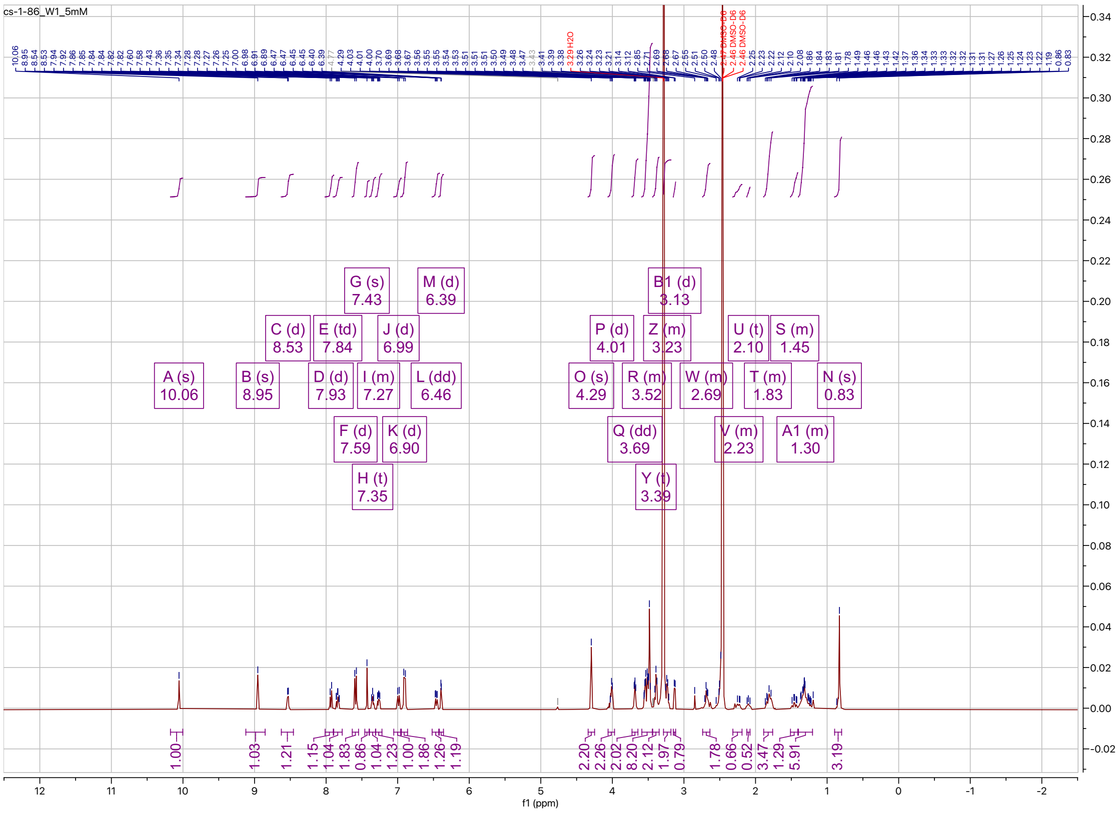
cs-1-86_W1_5mM
A (s) 10.06
B (s) 8.95
C (d) 8.53
D (d) 7.93
E (td) 7.84
F (d) 7.59
G (s) 7.43
H (t) 7.35
I (m) 7.27
J (d) 6.99
K (d) 6.90
L (dd) 6.46
M (d) 6.39
O (s) 4.29
P (d) 4.01
Q (dd) 3.69
R (m) 3.52
Y (t) 3.39
Z (m) 3.23
B1 (d) 3.13
W (m) 2.69
V (m) 2.23
U (t) 2.10
T (m) 1.83
S (m) 1.45
A1 (m) 1.30
N (s) 0.83
f1 (ppm)

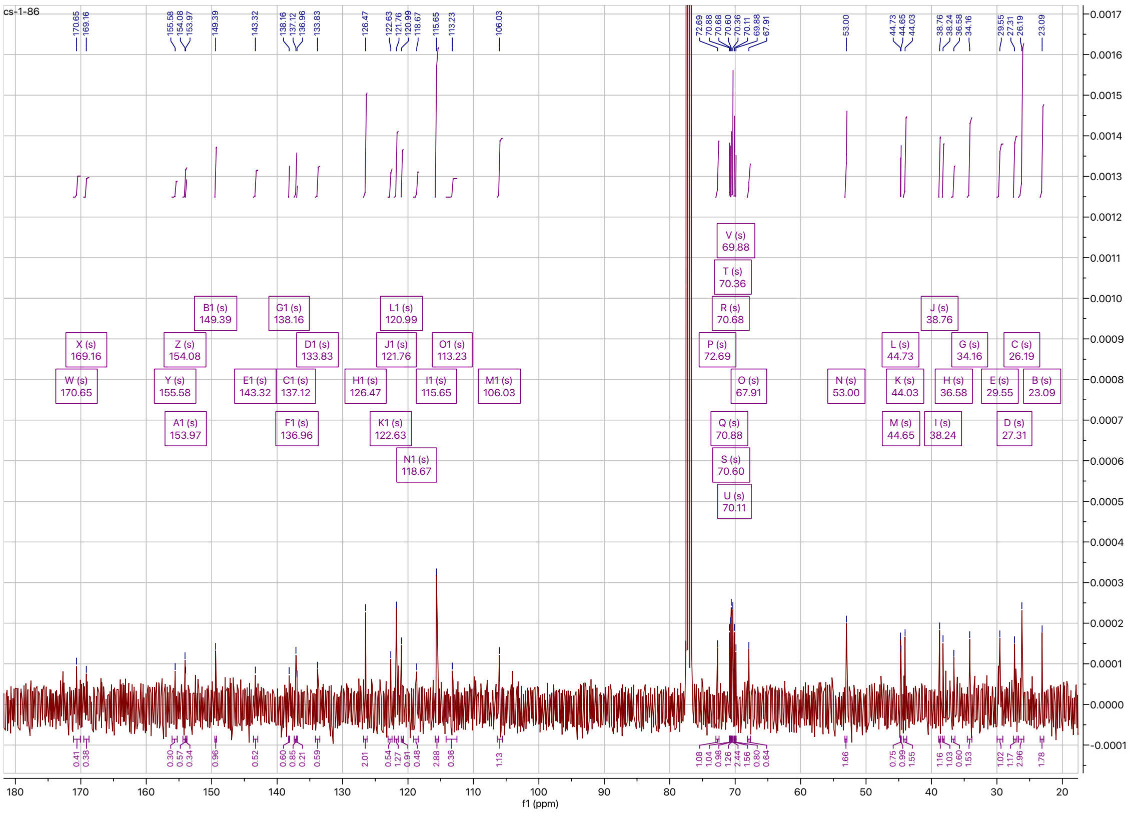
cs-1-86
W (s) 170.65
X (s) 169.16
Y (s) 155.58
Z (s) 154.08
A1 (s) 153.97
B1 (s) 149.39
E1 (s) 143.32
G1 (s) 138.16
C1 (s) 137.12
F1 (s) 136.96
D1 (s) 133.83
H1 (s) 126.47
K1 (s) 122.63
J1 (s) 121.76
L1 (s) 120.99
N1 (s) 118.67
I1 (s) 115.65
O1 (s) 113.23
M1 (s) 106.03
P (s) 72.69
Q (s) 70.88
R (s) 70.68
S (s) 70.60
T (s) 70.36
U (s) 70.11
V (s) 69.88
O (s) 67.91
N (s) 53.00
L (s) 44.73
M (s) 44.65
K (s) 44.03
J (s) 38.76
I (s) 38.24
H (s) 36.58
G (s) 34.16
E (s) 29.55
D (s) 27.31
C (s) 26.19
B (s) 23.09
f1 (ppm)

CS-1-99:

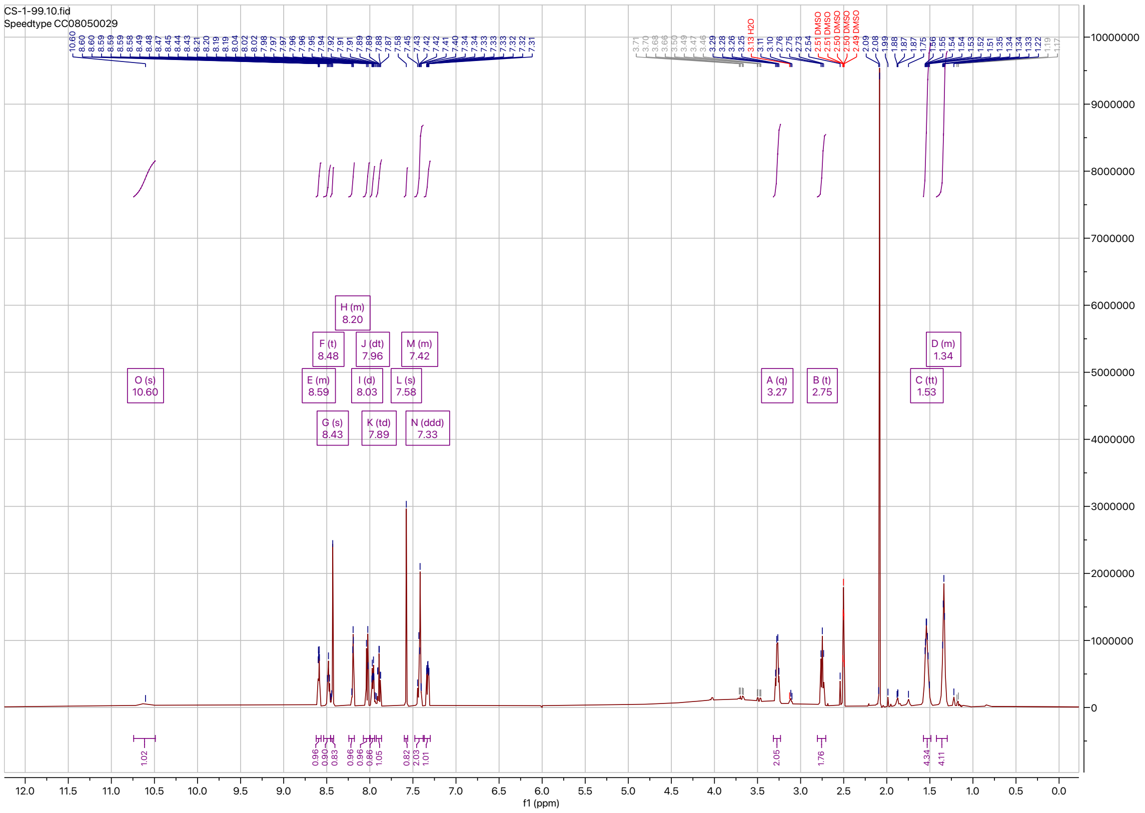

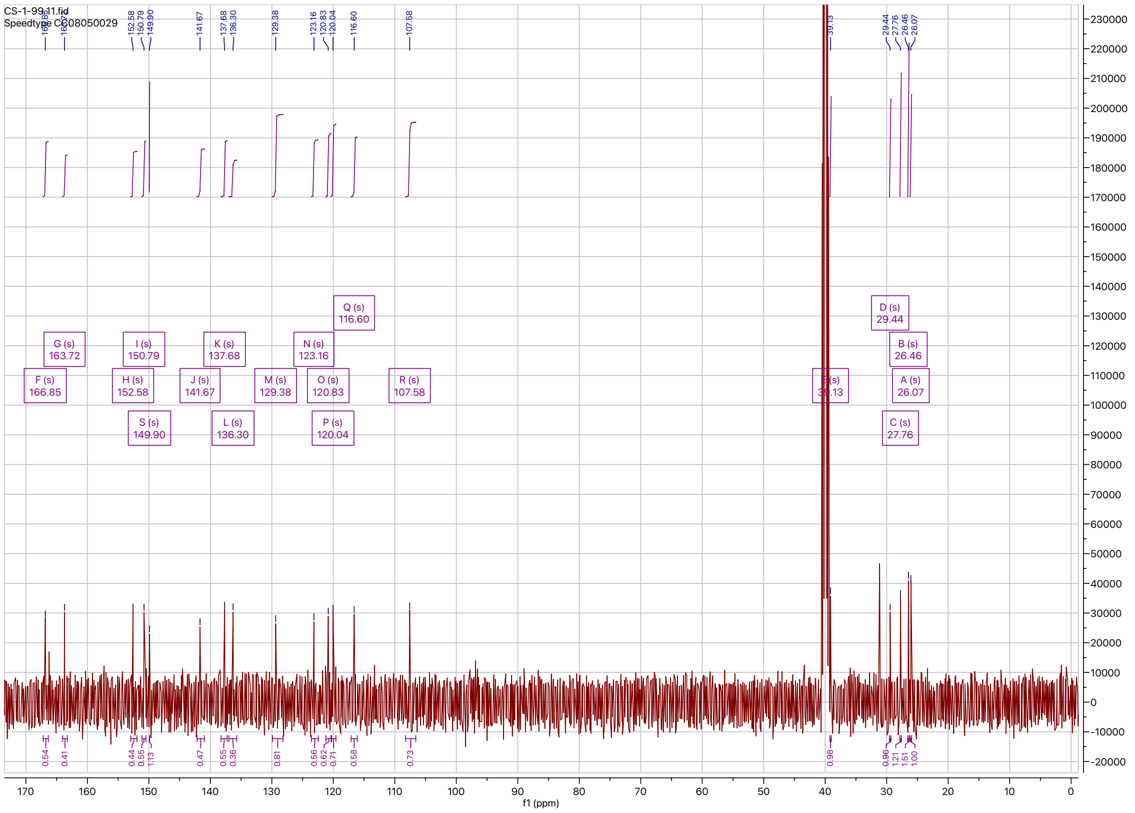

CS-1-102:

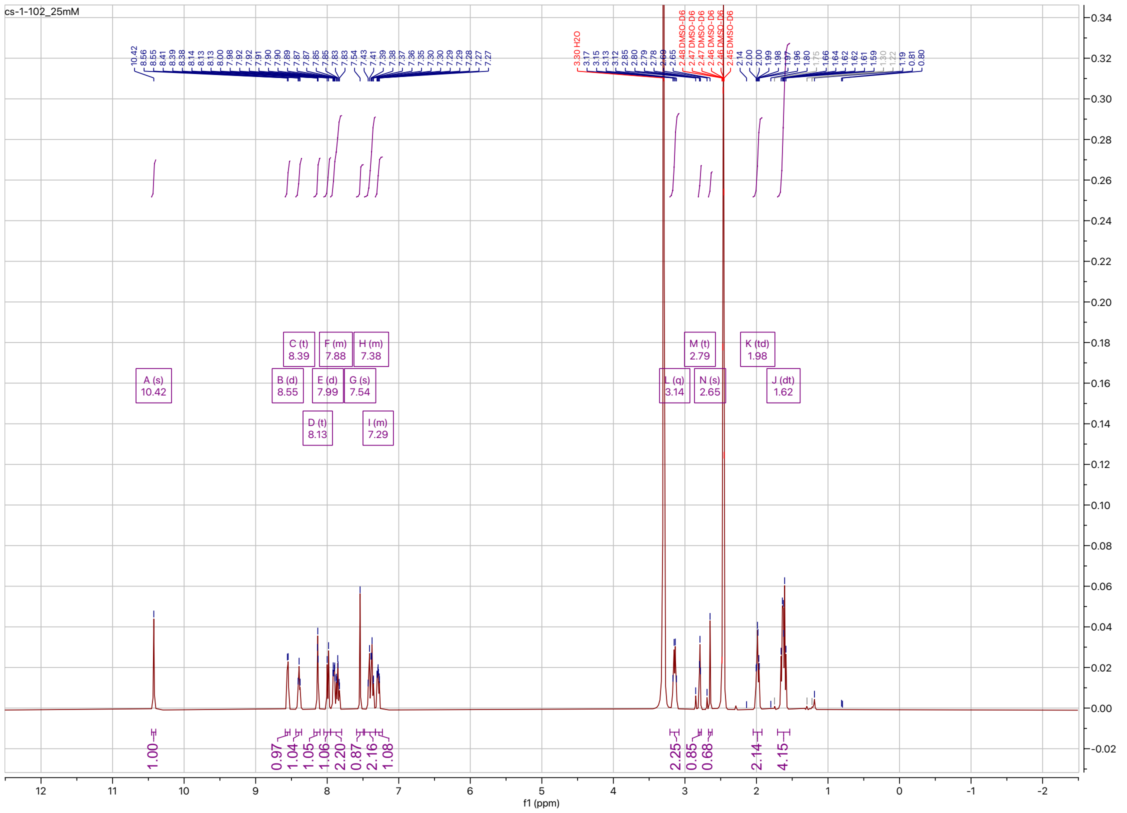

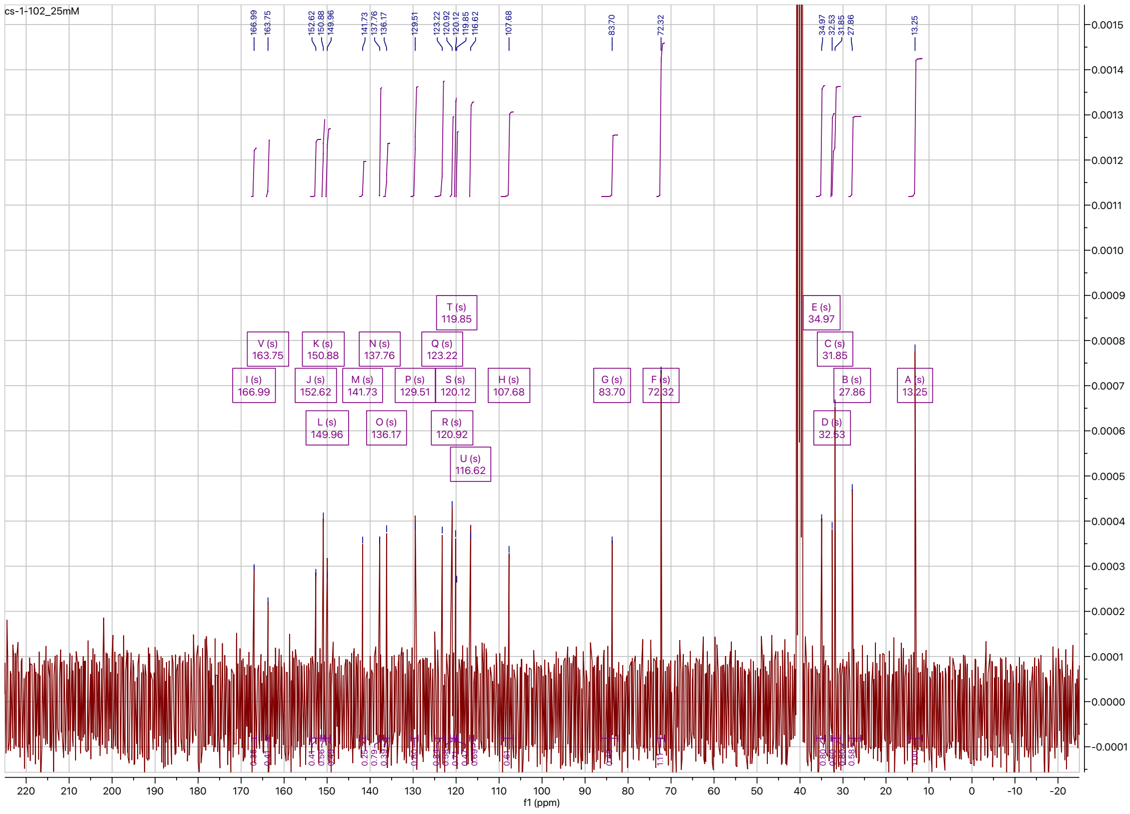

CS-1-103:

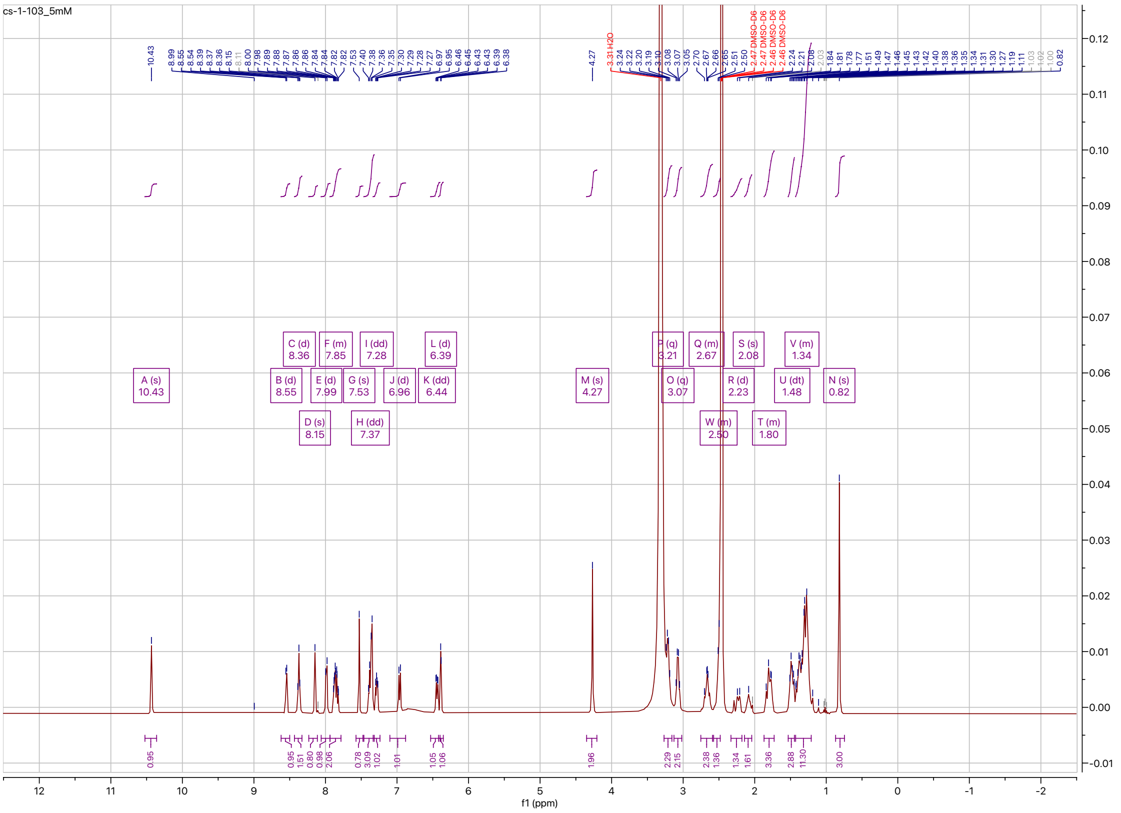

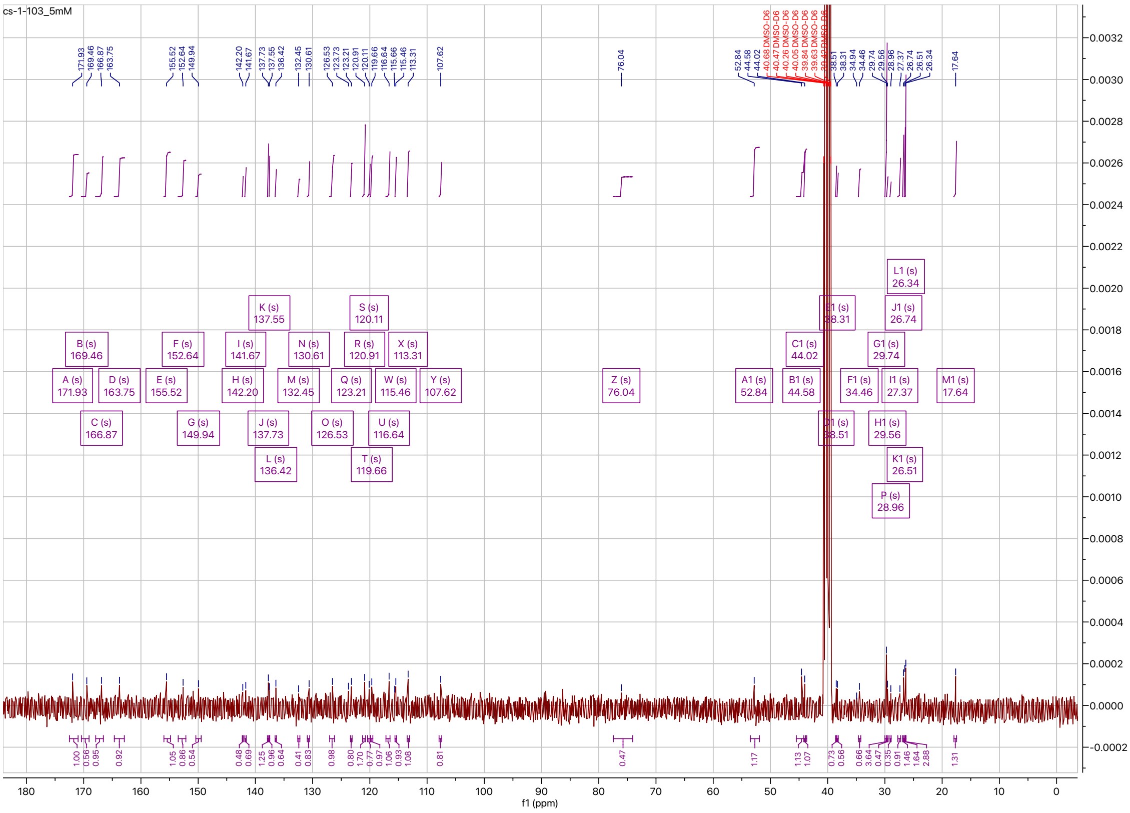

CS-1-123:

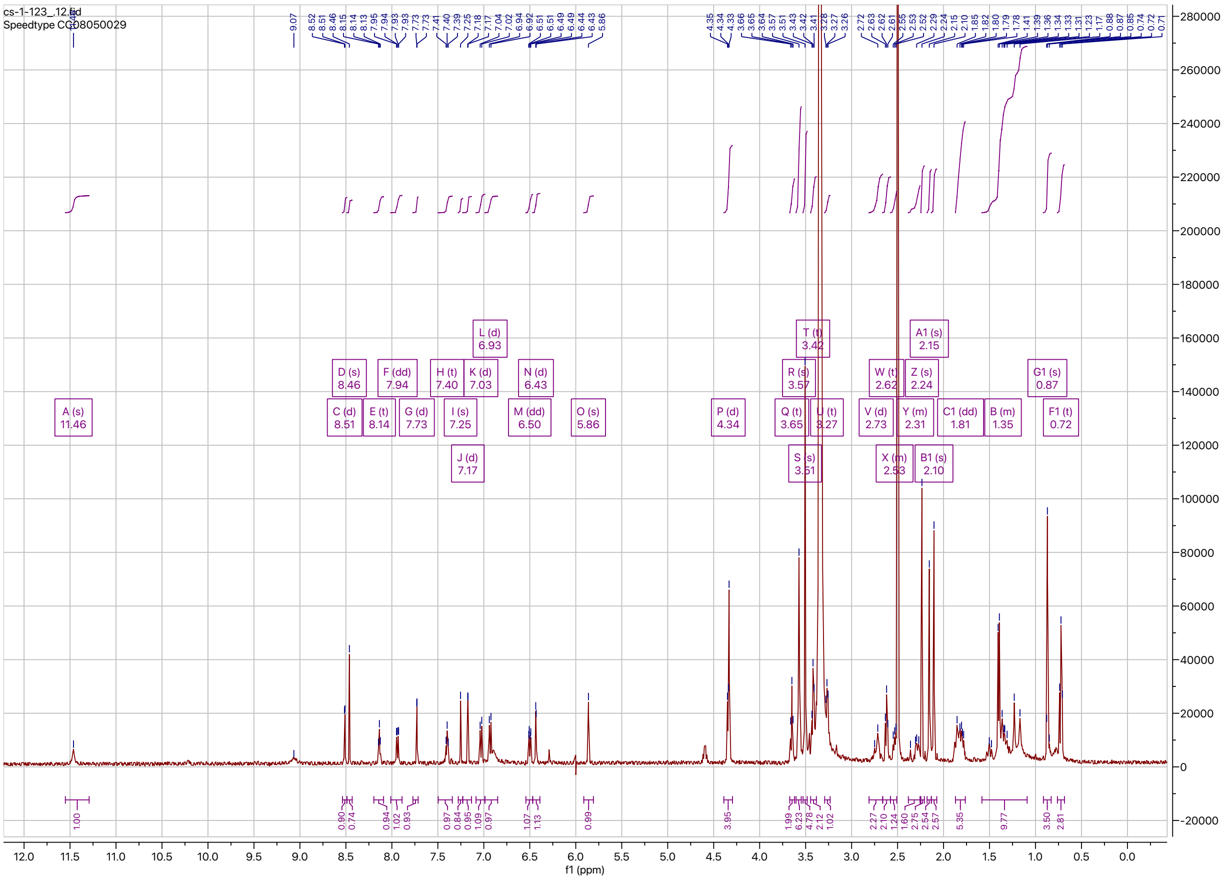
cs-1-123_.12.fid
Speedtype CG08050029
A (s)
11.46
C (d)
8.51
D (s)
8.46
E (t)
8.14
F (dd)
7.94
G (d)
7.73
H (t)
7.40
I (s)
7.25
J (d)
7.17
K (d)
7.03
L (d)
6.93
M (dd)
6.50
N (d)
6.43
O (s)
5.86
P (d)
4.34
Q (t)
3.65
R (s)
3.57
S (s)
3.51
T (t)
3.42
U (t)
3.27
V (d)
2.73
W (t)
2.62
X (m)
2.53
Y (m)
2.31
Z (s)
2.24
A1 (s)
2.15
B1 (s)
2.10
C1 (dd)
1.81
B (m)
1.35
G1 (s)
0.87
F1 (t)
0.72
f1 (ppm)

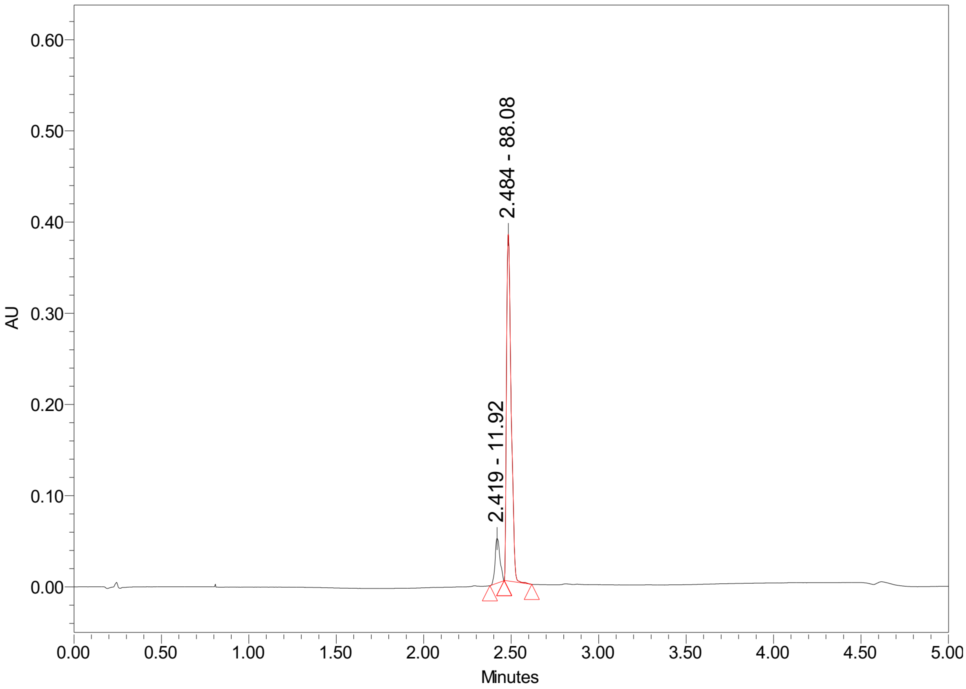
2.484 - 88.08
2.419 - 11.92
AU
Minutes

CS-1-132:

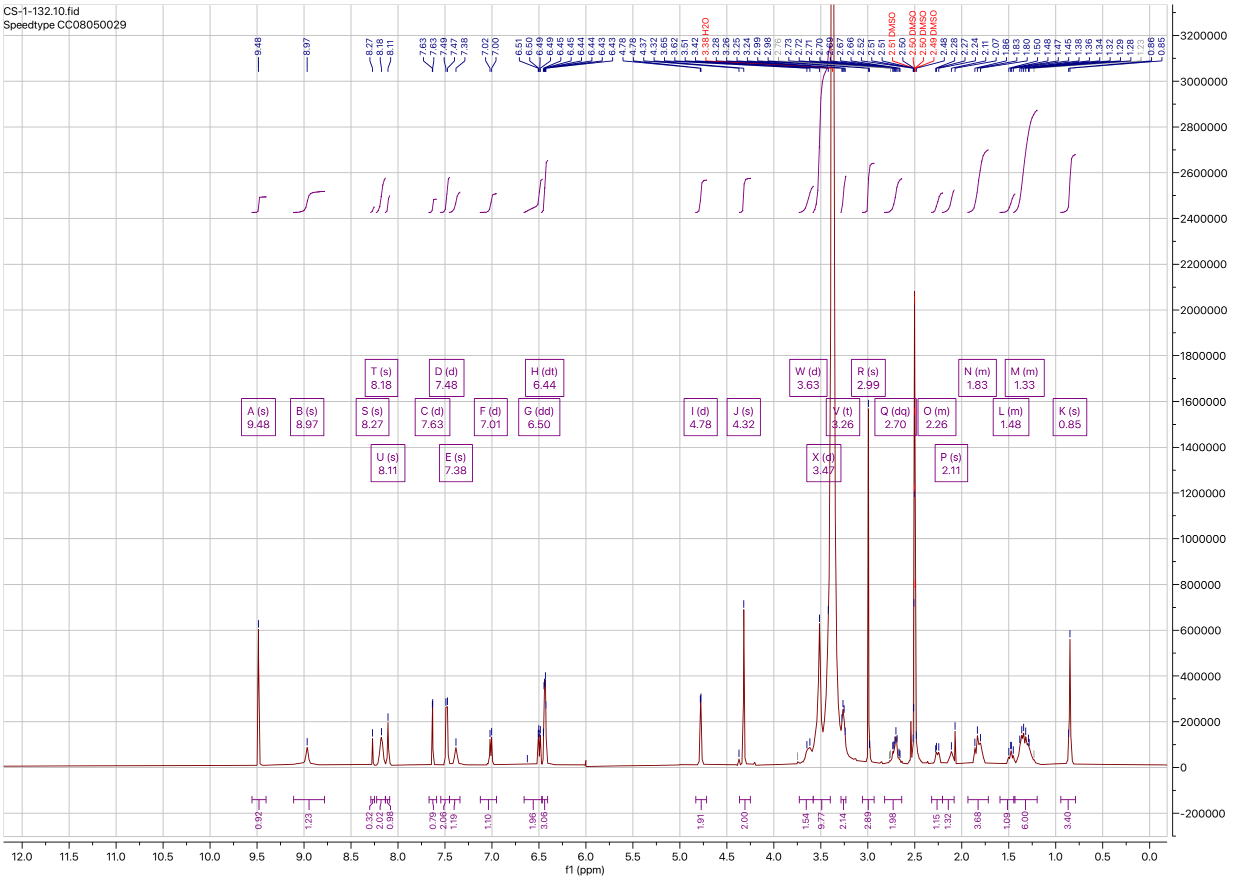

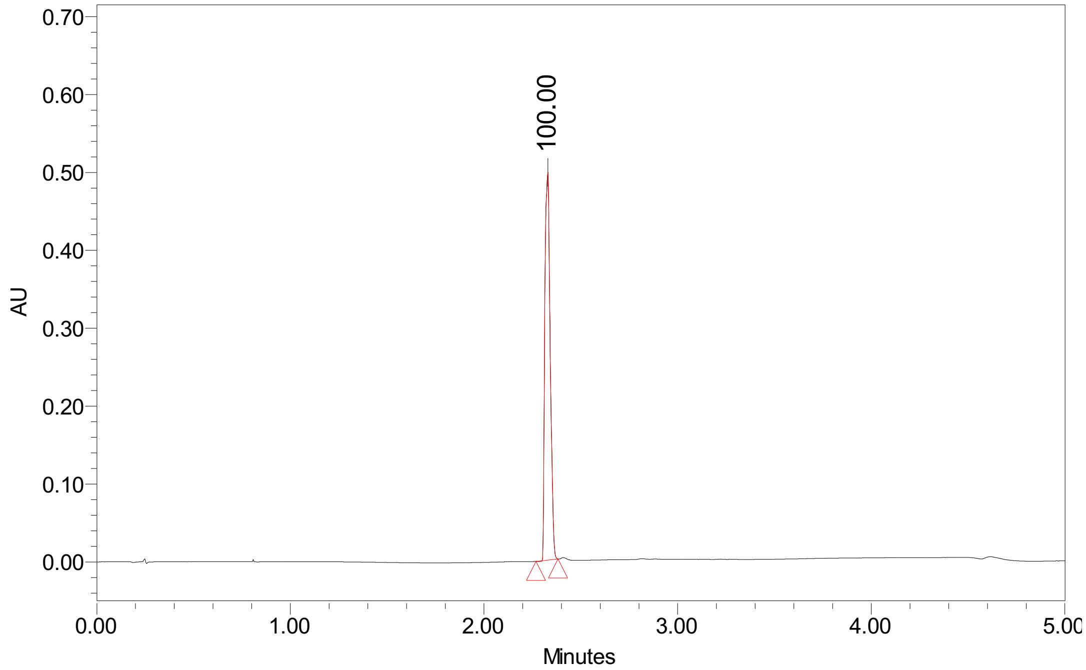

CS-1-157:

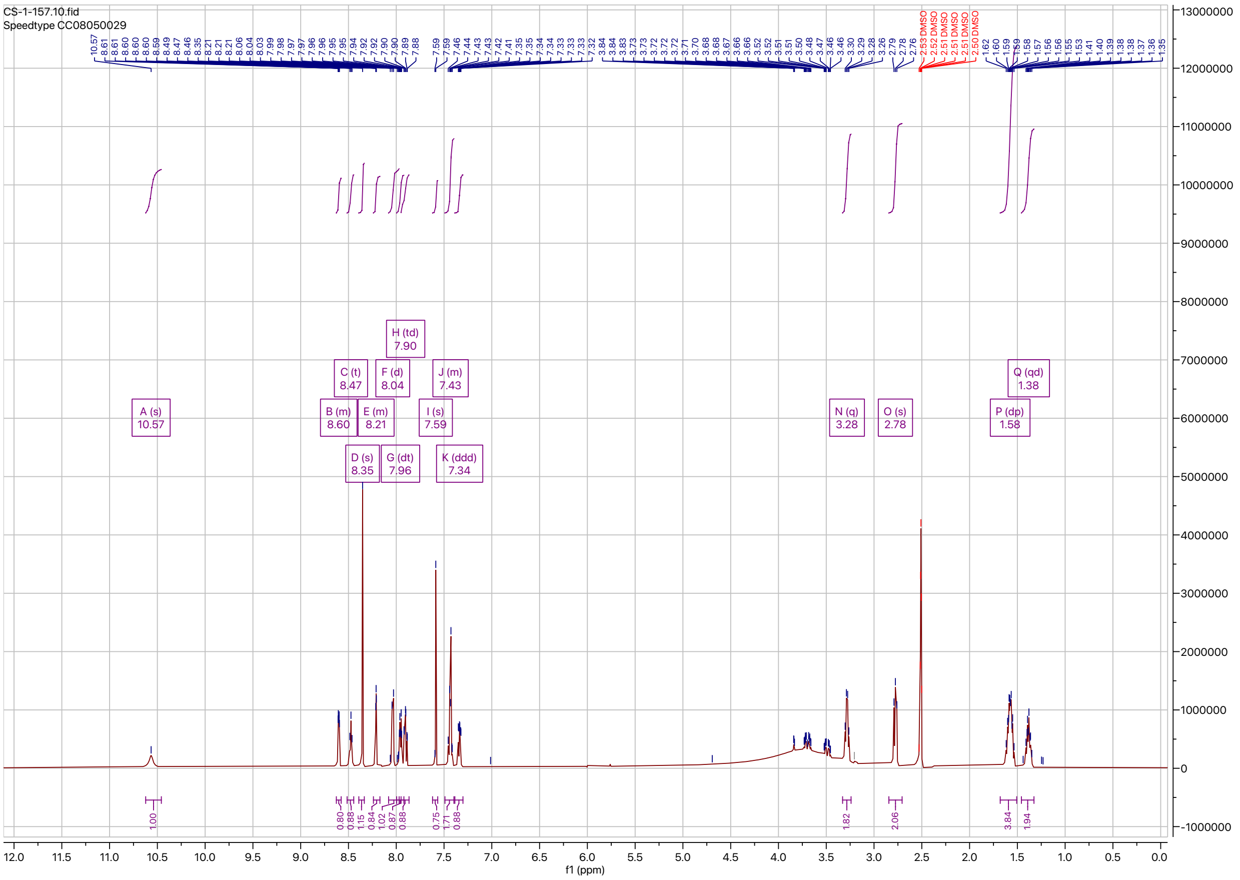

CS-1-157.10.fid
Speedtype CC08050029
A (s) 10.57
B (m) 8.60
C (t) 8.47
D (s) 8.35
E (m) 8.21
F (d) 8.04
G (dt) 7.96
H (td) 7.90
I (s) 7.59
J (m) 7.43
K (ddd) 7.34
N (q) 3.28
O (s) 2.78
P (dp) 1.58
Q (qd) 1.38
f1 (ppm)

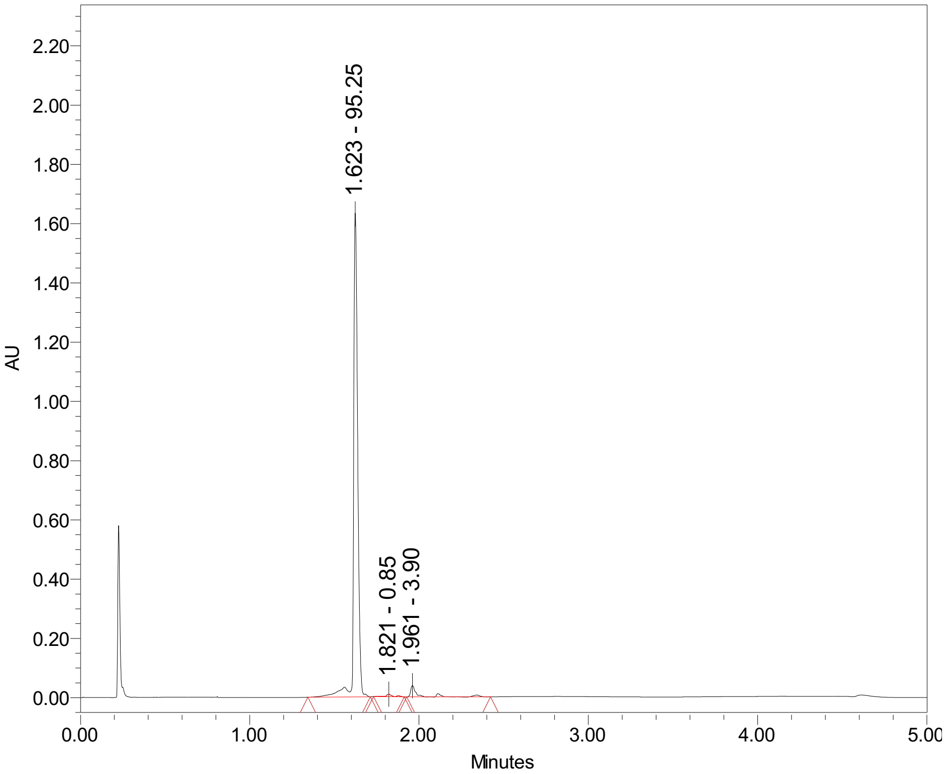

1.623 - 95.25
1.821 - 0.85
1.961 - 3.90
AU
2.20
2.00
1.80
1.60
1.40
1.20
1.00
0.80
0.60
0.40
0.20
0.00
0.00
1.00
2.00
3.00
4.00
5.00
Minutes

CS-1-158:

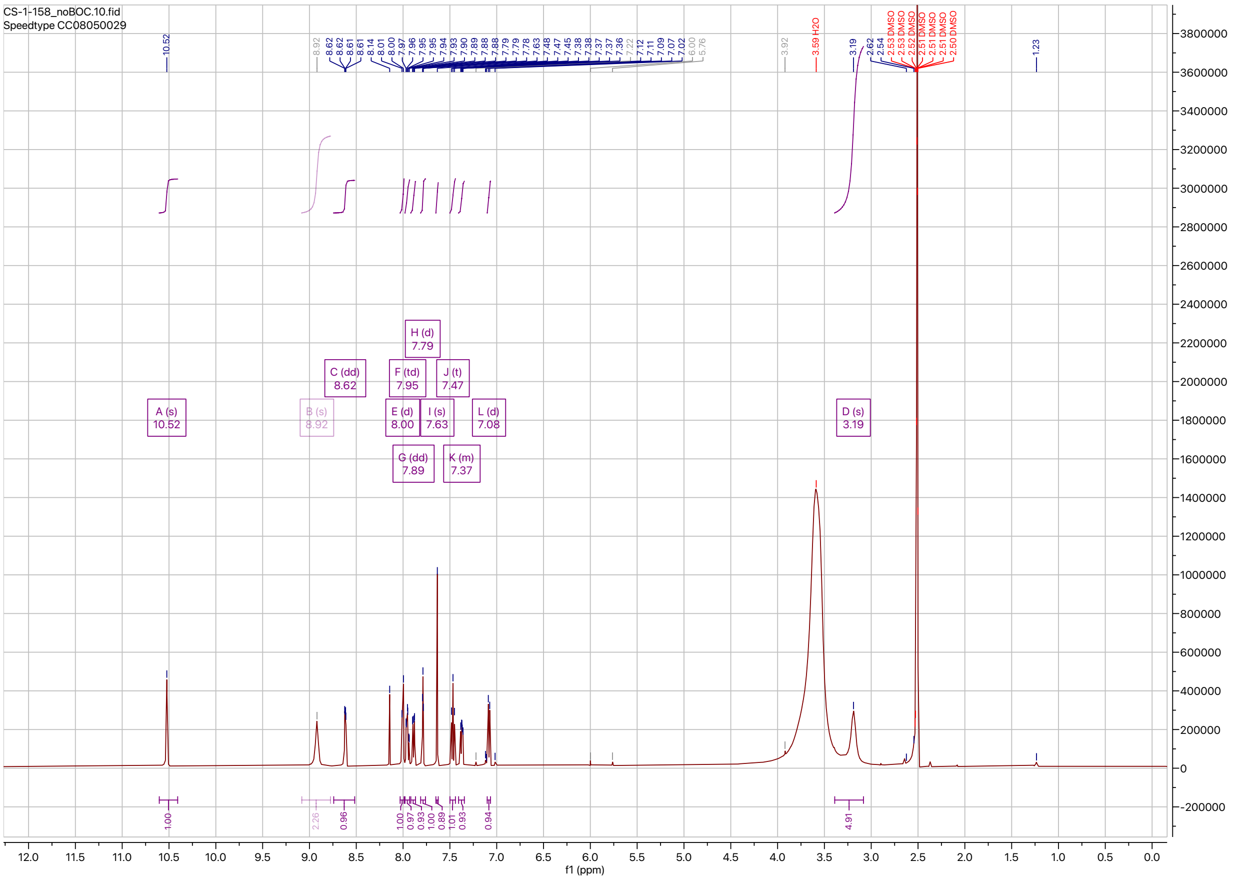
CS-1-158_noBOC.10.fid
Speedtype CC08050029
A (s)
10.52
B (s)
8.92
C (dd)
8.62
E (d)
8.00
F (td)
7.95
G (dd)
7.89
H (d)
7.79
I (s)
7.63
J (t)
7.47
K (m)
7.37
L (d)
7.08
D (s)
3.19
3.59 H2O
f1 (ppm)

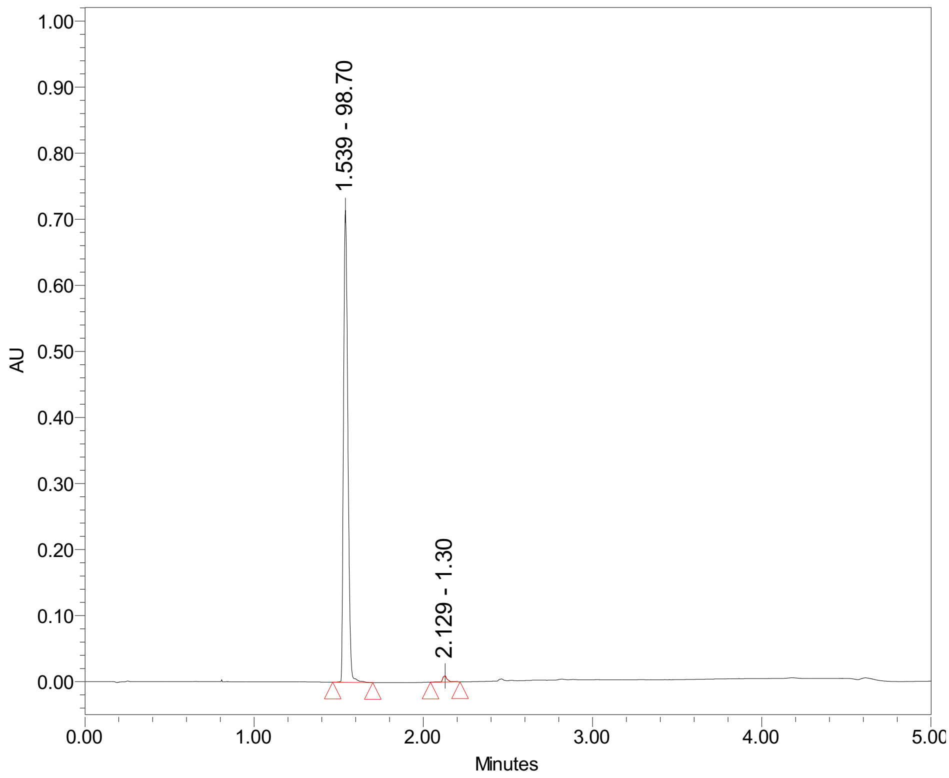
1.539 - 98.70
2.129 - 1.30
AU
Minutes

CS-1-159:

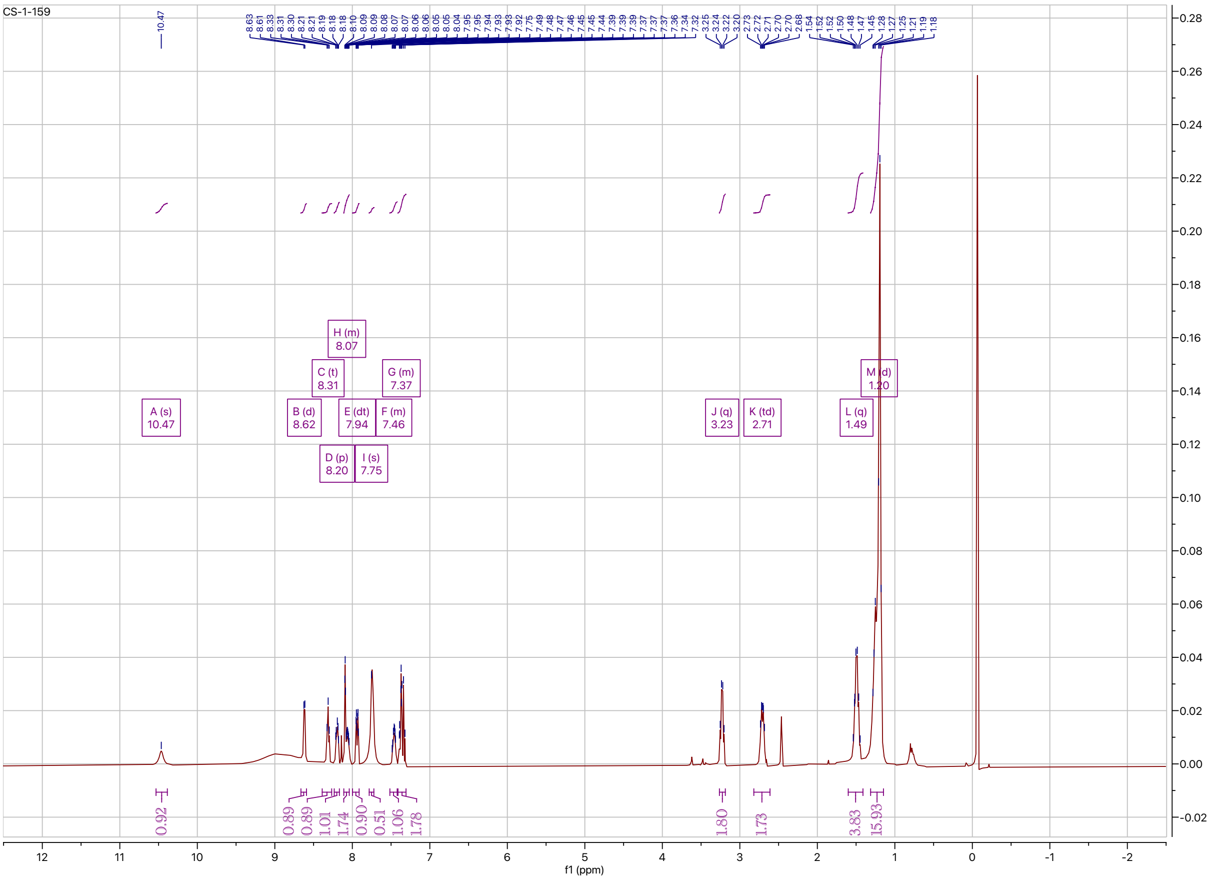
CS-1-159
A (s)
10.47
B (d)
8.62
C (t)
8.31
D (p)
8.20
E (dt)
7.94
F (m)
7.46
G (m)
7.37
H (m)
8.07
I (s)
7.75
J (q)
3.23
K (td)
2.71
L (q)
1.49
M (d)
1.20
f1 (ppm)

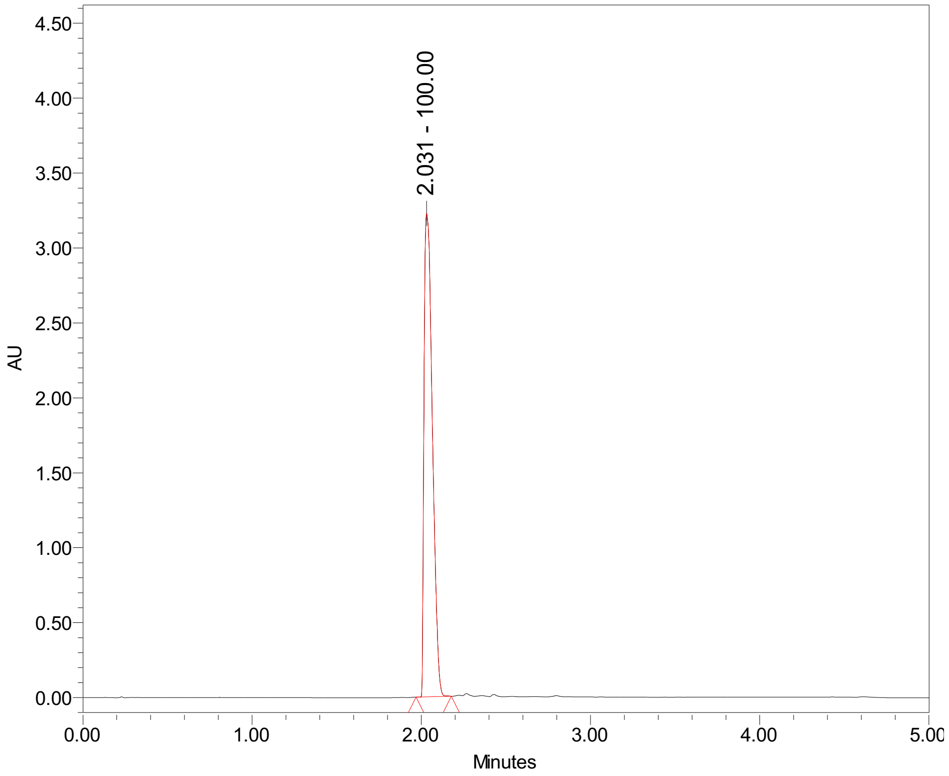
2.031 - 100.00
AU
Minutes

CS-1-160:

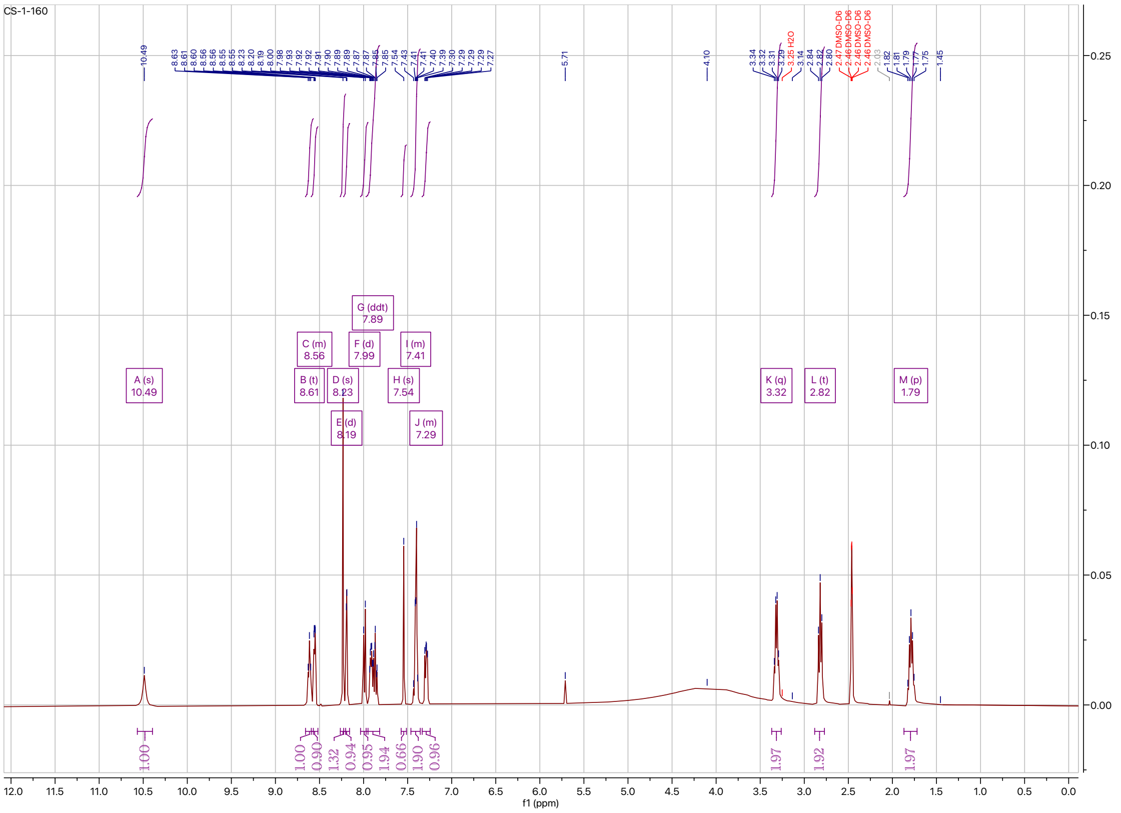

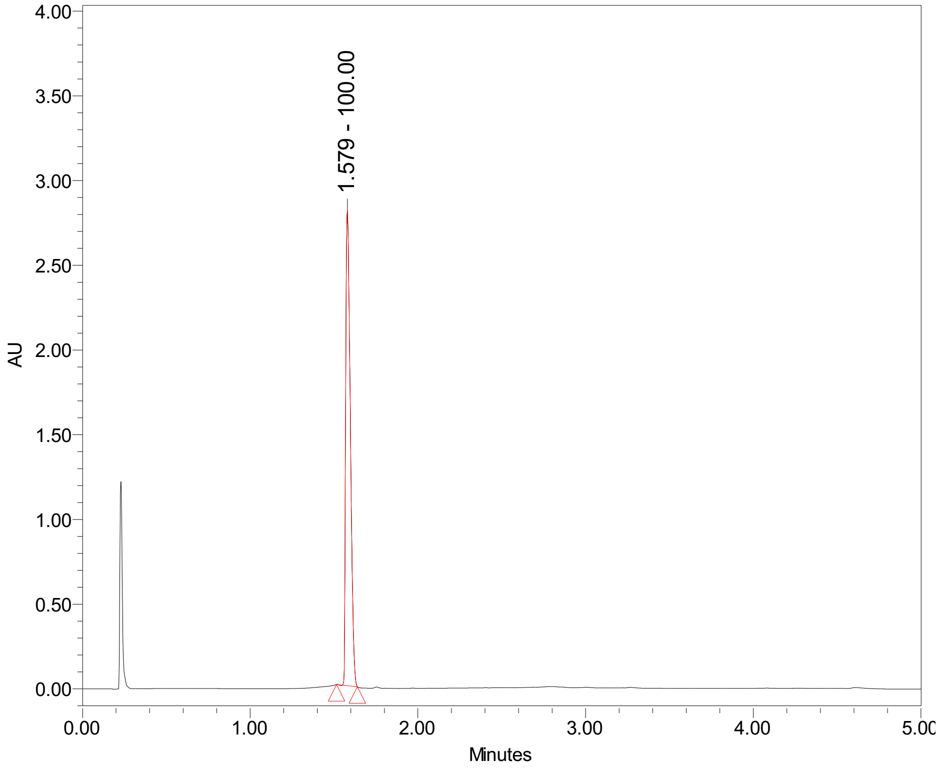

CS-1-161:

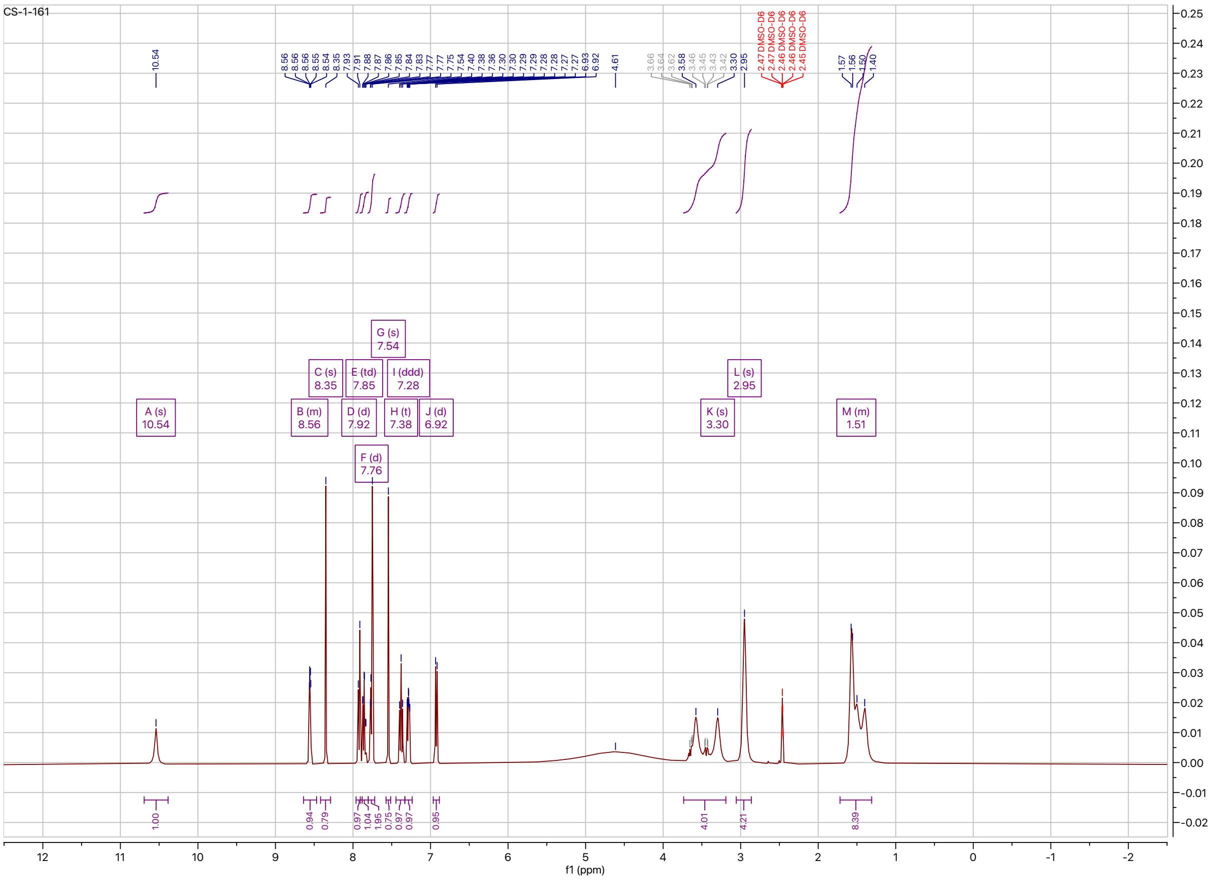
CS-1-161
A (s) 10.54
B (m) 8.56
C (s) 8.35
D (d) 7.92
E (td) 7.85
F (d) 7.76
G (s) 7.54
H (t) 7.38
I (ddd) 7.28
J (d) 6.92
K (s) 3.30
L (s) 2.95
M (m) 1.51
f1 (ppm)

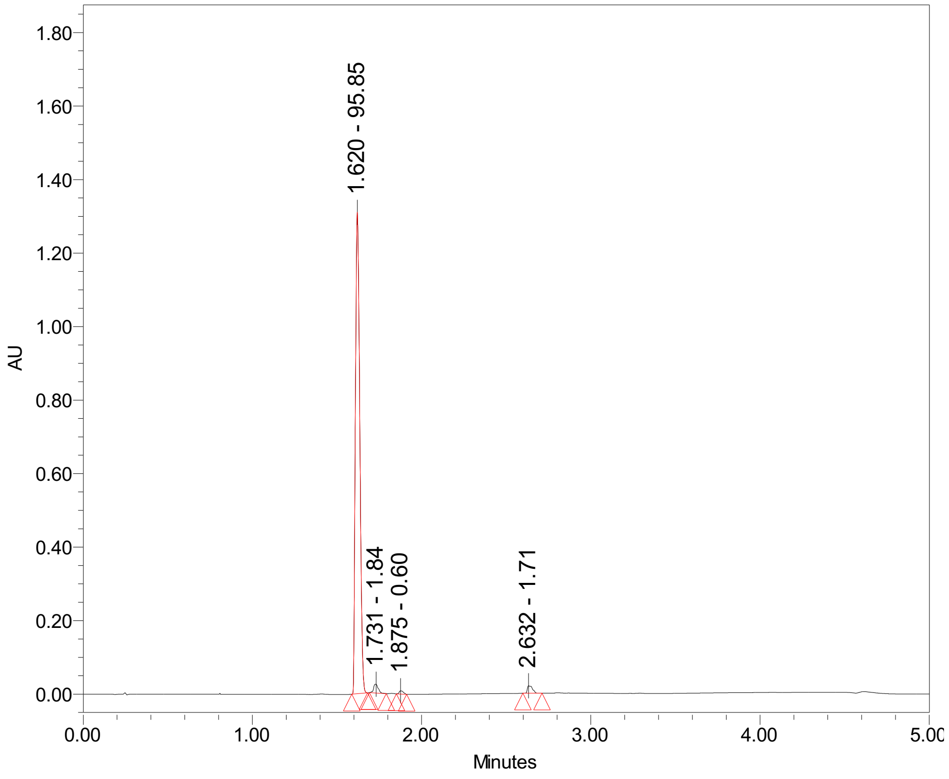

1.80
1.60
1.40
1.20
1.00
0.80
0.60
0.40
0.20
0.00
AU
1.620 - 95.85
1.731 - 1.84
1.875 - 0.60
2.632 - 1.71
0.00
1.00
2.00
3.00
4.00
5.00
Minutes

CS-1-162

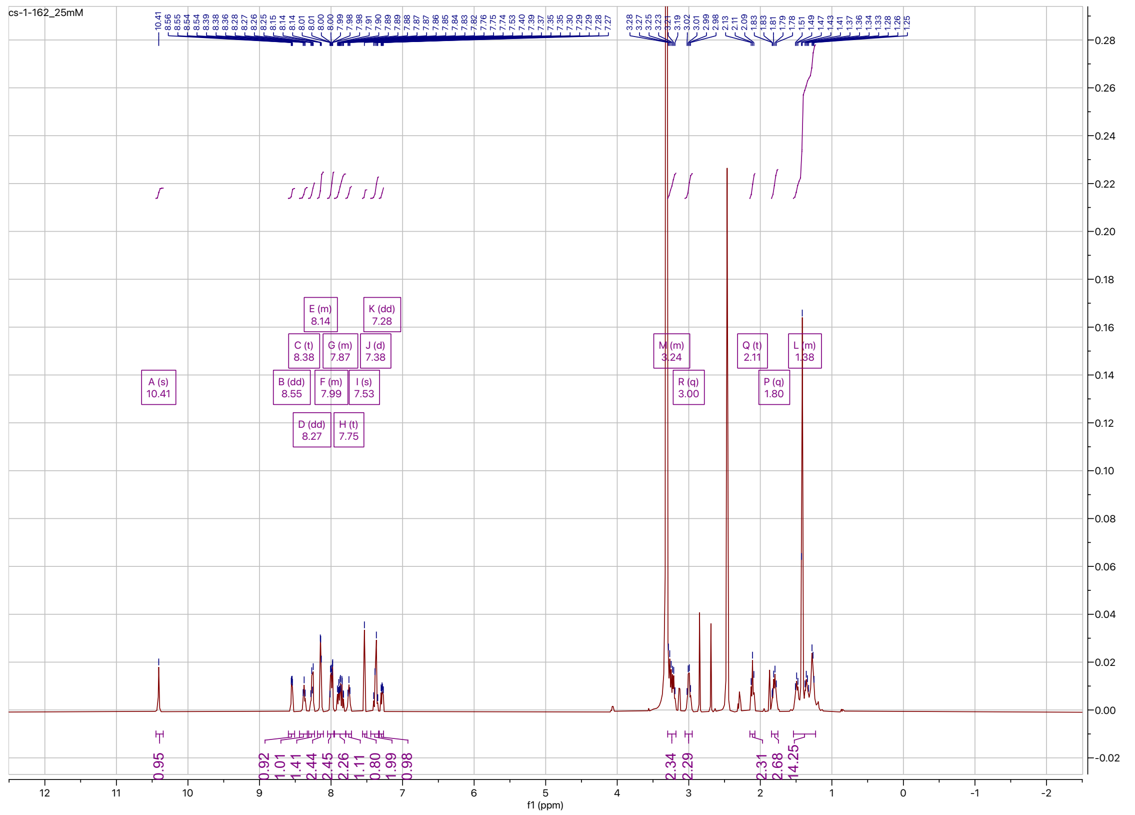

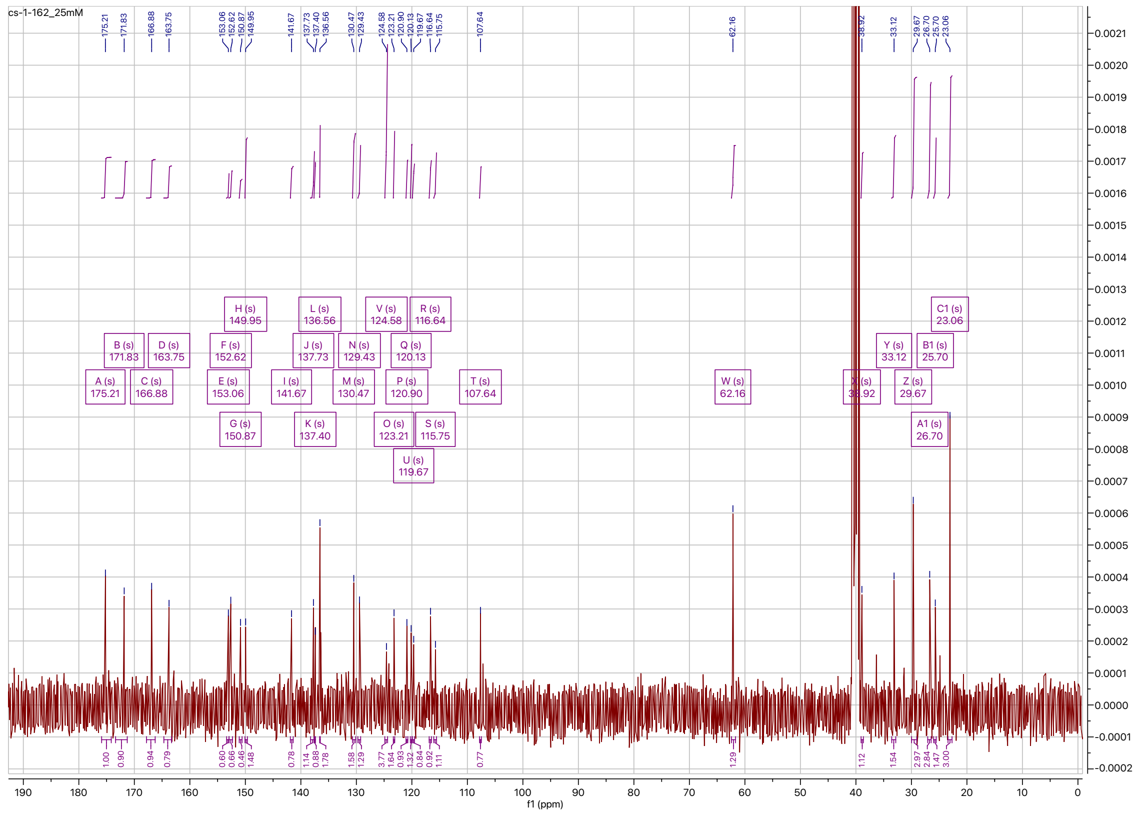

CS-1-163:

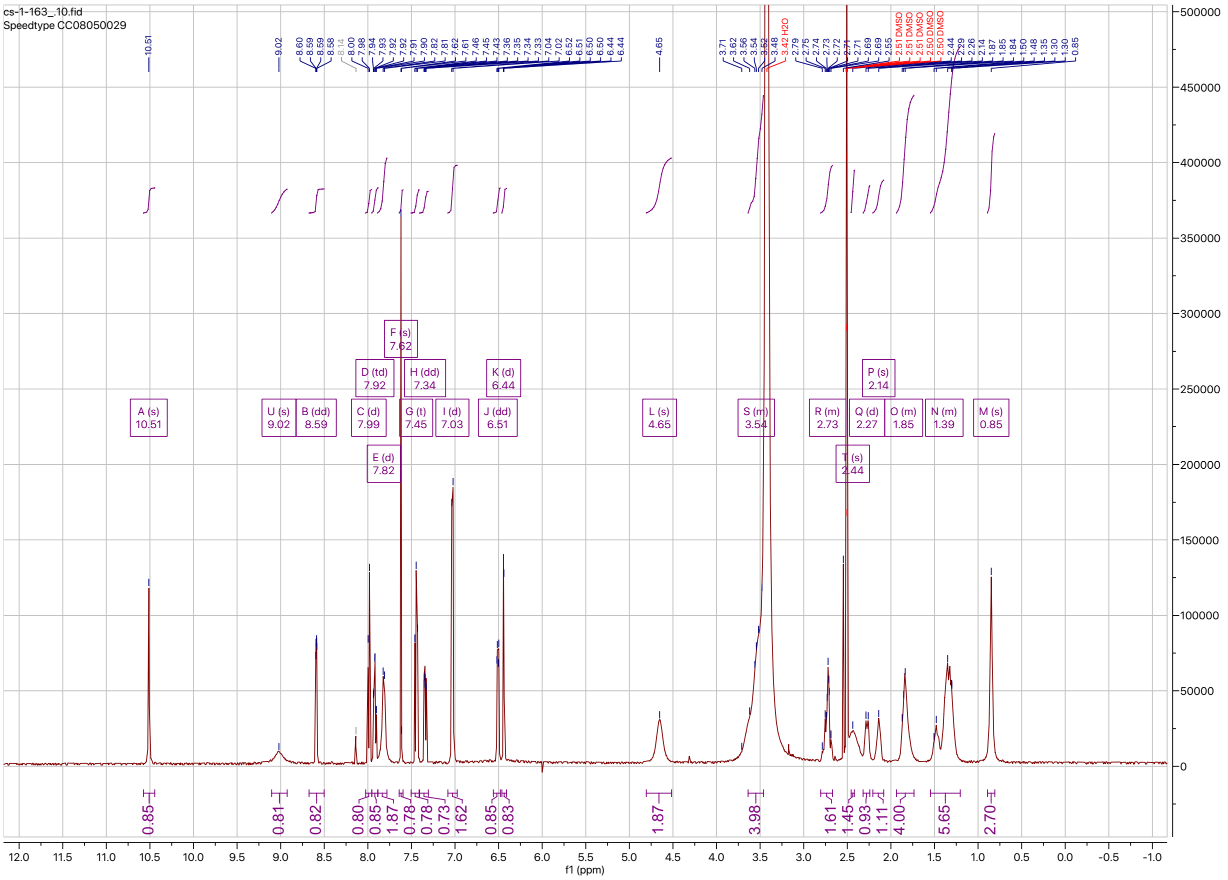

cs-1-163_.10.fid
Speedtype CC08050029
f1 (ppm)

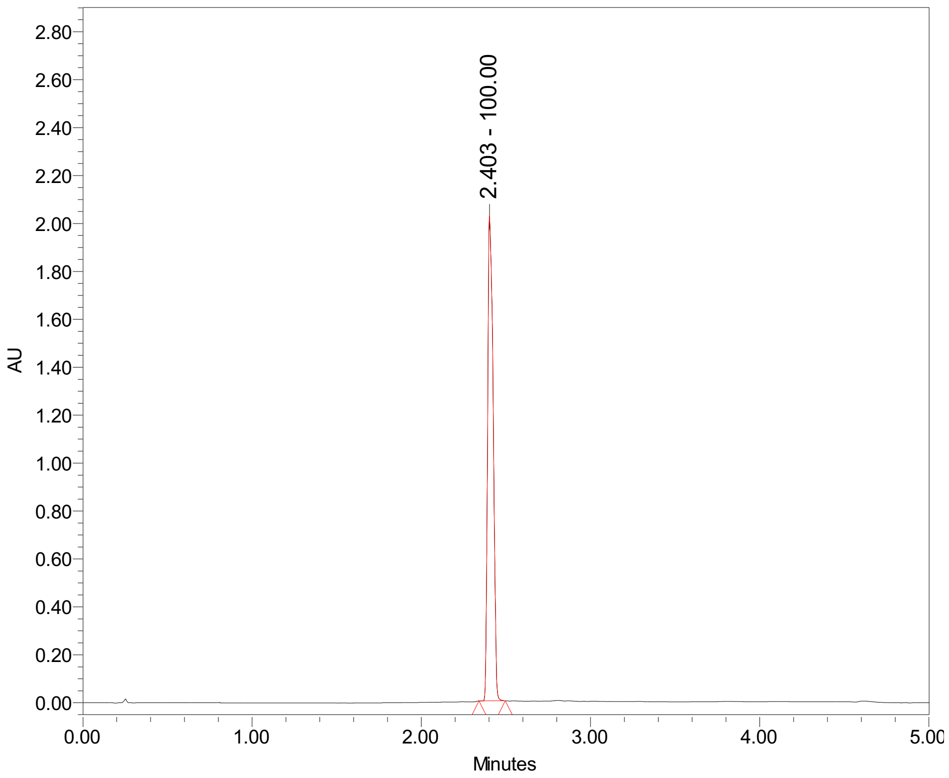

2.403 - 100.00
AU
Minutes

CS-1-164:

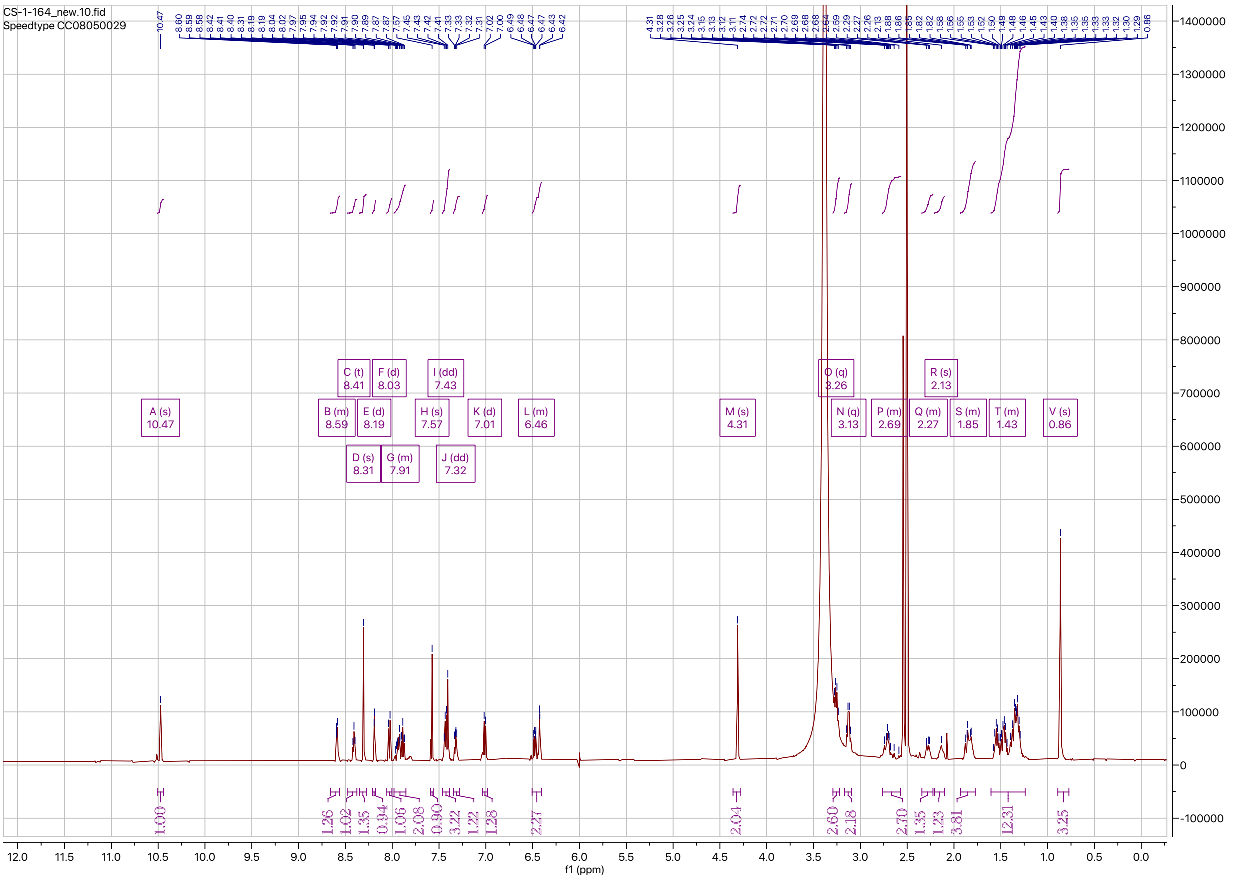

CS-1-164_new.10.fid
Speedtype CC08050029
A (s) 10.47
B (m) 8.59
C (t) 8.41
D (s) 8.31
E (d) 8.19
F (d) 8.03
G (m) 7.91
H (s) 7.57
I (dd) 7.43
J (dd) 7.32
K (d) 7.01
L (m) 6.46
M (s) 4.31
O (q) 3.26
N (q) 3.13
P (m) 2.69
Q (m) 2.27
R (s) 2.13
S (m) 1.85
T (m) 1.43
V (s) 0.86
f1 (ppm)

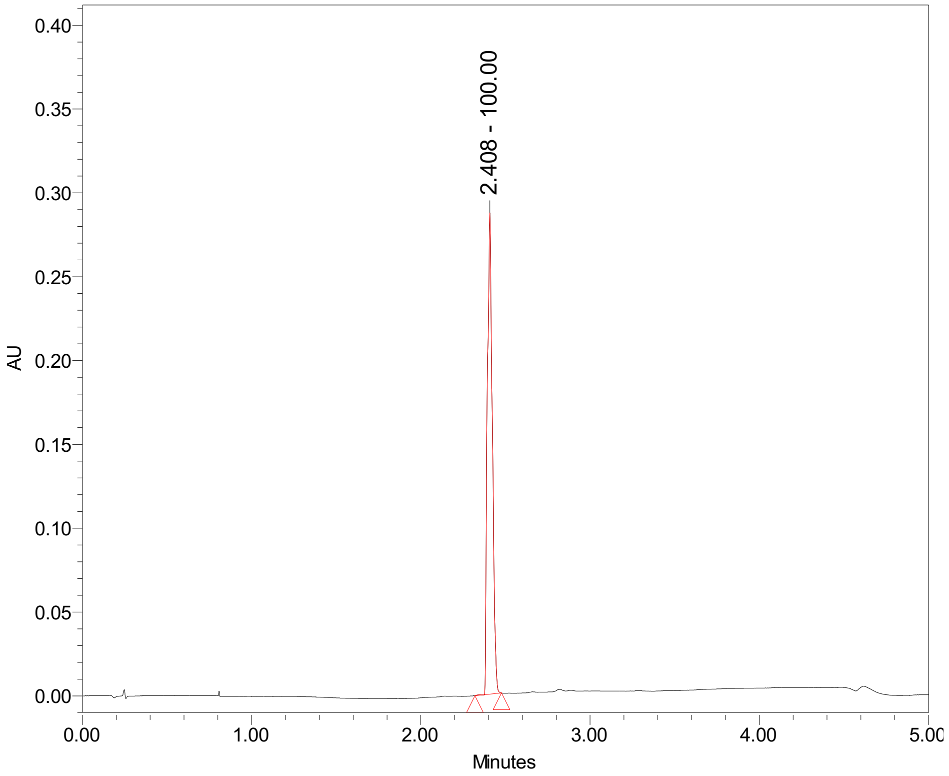

2.408 - 100.00
AU
Minutes

CS-1-165:

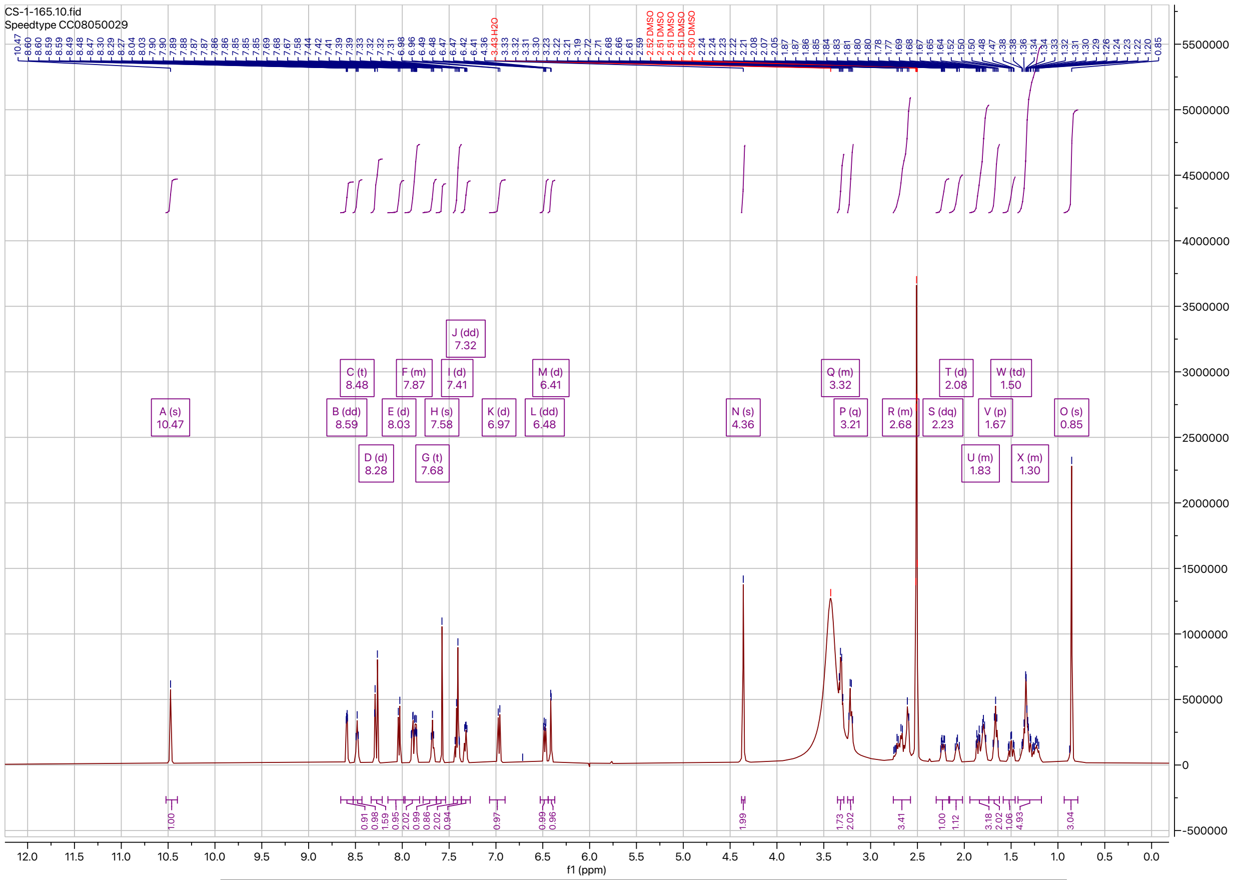
CS-1-165.10.fid
Speedtype CC08050029
A (s) 10.47
B (dd) 8.59
C (t) 8.48
D (d) 8.28
E (d) 8.03
F (m) 7.87
G (t) 7.68
H (s) 7.58
I (d) 7.41
J (dd) 7.32
K (d) 6.97
L (dd) 6.48
M (d) 6.41
N (s) 4.36
O (s) 0.85
P (q) 3.21
Q (m) 3.32
R (m) 2.68
S (dq) 2.23
T (d) 2.08
U (m) 1.83
V (p) 1.67
W (td) 1.50
X (m) 1.30
f1 (ppm)

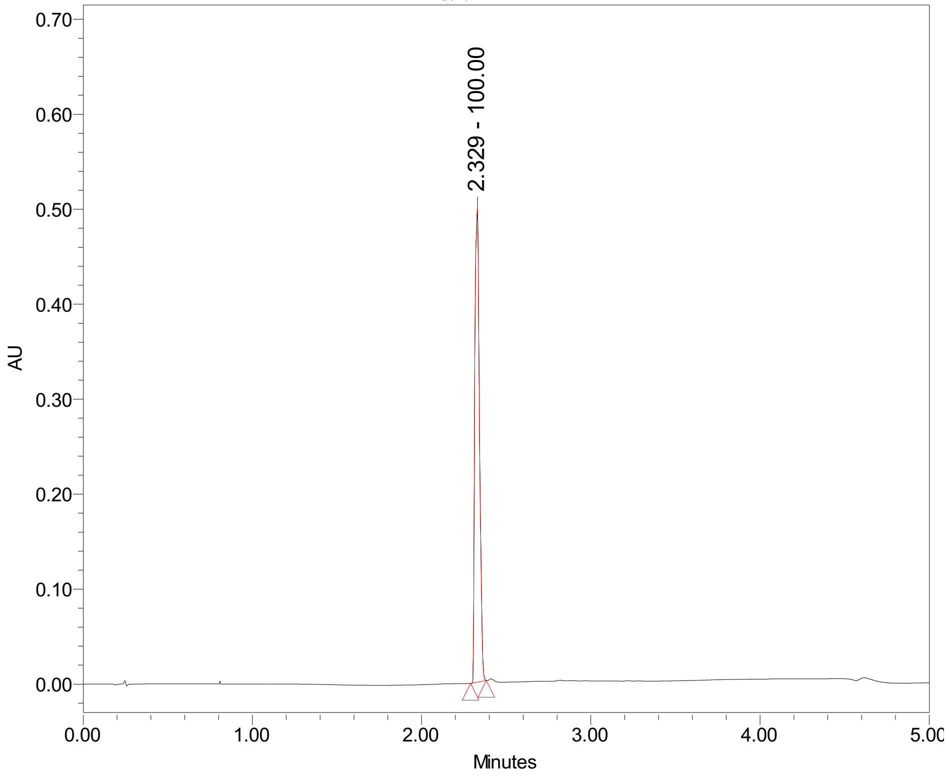
2.329 - 100.00
AU
Minutes

CS-1-166:

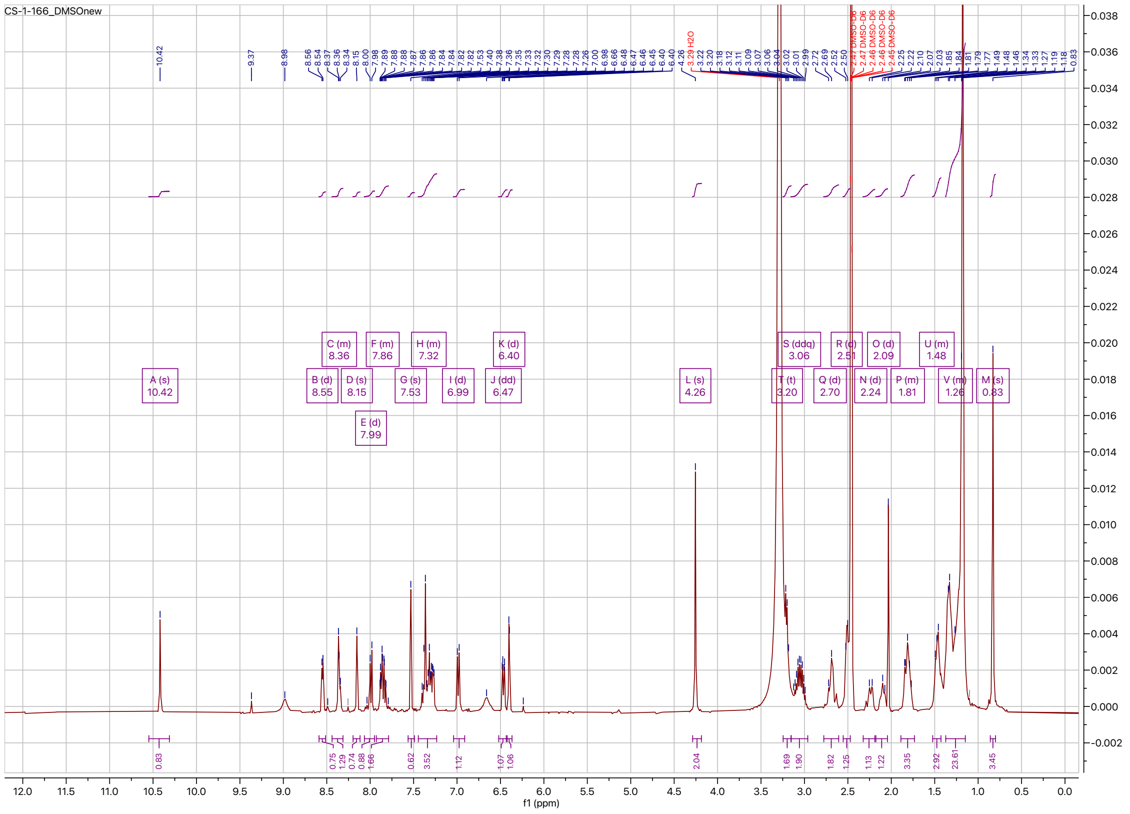

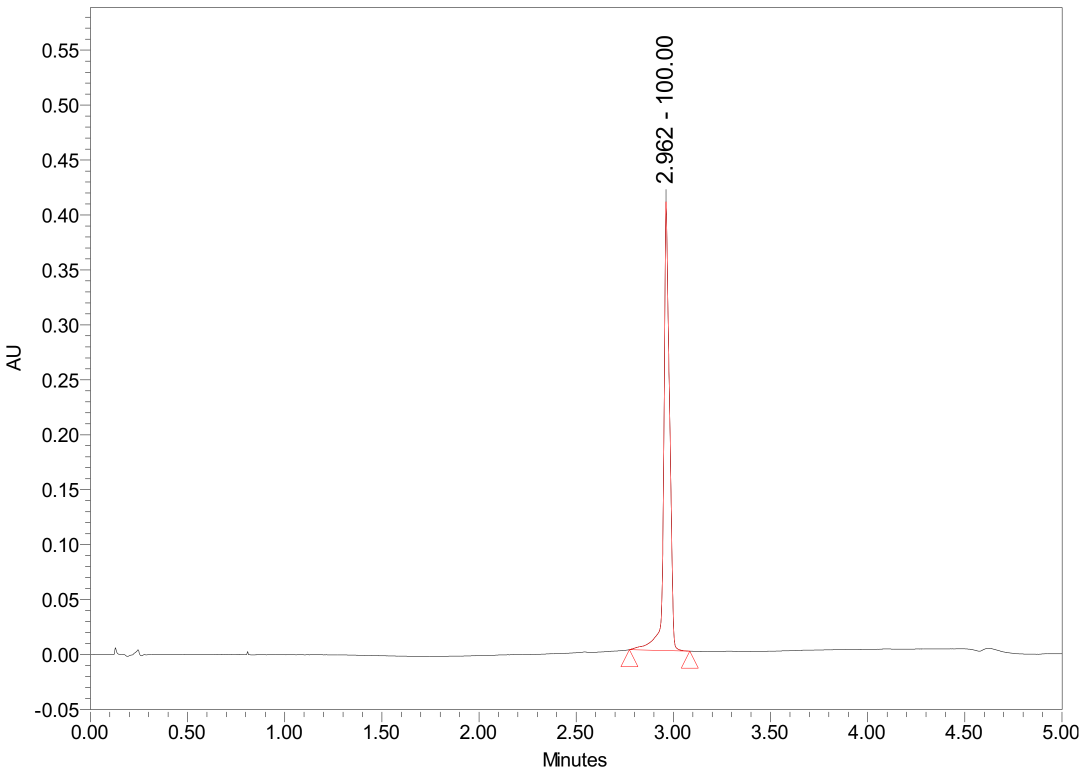

CS-1-167:

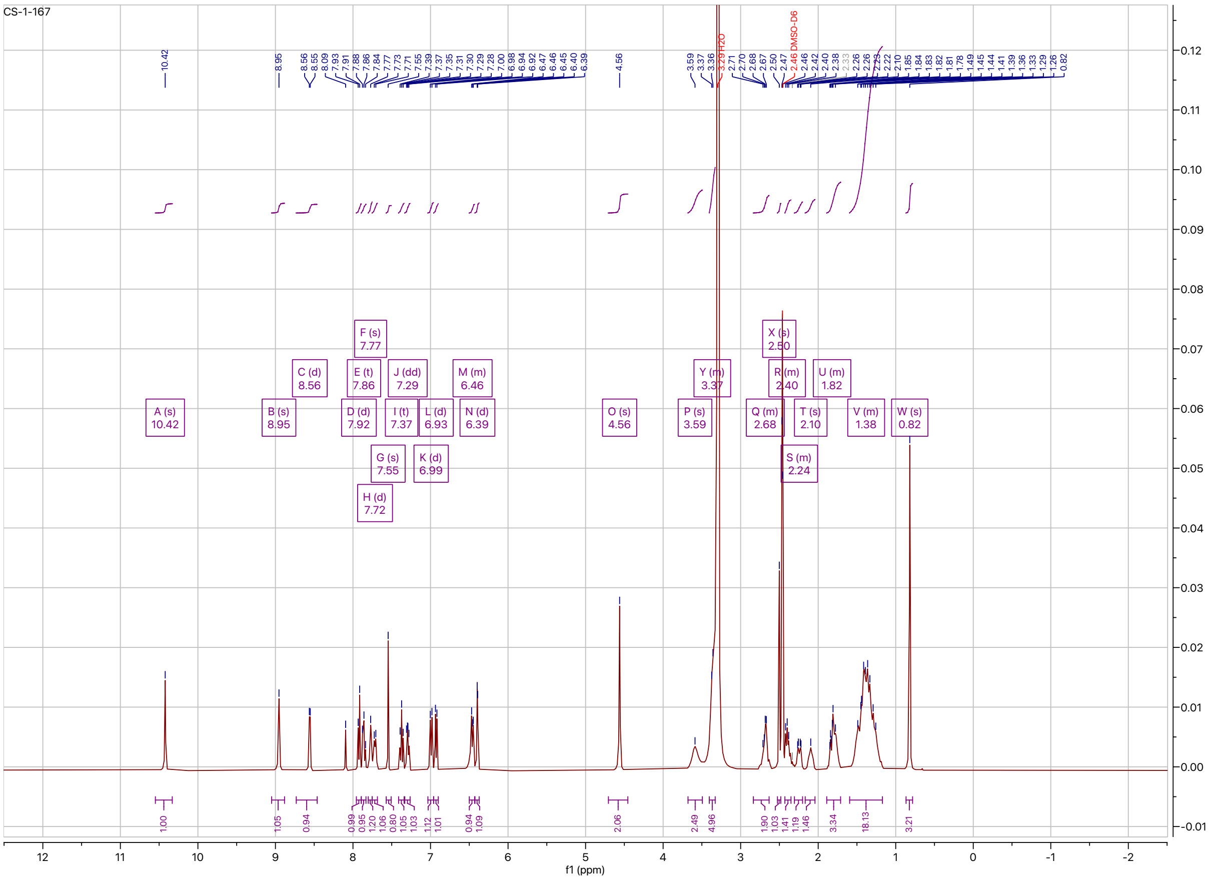

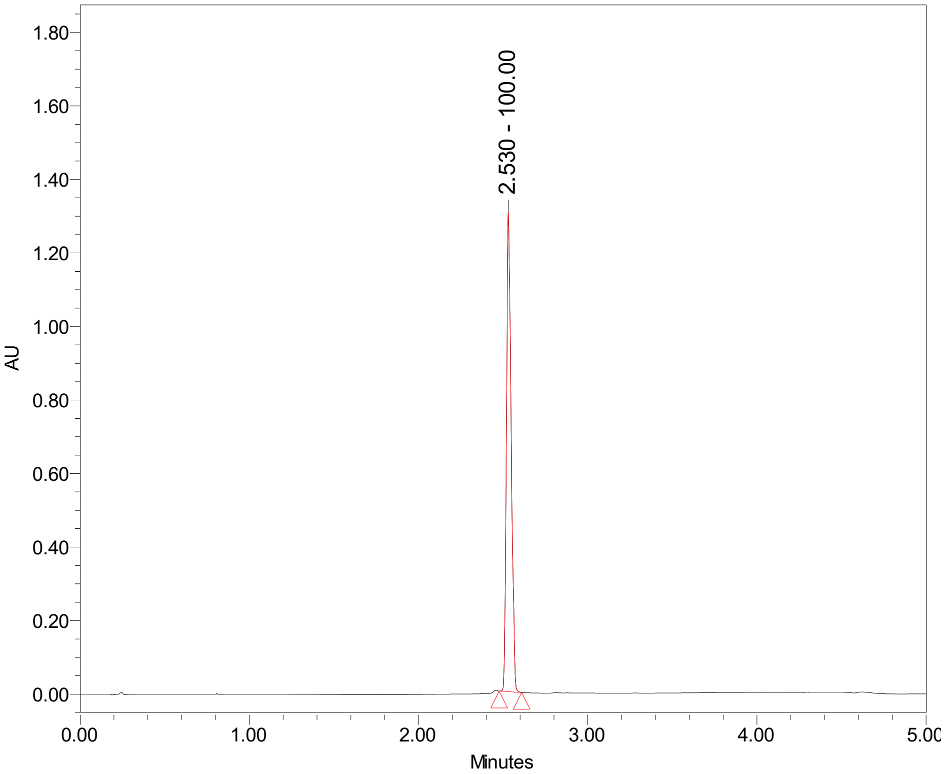

1.80
1.60
1.40
1.20
1.00
0.80
0.60
0.40
0.20
0.00
AU
2.530 - 100.00
0.00
1.00
2.00
3.00
4.00
5.00
Minutes

CS-1-168

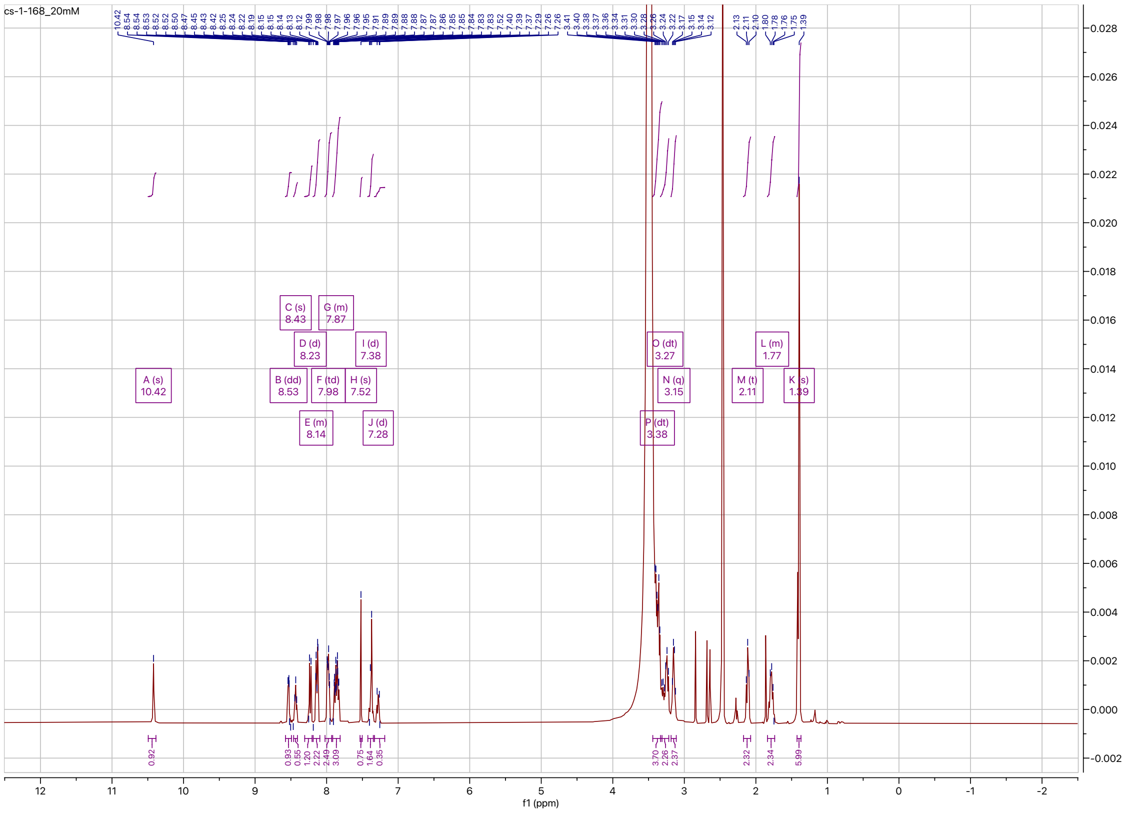
cs-1-168_20mM
A (s) 10.42
B (dd) 8.53
C (s) 8.43
D (d) 8.23
E (m) 8.14
F (td) 7.98
G (m) 7.87
H (s) 7.52
I (d) 7.38
J (d) 7.28
P (dt) 3.38
O (dt) 3.27
N (q) 3.15
M (t) 2.11
L (m) 1.77
K (s) 1.39
f1 (ppm)

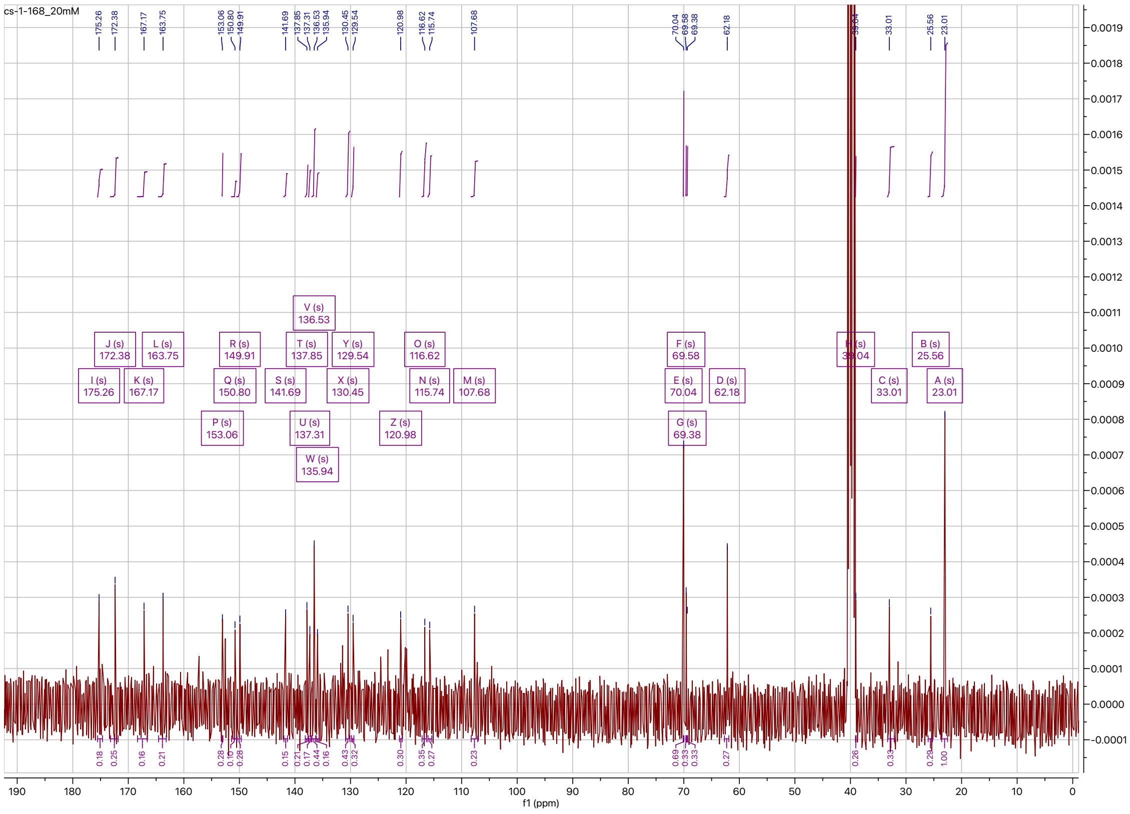
cs-1-168_20mM
I (s) 175.26
J (s) 172.38
K (s) 167.17
L (s) 163.75
P (s) 153.06
Q (s) 150.80
R (s) 149.91
S (s) 141.69
T (s) 137.85
U (s) 137.31
V (s) 136.53
W (s) 135.94
X (s) 130.45
Y (s) 129.54
Z (s) 120.98
O (s) 116.62
N (s) 115.74
M (s) 107.68
E (s) 70.04
F (s) 69.58
G (s) 69.38
D (s) 62.18
H (s) 39.04
C (s) 33.01
B (s) 25.56
A (s) 23.01
f1 (ppm)

CS-1-169

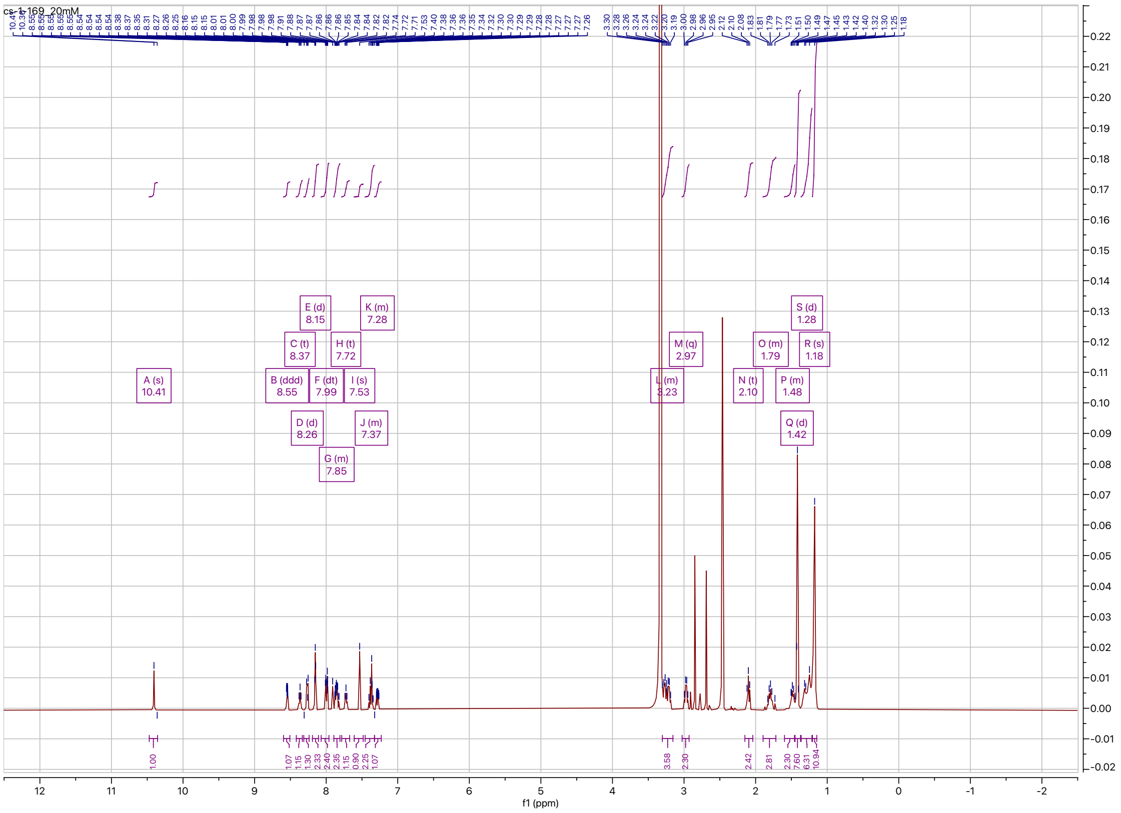

cs-1-169_20mM
A (s) 10.41
B (ddd) 8.55
C (t) 8.37
D (d) 8.26
E (d) 8.15
F (dt) 7.99
G (m) 7.85
H (t) 7.72
I (s) 7.53
J (m) 7.37
K (m) 7.28
M (q) 2.97
N (t) 2.10
O (m) 1.79
P (m) 1.48
Q (d) 1.42
R (s) 1.18
S (d) 1.28
f1 (ppm)

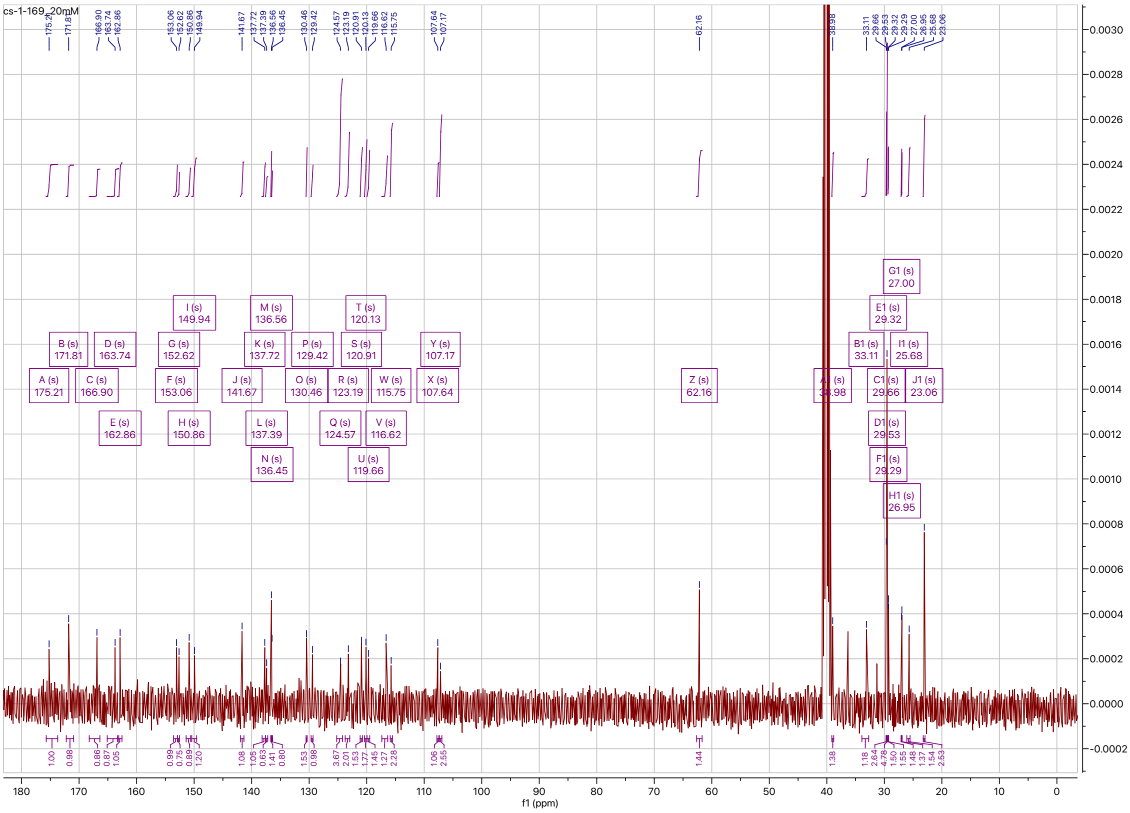

cs-1-169_20mM
A (s) 175.21
B (s) 171.81
C (s) 166.90
D (s) 163.74
E (s) 162.86
F (s) 153.06
G (s) 152.62
H (s) 150.86
I (s) 149.94
J (s) 141.67
K (s) 137.72
L (s) 137.39
M (s) 136.56
N (s) 136.45
O (s) 130.46
P (s) 129.42
Q (s) 124.57
R (s) 123.19
S (s) 120.91
T (s) 120.13
U (s) 119.66
V (s) 116.62
W (s) 115.75
X (s) 107.64
Y (s) 107.17
Z (s) 62.16
B1 (s) 33.11
C1 (s) 29.66
D1 (s) 29.53
E1 (s) 29.32
F1 (s) 29.29
G1 (s) 27.00
H1 (s) 26.95
I1 (s) 25.68
J1 (s) 23.06
f1 (ppm)

CS-1-175:

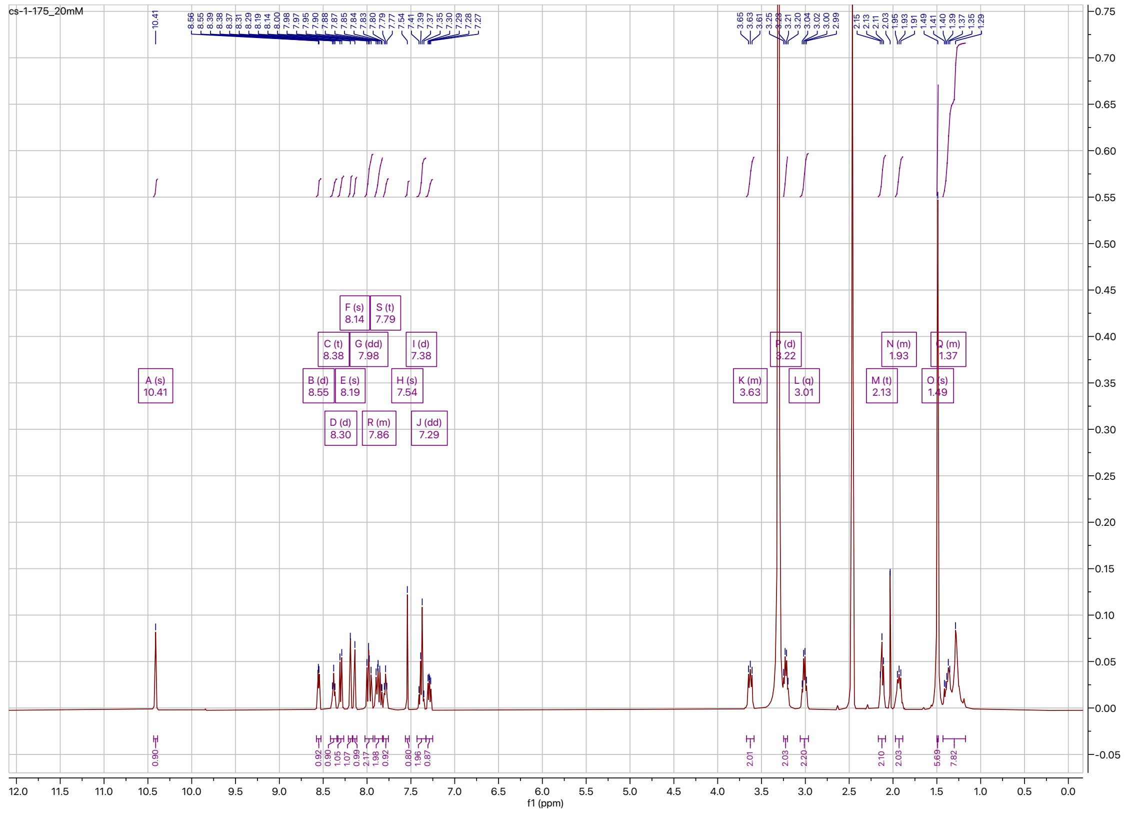

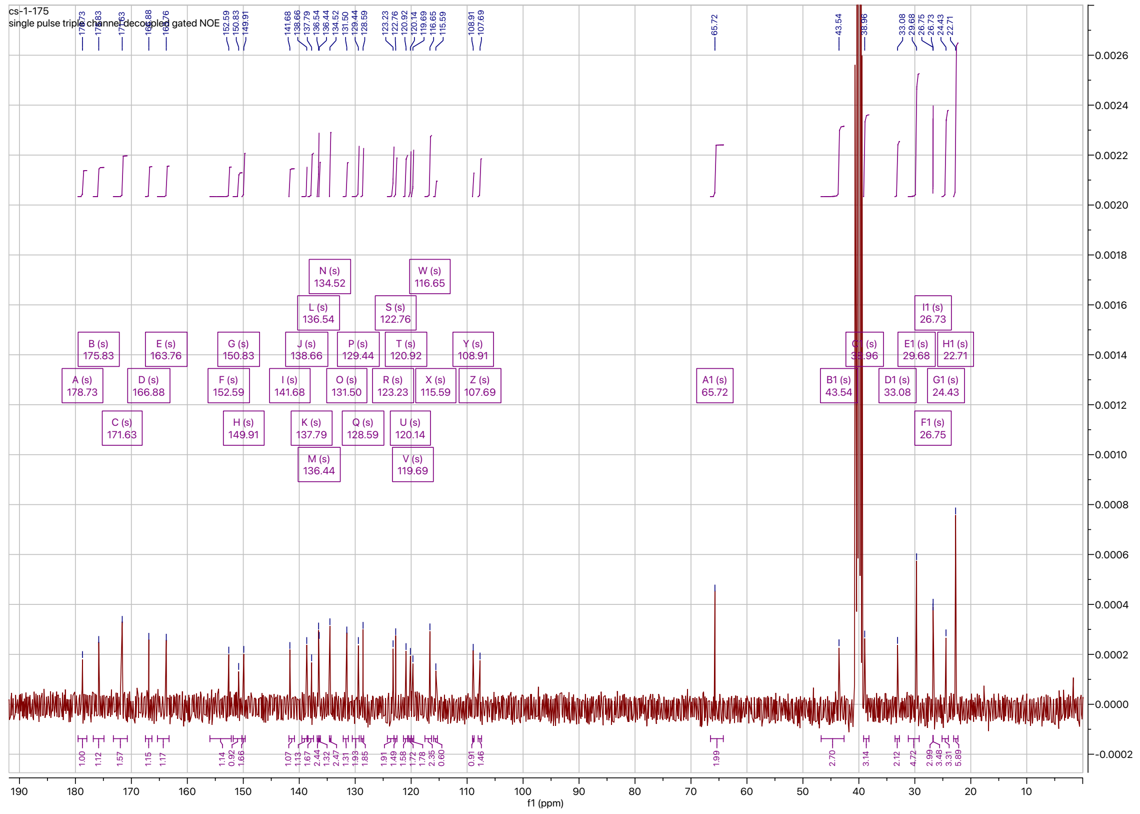

CS-1-176

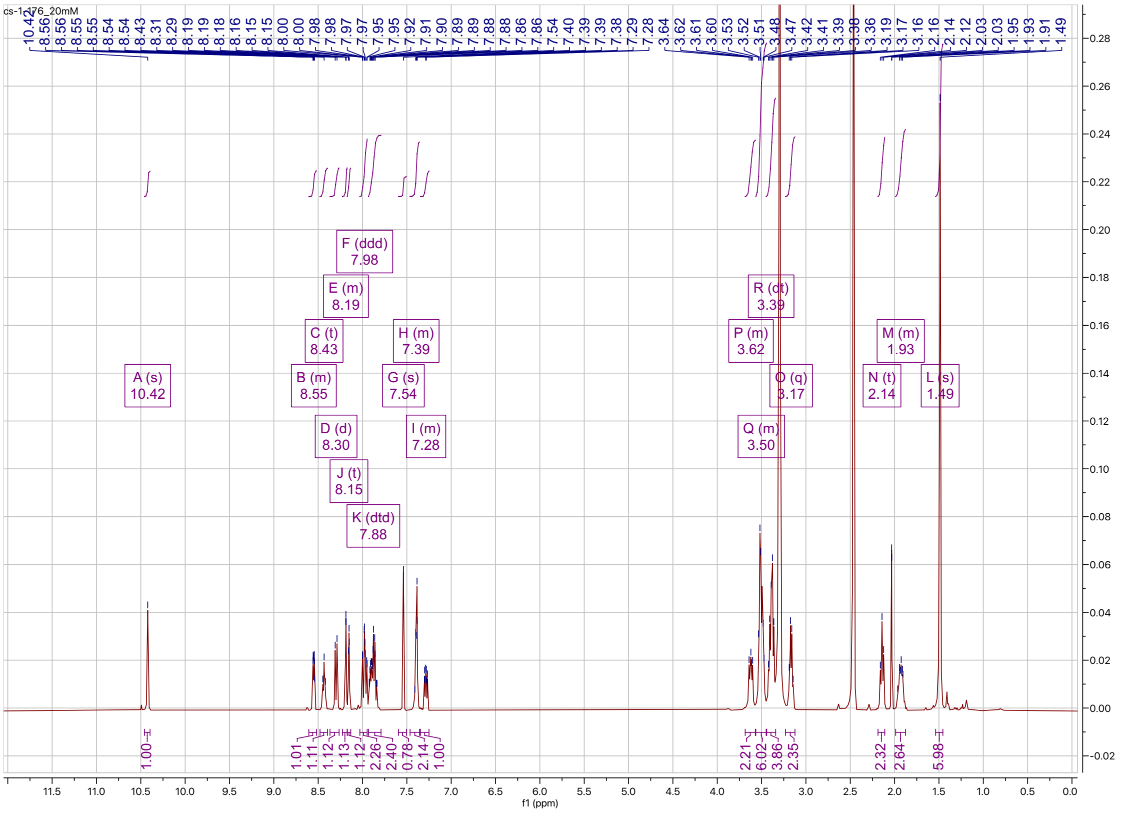

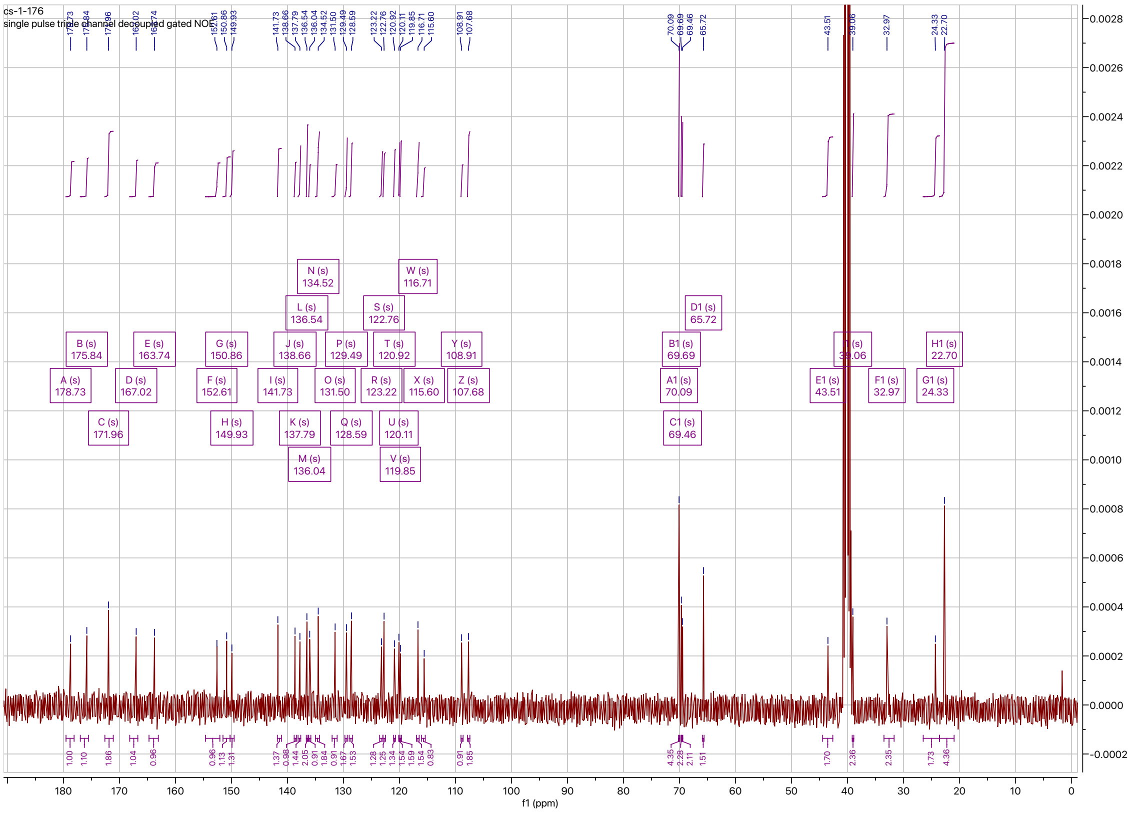

Supplement: Supplementary file 1 [file oc5c02277_si_001.pdf]
